# Re-estimation improved the performance of two Framingham cardiovascular risk equations and the Pooled Cohort equations: A nationwide registry analysis

**DOI:** 10.1038/s41598-020-64629-6

**Published:** 2020-05-18

**Authors:** Christine Wallisch, Georg Heinze, Christoph Rinner, Gerald Mundigler, Wolfgang C. Winkelmayer, Daniela Dunkler

**Affiliations:** 1 Medical University of Vienna, CEMSIIS, Section for Clinical Biometrics, Vienna, Austria; 2 Medical University of Vienna, CEMSIIS, Section for Medical Information Management, Vienna, Austria; 3 Medical University of Vienna, Department of Medicine 2, Division of Cardiology, Vienna, Austria; 4 Baylor College of Medicine, Section of Nephrology, Houston, TX USA

**Keywords:** Disease prevention, Cardiovascular diseases, Population screening

## Abstract

Equations predicting the risk of occurrence of cardiovascular disease (CVD) are used in primary care to identify high-risk individuals among the general population. To improve the predictive performance of such equations, we updated the Framingham general CVD 1991 and 2008 equations and the Pooled Cohort equations for atherosclerotic CVD within five years in a contemporary cohort of individuals who participated in the Austrian health-screening program from 2009–2014. The cohort comprised 1.7 M individuals aged 30–79 without documented CVD history. CVD was defined by hospitalization or death from cardiovascular cause. Using baseline and follow-up data, we recalibrated and re-estimated the equations. We evaluated the gain in discrimination and calibration and assessed explained variation. A five-year general CVD risk of 4.61% was observed. As expected, discrimination c-statistics increased only slightly and ranged from 0.73–0.79. The two original Framingham equations overestimated the CVD risk, whereas the original Pooled Cohort equations underestimated it. Re-estimation improved calibration of all equations adequately, especially for high-risk individuals. Half of the individuals were reclassified into another risk category using the re-estimated equations. Predictors in the re-estimated Framingham equations explained 7.37% of the variation, whereas the Pooled Cohort equations explained 5.81%. Age was the most important predictor.

## Introduction

Cardiovascular disease (CVD) remains the leading cause of morbidity and death in developed countries. CVD strongly relates to life-style and other potentially modifiable risk factors, but atherosclerosis, usually the underlying pathology, progresses over many years without symptoms. CVD risk equations are used in primary care to identify high-risk individuals. However, an abundance of CVD equations already exists; e.g., in a systematic review Damen *et al*. found 363 CVD equations for the general population^1^.

Consequently, instead of developing new equations, research should utilize available evidence by focusing on validation and updating of promising, existing equations^1,2^. External validation is conducted often for CVD equations^3–5^, but updating studies are less frequent^6–8^. External validation studies often show severe under- or overprediction as the incidence of the outcome and the distributions of risk factors differ across populations^9,10^. Generally, risk equations will overestimate risk if applied to a lower risk population, and underestimate it if applied to a higher risk population. Damen *et al*. also confirmed the need for updating of equations as miscalibration varies across settings^11^.

We externally validated three well-known CVD equations—the 1991 and 2008 Framingham general CVD equations (FR1991 and FR2008 equations) and the Pooled Cohort (PC) equation for atherosclerotic CVD (ASCVD)—for occurrence of (AS)CVD within five years in a large contemporary cohort of 1.7 M participants of the Austrian health-screening program^12–16^. This program offers a yearly, standardized and free-of-charge preventive health-screening to Austrian residents, a country with mandatory and near-universal health care coverage^17^. During a health-screening, the risk of an non-fatal or fatal CVD event in the next five years is predicted for each participant using the New Zealand Risk Scale and the American Heart Association Risk Calculator^18,19^. The SCORE equation focusing only on CVD death is not considered, as the combined endpoint of non-fatal and fatal CVD is more relevant for individuals in this setting^20^.

In the Austrian study cohort, we found that both Framingham equations slightly overestimated the five-year risk, but underestimated the risk of individuals older than 65 years, whereas the PC equations systematically underestimated the five-year risk. Consequently, to improve the predictive performance of these equations in the Austrian and similar populations, we sought to update these equations utilizing contemporary data of a nationwide registry.

## Methods

### Updating of risk equations

Equations are updated by enhancing information from the development cohorts captured in the original equations with information from a new cohort. In the present study, updating is achieved by recalibration and re-estimation^21,22^. A recalibrated equation corrects ‘calibration-in-the-large’, which means aligning the mean predicted probability to the observed outcome frequency in the new cohort. Re-estimation refers to the updating of the original regression coefficients with new data to adjust the equation to local and contemporary circumstances. More extensive updating methods are often not required^11^.

### Evaluated risk equations

The FR1991 equation is the best known CVD prediction tool for combined non-fatal and fatal CVD. The small development cohort had a high background CVD risk, and was mostly untreated as was standard of care in the late 1960s (the time of study entry). The FR2008 equation is an update of the 1991 equation based on an extended cohort. In the Framingham equations, CVD is defined as coronary heart disease (coronary death, myocardial infarction, coronary insufficiency, angina), cerebrovascular events (ischemic stroke, hemorrhagic stroke, transient ischemic attack), peripheral artery disease (intermittent claudication), and heart failure. The PC equations were derived from pooled US cohorts and focus on ASCVD, defined as nonfatal myocardial infarction or coronary heart disease death, or stroke. We applied the PC equations for non-Hispanic whites. Only traditional CVD risk factors are used in these equations comprising sex, age, blood pressure (BP), cholesterol, diabetes, and smoking.

### The Austrian health-screening program

Around 13% of the relevant population participates in the preventive health-screening program every year (e.g., 2011: n = 884,589, 12.8%)^23^. On average, people attend a health-screening every three years, which means that more than one third of the screening population is reached. A main goal of the program is the reduction of the incidence and mortality of CVD. During a health-screening, the absolute 5-year CVD risk is assessed using risk tables^18,19^; for participants up to 39 years the AHA risk calculator resulting in three risk categories and for older participants the New Zealand risk scale leading to four risk categories are currently applied. Clinically determined high-risk factors like diabetes (for the AHA risk calculator) and CVD in the family anamnesis (for the New Zealand Risk Scale) are additionally taken into consideration. The risk tables are shown to and discussed with the participants to explain the individual risk profile and to educate on important risk factors. Depending on the assessed risk, medical practioners may recommend and initiate various preventive interventions outlined in the guidelines of the program^23,24^.

### Study population

**Figure 1:**
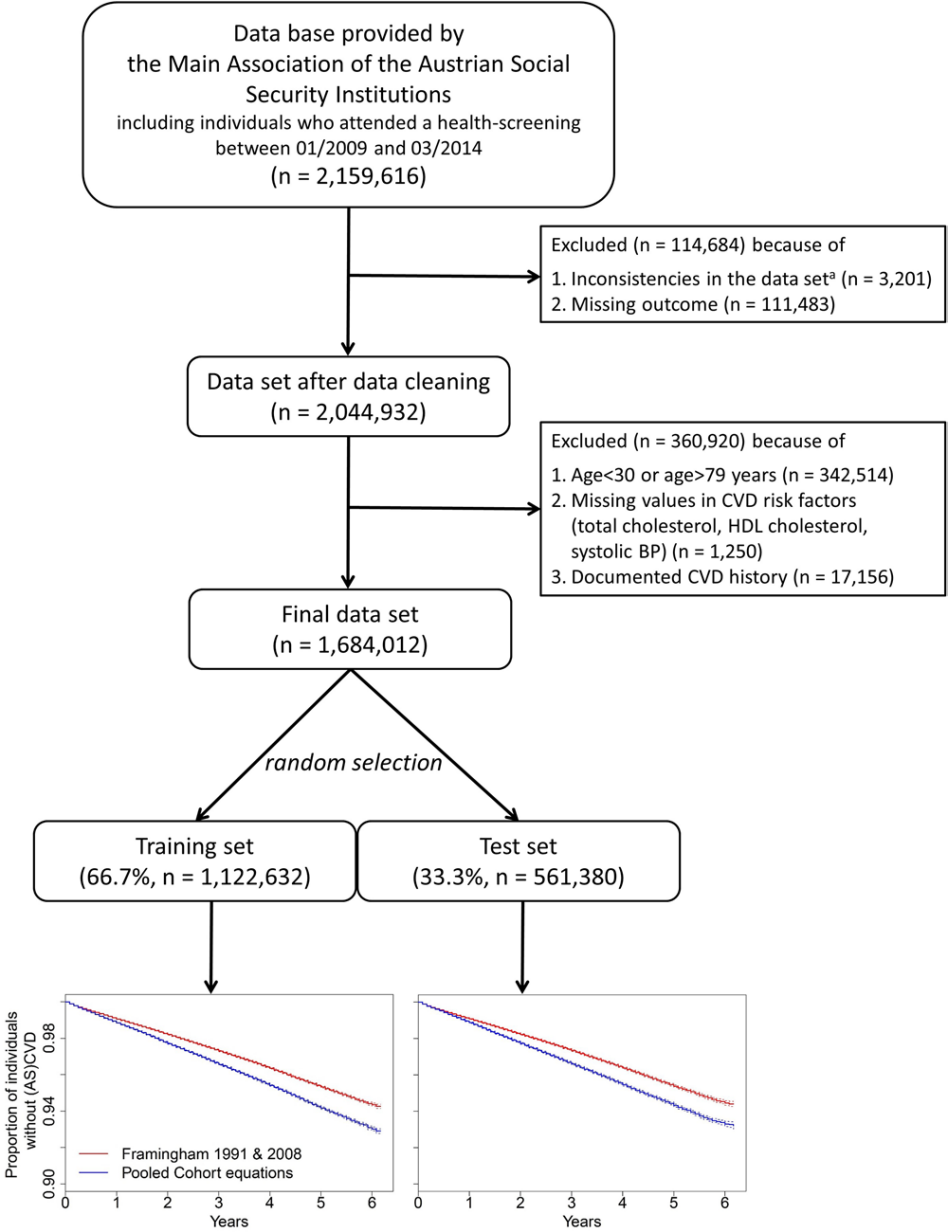
Flow-chart deriving the training and test set. At the bottom, Kaplan-Meier plots for the time to a first (atherosclerotic-) cardiovascular disease for the evaluated equations in the training and the test set are shown. ^a^Individuals with inconsistencies in repeated health-screenings (sex, date of birth, or death) were excluded.

A pseudonymized database was provided by the Main Association of the Austrian Social Security Institutions containing all preventive health-screenings in Austria. A description of the data preparation can be found in Supplementary Methods S1. The database included information on hospitalizations (1/2008-3/2015), health-screenings (1/2009-3/2014), and causes of death (1/2009-3/2015). At health-screenings, general practioners collected standardized information on individuals, e.g., demographics, laboratory values, BP and related medication, smoking status, and comorbidities like diabetes. Missing values were very rarely observed (Fig. 1). To ascertain CVD outcomes, information on hospitalizations, and primary causes of death classified according to the 9th or 10th revision of the International Classification of Diseases (ICD) were used. As the definitions of CVD differ slightly between the investigated equations, different ICD-codes were required (Supplementary Tables S2a-c). Causes of death were obtained by linkage with the registry of deaths from Statistik Austria, the Austrian Federal Institute for Statistics. For 10.3% of the combined outcome a probabilistic assignment of CVD/non-CVD-related death was applied (Supplementary Methods S1). Individuals were followed-up until the date of their first CVD-related hospitalization, the date of fatal CVD, or 3/15/2015, whatever occurred first.

While the equations were developed for a time horizon of ten years, we mainly focused on five-year risk because of data availability. However, assuming a constant hazard, the predicted five-year risk can be used to approximate a risk prediction for ten years (for details see Supplementary Fig. 1). The five-year risk is roughly half the ten-year risk (for ten-year risk up to 20%). Considering constant hazards is the most plausible assumption given that the observational period did not start with an intervention such as a surgery or diagnosis of a disease. The plots of the hazard for (AS-)CVD in Supplementary Fig. 1 confirm that the assumption of constant hazards is approximately fulfilled for the first five years. For the FR1991 equation, the five and ten-year risk can be directly estimated. For the original FR2008 equation, we assumed a constant hazard to approximate the five-year baseline survival function, and for the PC equations, the five-year baseline survival was described in Muntner *et al*.^4^. In the FR1991 equation, we did not consider an indicator for left ventricular hypertrophy, which has a very low prevalence (0.8%)^25^, as in the New Zealand Risk Scale^19^. The final study cohort comprised 1,684,012 individuals between 30 and 79 years at their first health-screening without documented CVD (in a time window of one year prior to the health-screening), and with a potential follow-up of at least one year. Statin therapy at the time of the health-screening was not considered. This is fully consistent with the evaluated risk equations, which did not consider statin use as an inclusion/exclusion criterion or a candidate variable. This cohort was randomly split into a training (66.7%, n = 1,122,632) and a test set (33.3%, n = 561,380) for split-sample validation, the recommended validation method for a data-rich situation^26^. The inclusion/exclusion criteria of the equations differ slightly regarding age limits and pre-existing diseases (Supplementary Tables S2a–c). Therefore, appropriate subsets of the final cohort were used for each equation. The study was approved by the ethics committee of the Medical University of Vienna (no. 1232/2014).

### Statistical analysis

Continuous variables were summarized using median and interquartile range (IQR); for categorical variables absolute frequencies and percentages were used. Mean linear predictors were compared between the study and the development cohorts to investigate their overall relatedness, i.e., the similarity in overall predicted outcome frequencies^27^. The equations were updated; each equation was *recalibrated* by re-shifting the baseline risk and *re-estimated*^2^. The FR1991 equation, which is a parametric Weibull accelerated failure time model with varying location and dispersion, could only be re-estimated.

First, the training set was used to recalibrate and re-estimate the equations. Second, in the independent test set performance of the recalibrated and the re-estimated equations was evaluated and compared with results from the original equations. Hence, all results except the recalibrated and re-estimated regression coefficients were derived from the test set. The performance of the original, the recalibrated and the re-estimated equations was assessed by evaluating discrimination using c-statistics^28^, and calibration examining calibration slope, calibration-in-the-large, and calibration plots. The calibration slope is the regression coefficient of a univariable model with CVD as outcome and the centered linear predictor as the independent variable. Calibration-in-the-large is the difference of the mean predicted risk and the observed cumulative incidence. Calibration plots display probabilities as predicted by the equations and the corresponding observed cumulative incidences for deciles of predicted probabilities. For re-estimated equations, explained variation of different predictors was assessed^29^. 95%-confidence intervals for c-statistics and explained variation were estimated from 1000 bootstrap samples with the percentile method. Additionally, the performance of the equations in high-risk groups encompassing the elderly, individuals with diabetes or hypertension at baseline were examined. Risk reclassifications tables were obtained to highlight movement of participants between risk categories. To investigate the generalizability of the study cohort to the general Austrian population, data from the latest Austrian Health Interview Survey (2014)^30^, a representative Austrian-wide survey, were used. Re-estimated regression coefficients, baseline survival and mean linear predictors can be found in Supplementary Tables S2a–c.

## Results

### Baseline characteristics

Baseline characteristics of 1,684,012 individuals in the study cohort are detailed in Table 1. Individuals in the training set (n = 1,122,632) had almost identical baseline characteristics to individuals in the test set (Supplementary Table S3). The median age was 50 (IQR 41, 62) with 53.8% being female. Women reached more frequently recommended target values in high-density lipoprotein (HDL) cholesterol, had lower systolic BP, smoked less, and had a lower prevalence of diabetes. CVD risk is primarily assessed in individuals who attend health-screenings, matching this study cohort. Nonetheless, we wondered if equations updated with information from this cohort may also be applicable to the general Austrian population. First, our study cohort included participants from all Austrian provinces and represented 32.5% (n = 8,388,534; 2011) of all Austrian inhabitants between 30 and 79 years. Second, comparing baseline characteristics of this cohort to the latest Austrian Health Interview Survey showed that characteristics of individuals attending health-screenings are representative of the general population regarding the distribution of sex, smoking habits, and diabetes (Supplementary Table S4). However, in the study cohort women older than 70 were underrepresented, and especially individuals older than 60 had a slightly better five-year overall survival compared to the general population^31^.

**Table 1:** Baseline characteristics of 1,684,012 individuals in the study cohort.

Baseline characteristics	Women (n = 905,806, 53.8%)	Men (n = 778,206, 46.2%)
Age, median (IQR), years	50 (41, 62)	50 (41, 61)
Total cholesterol, median (IQR), mmol/L	5.40 (4.73, 6.15)	5.35 (4.65, 6.08)
HDL cholesterol (mmol/L), median (IQR), mmol/L	1.60 (1.34, 1.91)	1.27 (1.06, 1.53)
Cholesterol ratio (total/HDL cholesterol), median (IQR), mmol/L	3.3 (2.7, 4.1)	4.2 (3.4, 5.1)
Systolic BP (mmHg), median (IQR), mmol/L	125 (115, 140)	130 (120, 141)
BP treatment, no. (%)	127,529 (14.1)	119,239 (15.3)
Smoking, no. (%)	182,808 (20.2)	187,896 (24.1)
Diabetes, no. (%)	40,293 (4.5)	48,910 (6.3)
**Observed five-year risk**^**a**^ **in % for CVD as defined by**		
Framingham 1991 CVD (56,380 events)^b^	3.29	6.17
Framingham 2008 CVD (55,078 events)^b^	3.31	6.25
Pooled Cohort ASCVD **e**quations (60,219 events)	4.32	7.49

Abbreviations: ASCVD, atherosclerotic cardiovascular disease; BP, blood pressure; CVD, cardiovascular disease; HDL, high density lipoprotein, IQR, interquartile range.

^a^Kaplan-Meier estimate. ^b^The two Framingham equations have slightly different inclusion/exclusion criteria (see Supplementary Table S2a,b).

Individuals attending health-screenings between 1/2009 and 3/2014, aged 30 to 79, without a history of CVD, and their observed five-year general and atherosclerotic CVD risk. Baseline characteristics of individuals in the training set (n = 1,122,632) compared to individuals in the test set (n = 561,380) are almost identical (Supplementary Table S3).

Compared to the development cohorts of the two Framingham equations, individuals in this study cohort had comparable risk factor distributions, thus, also mean linear predictors were similar (Supplementary Table S2a–c). Only the proportion of smokers was lower in the study cohort than in the Framingham cohorts.

### CVD and ASCVD outcome

While we observed a five-year general CVD risk of 4.58% and 4.63% according to the FR1991 and FR2008 CVD definitions; the five-year ASCVD risk was 5.69%. Men had a considerably higher five-year CVD and ASCVD risk (Table 1). Median follow-up time for the Framingham equations was 4.00 years (IQR 2.66, 5.00), and for the PC equations it was 4.08 years (2.75, 5.00).

### Updating changed some regression coefficients

**Figure 2:**
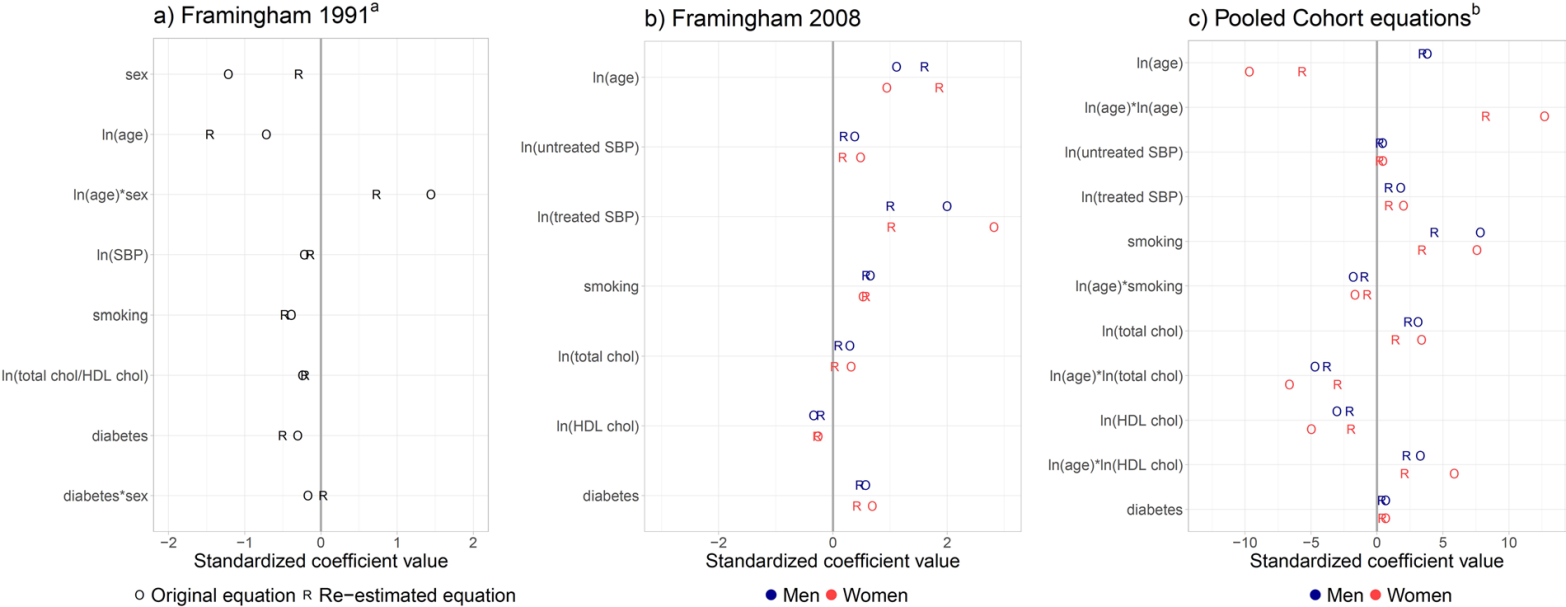
Forest-plot comparing standardized re-estimated (R) to standardized original (O) regression coefficients. Note, the different scale of standardized regression coefficients in the last panel. ^a^’ln(age)’ represents the natural logarithm of age; ‘ln(age)*sex’ is a pair-wise interaction of ln(age) with sex. ^b^The equation for men does not include a term for ‘ln(age)*ln(age)’.

The standardized re-estimated regression coefficients differed, sometimes considerably, from the original coefficients in all predictors and equations (Fig. 2). The differences reached statistical significance (also due to the large sample size) in all predictors and equations. In the Framingham equations the impact of age increased strongly. The impact of sex, and the pairwise interaction with age became less important in the FR1991 equation, whereas the effect of treated BP was reduced in the FR2008 equation. The impact of other predictors remained stable in both equations. In the PC equations a decreased effect of all predictors was observed. The reductions were most pronounced in the effect of age for women and in the effect of smoking for women and men.

### Updating slightly improved discrimination

By re-estimating the equations, c-statistics increased slightly in the study cohort (mean change 0.01; Table 2). In the re-estimated equations, c-statistics were between 0.725 and 0.787, with the two Framingham equations reaching higher c-statistics compared to the PC equations. Equations always achieved higher c-statistics for women (mean 0.778) than for men (mean 0.750).

**Table 2:** C-statistics and explained variation for the five-year risk.

Equation	Framingham 1991 CVD equation		Framingham 2008 CVD equation		Pooled Cohort ASCVD equations	
	C-statistic (95%-confidence interval)					
	women	men	women	men	women	men
original	0.769 (0.764, 0.774)	0.755 (0.751, 0.759)	0.768 (0.762, 0.772)	0.759 (0.755, 0.763)	0.776 (0.771, 0.781)	0.715 (0.711, 0.720)
re-estimated	0.784 (0.780, 0.789)	0.770 (0.766, 0.774)	0.787 (0.782, 0.792)	0.773 (0.768, 0.776)	0.782 (0.777, 0.787)	0.725 (0.721, 0.729)
difference	0.016 (0.014, 0.018)	0.015 (0.013, 0.017)	0.020 (0.017, 0.022)	0.014 (0.012, 0.016)	0.005 (0.004, 0.006)	0.010 (0.009, 0.011)
**Predictors**	**Explained variation (in % and 95%-confidence interval)**					
	**all**		**women**	**men**	**women**	**men**
*All*	7.424 (6.139, 8.790)		7.727 (5.671, 9.886)	6.971 (5.461, 8.501)	6.919 (4.258, 12.258)	4.697 (3.152, 6.233)
Age	5.645 (5.368, 5.915)		3.323 (2.954, 3.638)	5.446 (5.050, 5.799)	6.638 (3.912, 6.910)	4.012 (3.711, 4.326)
Sex	0.517 (0.436, 0.604)		—	—	—	—
Diabetes	0.399 (0.339, 0.459)		0.162 (0.098, 0.227)	0.262 (0.199, 0.326)	0.289 (0.121, 0.333)	0.141 (0.089, 0.189)
Cholesterol^a^	0.237 (0.190, 0.289)		0.299 (0.207, 0.389)	0.218 (0.153,0.285)	0.436 (0.237, 0.505)	0.175 (0.119, 0.231)
Smoking	0.214 (0.150, 0.279)		0.099 (0.007, 0.191)	0.274 (0.175, 0.363)	0.004 (−0.037, 0.069)	0.070 (0.008, 0.134)
Systolic BP^b^	0.021 (−0.020, 0.064)		0.243 (0.154, 0.333)	0.463 (0.388, 0.542)	0.416 (0.211, 0.471)	0.394 (0.326, 0.457)

Recalibrating an equation by updating the baseline risk does not change the original c-statistic. Explained variation is obtained from the re-estimated risk equations. For the Framingham 1991 CVD equation explained variation is estimated for all individuals, as women and men are modelled in one equation.

Abbreviations: ASCVD, atherosclerotic cardiovascular disease; BP, blood pressure; CVD, cardiovascular disease.

^a^Depending on the equation, the predictor cholesterol may include total and HDL cholesterol, and all interactions with these variables. For example, the PC equations include ln(total cholesterol), ln(HDL cholesterol), and the pair-wise interactions of the ln(age) with the ln(total cholesterol) and the ln(HDL cholesterol).

^b^Depending on the risk equation, the predictor systolic BP includes systolic BP or untreated/treated systolic BP. For example, the Framingham 2008 equation includes ln(treated systolic BP), and ln(untreated systolic BP).

### Updating enhanced the agreement of observed and predicted risk

**Figure 3:**
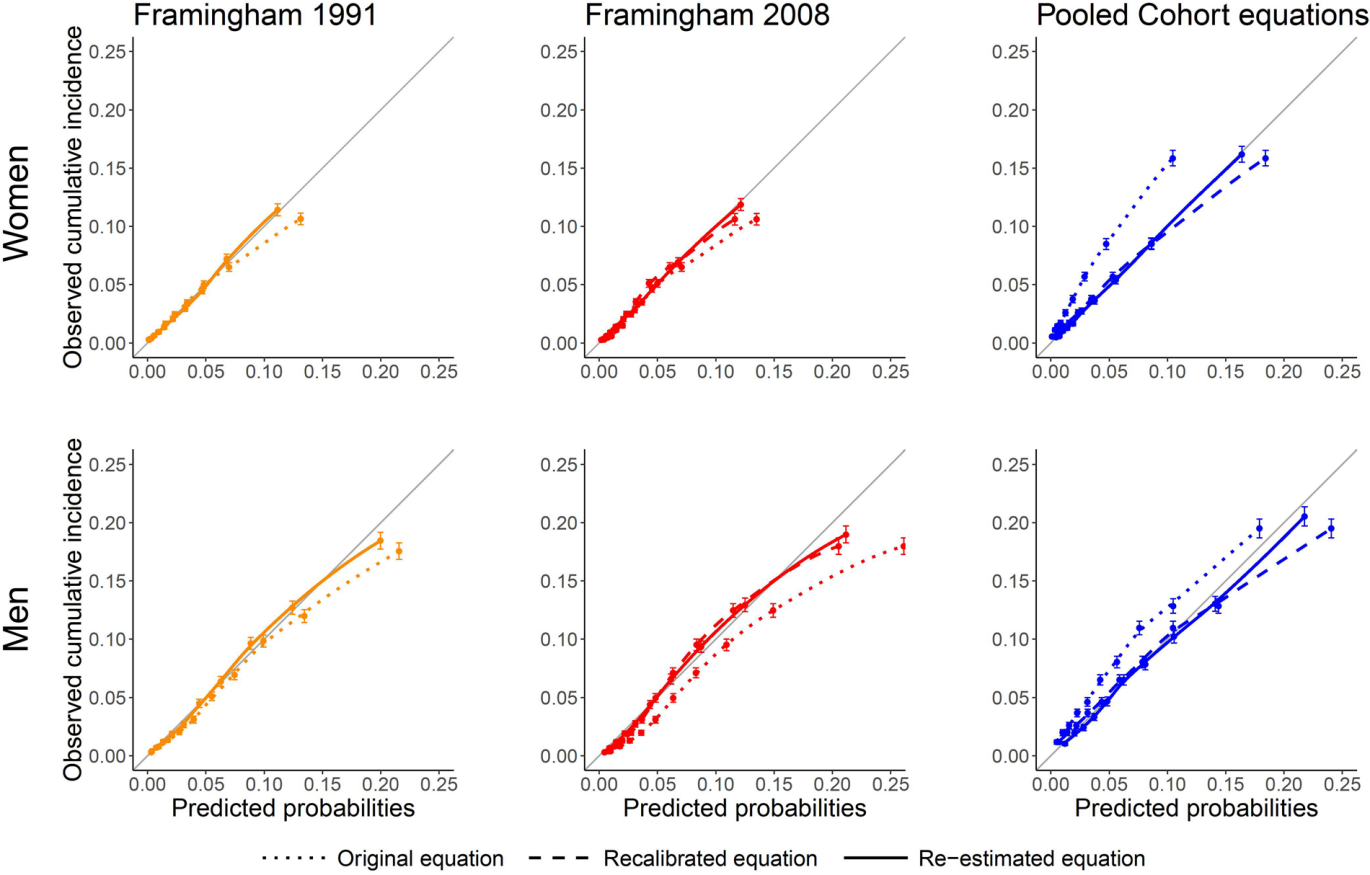
Calibration plots for the five-year CVD risk for women and men. Error bars represent 95%-confidence intervals. Dotted lines visualize predictions from the original equations, dashed lines show predictions from recalibrated equations and continuous lines give predictions from re-estimated equations. In a perfectly calibrated equation, the calibration curve follows the diagonal; a calibration curve above the diagonal indicates underprediction, whereas a calibration curve below the diagonal indicates overprediction. The parametric Framingham 1991 equation cannot be recalibrated. Abbreviations: ASCVD, atherosclerotic cardiovascular disease; CVD, cardiovascular disease.

Calibration plots for the five-year risk, as shown in Fig. 3, visualize the agreement between observed cumulative incidences and predicted probabilities. In the study cohort, the best calibration of the original equations was achieved by the FR1991 equation. Especially for individuals with higher predicted probabilities, re-estimation improved calibration for this equation. The original FR2008 equation overestimated the observed cumulative incidence of CVD especially for men. Updating improved its calibration as for the FR1991 equation. Applying the original PC equations led to considerable underestimation, also for individuals with low predicted probabilities. However, recalibration and especially re-estimation improved the equation reaching almost perfect calibration.

After updating all estimates of calibration-in-the-large were within 0.2 percentage points of the optimal value zero (Supplementary Table S5). Calibration slopes were already very good for the original Framingham equations reaching the optimal value of one. However, for the PC equations re-estimation improved the calibration slope.

**Figure 4:**
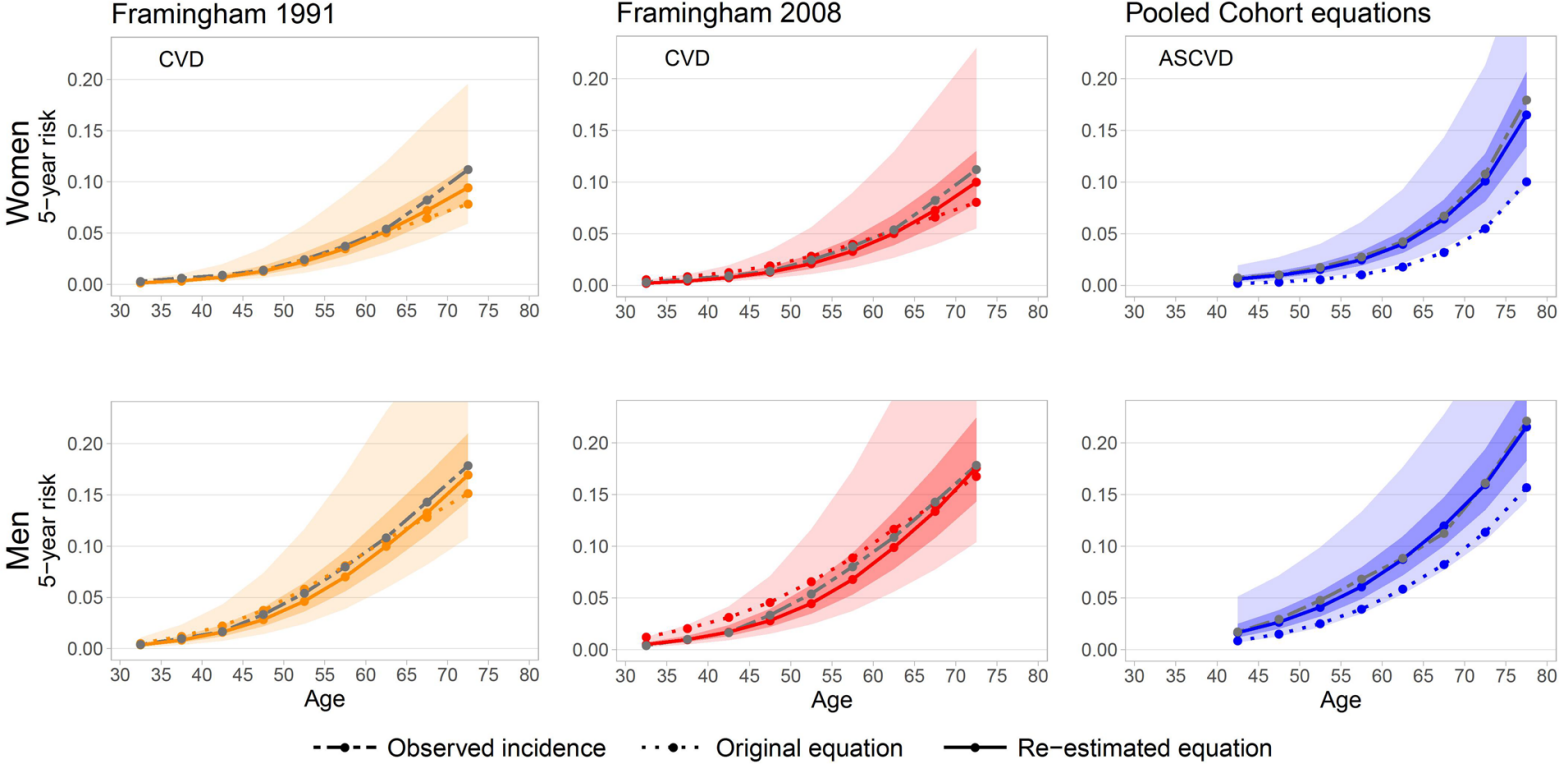
Median predicted five-year CVD risk for different age groups for women and men. Dashed, gray lines visualize the observed incidence, dotted lines show predictions from the original equations, and continuous lines give predictions from the re-estimated equations. The range from the 2.5th to the 97.5th and the 25th to the 75th percentile of the predicted five-year risk are visualized as shaded areas for the re-estimated equations. (The largest estimated 97.5^th^ percentile of the estimated five-year risk was 32.5% for women and 37.4% for men.).

Re-estimation improved the agreement of the predicted five-year (AS)CVD risk with the observed cumulative incidence rate across different ages (Fig. 4). For the original Framingham equations for individuals up to 65 years, the predicted risk corresponded to the observed incidence, but for older women the risk was underestimated. By re-estimating the equations, this underestimation was mostly removed. The original PC equations severely underestimated the observed five-year incidence for individuals of all ages, but this underestimation could be removed by re-estimation. We also assessed calibration for important subgroups in the original and the re-estimated equations (Supplementary Fig. S2). For individuals in high-risk groups, i.e., older individuals, individuals with diabetes or with hypertension, the original equations exhibited worse calibration (mainly overprediction for the two Framingham equations and underprediction for the PC equations) than individuals in the respective low-risk groups. However, the re-estimated equations were well calibrated for all subgroups, except for the re-estimated FR2008 equation where individuals with diabetes received too low predictions.

### Half of the individuals were reclassified in the re-estimated risk equations

In the re-estimated FR1991 equation (n = 525,498), 61.5% of the individuals remained in the risk category as classified with the original equation, 12.5% were reclassified to a higher risk category, and 26.0% were reclassified to a lower risk category (Table 3). In the re-estimated FR2008 equation (n = 532,668), 49.0% remained in the same category, 7.6% were up-classified, and 43.4% were down-classified. Absolute changes in predicted five-year risk due to the re-estimation were larger for FR2008 (1. to 99. percentile −10.83 to 4.29%), but were symmetric around zero for both FR equations (Supplementary Fig. 3). Men were more often reclassified than women (e.g., FR2008 men 58.0%, women 45.1%; Supplementary Table 7). These reclassified men were usually down-classified (e.g., FR2008 men 94.0%, women 74.7%). Reclassified individuals younger than 60 years were more often down-classified, while older reclassified individuals were more often up-classified. As indicated in Fig. 2, the regression coefficients of diabetes did not drastically change in the re-estimated Framingham equations. Consequently, reclassifications for individuals with and without diabetes were comparable, although individuals with diabetes were less often up-classified compared to individuals without diabetes. Individuals with hypertension were more often down-classified compared to individuals without hypertension (FR1991: 43.6% versus 16.8%, FR2008: 57.4% versus 36.0%).

**Table 3:** Risk reclassification tables.

			Re-estimated equation															
**Framingham 1991 general CVD**																		
			**<1.25%**		**1.25 – 2.49%**		**2.50 – 3.74%**		**3.75 - 4.99%**		**5.00 – 7.49%**		**7.50 - 9.99%**		**≥ 10.00%**		**Total**	
**Original equation**	**<1.25%**		149,963 (28.5)		10,905 (2.1)		279 (0.1)		10 (0.0)		0 (0.0)		0 (0.0)		0 (0.0)		161,157 (30.7)	
	**1.25 – 2.49%**		24,297 (4.6)		43,903 (8.4)		10,101 (1.9)		1,639 (0.3)		274 (0.1)		0 (0.0)		0 (0.0)		80,214 (15.3)	
	**2.50 – 3.74%**		2,354 (0.4)		21,582 (4.1)		20,591 (3.9)		8,259 (1.6)		3,649 (0.7)		31 (0.0)		0 (0.0)		56,466 (10.7)	
	**3.75 – 4.99%**		282 (0.1)		6,271 (1.2)		14,364 (2.7)		11,976 (2.3)		10,038 (1.9)		1,001 (0.2)		11 (0.0)		43,943 (8.4)	
	**5.00 – 7.49%**		30 (0.0)		1,902 (0.4)		9,197 (1.8)		14,796 (2.8)		24,873 (4.7)		10,944 (2.1)		1,074 (0.2)		62,816 (12.0)	
	**7.50 – 9.99%**		1 (0.0)		113 (0.0)		1,260 (0.2)		4,106 (0.8)		14,310 (2.7)		13,637 (2.6)		7,201 (1.4)		40,628 (7.7)	
	**≥10.00%**		0 (0.0)		9 (0.0)		160 (0.0)		797 (0.2)		6,930 (1.3)		14,039 (2.7)		58,339 (11.1)		80,274 (15.3)	
	**Total**		176,927 (33.7)		84,685 (16.1)		55,952 (10.6)		41,583 (7.9)		60,074 (11.4)		39,652 (7.5)		66,625 (12.7)		525,498 (100)	
**Framingham 2008 general CVD**																		
**Original equation**	**<1.25%**		104,862 (19.7)		1,921 (0.4)		34 (0.0)		1 (0.0)		1 (0.0)		0 (0.0)		0 (0.0)		106,819 (20.1)	
	**1.25 – 2.49%**		60,326 (11.3)		36,074 (6.8)		4,059 (0.8)		743 (0.1)		221 (0.0)		19 (0.0)		0 (0.0)		101,442 (19.0)	
	**2.50 – 3.74%**		7,863 (1.5)		37,049 (7.0)		16,900 (3.2)		5,026 (0.9)		2,585 (0.5)		274 (0.1)		24 (0.0)		69,721 (13.1)	
	**3.75 – 4.99%**		812 (0.2)		13,884 (2.6)		18,638 (3.5)		9,734 (1.8)		6,998 (1.3)		1,415 (0.3)		174 (0.0)		51,655 (9.7)	
	**5.00 – 7.49%**		99 (0.0)		4,632 (0.9)		16,019 (3.0)		17,749 (3.3)		21,158 (4.0)		8,086 (1.5)		2,236 (0.4)		69,979 (13.1)	
	**7.50 – 9.99%**		4 (0.0)		263 (0.0)		2,687 (0.5)		6,564 (1.2)		15,737 (3.0)		11,089 (2.1)		6,887 (1.3)		43,231 (8.1)	
	**≥10.00%**		0 (0.0)		33 (0.0)		390 (0.1)		1,827 (0.3)		10,427 (2.0)		16,216 (3.0)		60,928 (11.4)		89,821 (16.9)	
	**Total**		173,966 (32.7)		93,856 (17.6)		58,727 (11.0)		41,644 (7.8)		57,127 (10.7)		37,099 (7.0)		70,249 (13.2)		532,668 (100)	
**Pooled Cohort ASCVD Equations**																		
**Original equation**	**<1.25%**		84,567 (19.0)		74,867 (16.8)		11,528 (2.6)		505 (0.1)		34 (0.0)		0 (0.0)		0 (0.0)		171,501 (38.5)	
	**1.25 – 2.49%**		13 (0.0)		11,515 (2.6)		40,907 (9.2)		20,176 (4.5)		5,614 (1.3)		185 (0.0)		14 (0.0)		78,424 (17.6)	
	**2.50 – 3.74%**		0 (0.0)		168 (0.0)		4,258 (1.0)		16,734 (3.8)		22,951 (5.2)		3,353 (0.8)		305 (0.1)		47,769 (10.7)	
	**3.75 – 4.99%**		0 (0.0)		14 (0.0)		176 (0.0)		2,754 (0.6)		18,402 (4.1)		9,925 (2.2)		2,166 (0.5)		33,437 (7.5)	
	**5.00 – 7.49%**		0 (0.0)		3 (0.0)		18 (0.0)		329 (0.1)		8,408 (1.9)		19,161 (4.3)		16,134 (3.6)		44,053 (9.9)	
	**7.50 – 9.99%**		0 (0.0)		0 (0.0)		0 (0.0)		7 (0.0)		513 (0.1)		4,127 (0.9)		22,348 (5.0)		26,995 (6.1)	
	**≥10.00%**		0 (0.0)		0 (0.0)		0 (0.0)		6 (0.0)		26 (0.0)		592 (0.1)		42,382 (9.5)		43,006 (9.7)	
	**Total**		84,580 (19.0)		86,567 (19.4)		56,887 (12.8)		40,511 (9.1)		55,948 (12.6)		37,343 (8.4)		83,349 (18.7)		445,185 (100)	
**Legend: Observed 5-year risk (in %) for CVD and ASCVD**																		
	<1	1-2	2-3	3-4	4-5	5-6	6-7	7-8	8-9	9-10	10-11	11-12	12-13	13-14	14-15	15-16	16-17	17-18

Risk reclassification tables for the estimates 5-year risk (in %) for general cardiovascular disease (CVD) for the two Framingham equations and for atherosclerotic CVD (ASCVD) for the Pooled Cohort equations. Assuming constant hazard, approximately twice the estimated 5-year risk corresponds to the 10-year risk. For more details on the appropriateness of this assumption in this context and on the conversion, see Supplementary Figure 1. Individuals classified to cells in the diagonal (cells with a black frame) remain in the same risk category, irrespective if the original or the re-estimated equation is applied. All other individuals are re-classified to another risk category. Grey colors indicate the observed 5-year risk. The darker the grey color in a cell, the higher the observed 5-year risk of the individuals classified to this cell. (The observed 5-year risk was computed only for cells with at least 100 observations and at least one event.) If a re-estimated equation improves the discrimination of (AS-)CVD events, then separately for each row of Table 3, cells left of the diagonal should be colored in a lighter shade of grey compared to the cell in the diagonal, and cells right of the diagonal should be colored in a darker shade of grey compared to the cell in the diagonal. The observed 5-year risks and 95%-confidence intervals are reported in Supplementary Table 6. For a more precise view on the movement of participants between risk categories, we report reclassifications tables separate for women and men, individuals of different age groups, and for individuals with and without diabetes and hypertension in Supplementary Figure 3 and Table 7. Abbreviations: ASCVD, atherosclerotic cardiovascular disease; CVD, cardiovascular disease.

For the PC equations (n = 445,185), the results were different; 35.5% remained in their risk category, but 64.1% were up-classified and only 0.4% were down-classified. Absolute changes in predicted probabilities due to the re-estimation ranged from −1.05 to 9.58% (1. to 99. percentile). The distribution of reclassifications was similar for women and men. In the PC equations, age is modeled with a non-linear effect and pair-wise interactions with age are included (Fig. 2). This can explain the relation between reclassifications and age:

Individuals from 50 to 69 (n = 233,797) were up-classified in more than 80%. Among young individuals (40 to 49, n = 149,070) 39.9% were up-classified, while 51.9% of old individuals (70 to 79, n = 62,318) were up-classified.

As the two Framingham equations have identical definitions of the CVD outcome, the two re-estimated equations can be directly compared. Most individuals (83.1%, n = 436,579) remained in the same risk category, while 7.1% (n = 37,519) were up-classified in the re-estimated FR1991 equation (compared to FR2008), and 9.8% (n = 51,400) were down-classified. The percentage of reclassifications (no, up and down) was similar among women and men.

Risk equations are re-estimated to improve the discrimination of individuals with and without (AS-)CVD events. Hence, individuals who are down-classified after re-estimation should show lower observed 5-year risks compared to the classification from the original equation, whereas individuals who are up-classified should have higher observed 5-year (AS-)CVD risks (see different shadings in Table 3 and Supplementary Table 6). For example, the original FR2008 equation classified 101,442 individuals into the 5-year CVD risk category ‘1.25 to 2.49%’ (Table 3). Of those, 36,074 individuals (35.6%) were classified into the same category with the re-estimated FR2008 equation. These individuals had an observed 5-year CVD risk of 1.66% (1.49; 1.84), while for the 60,326 (59.5%) individuals who were down-classified a 5-year CVD risk of 1.04% (0.75; 1.33) was observed. Further 4,059 (4.0%) individuals were up-classified by one risk category and had an observed 5-year CVD risk of 2.97% (2.32; 3.61), 743 (0.7%) were up-classified by two risk categories and had an observed 5-year CVD risk of 5.24% (3.43, 7.02), and 221 (0.2%) were up-classified by three risk categories and had an observed 5-year CVD risk of 11.97% (6.84, 16.82). Improved discrimination was observed for all three evaluated equations.

### Age was the most important predictor for CVD

In the re-estimated equations, predictors explained on average 7.37% of the variation of general CVD, whereas the PC equations explained on average 5.81% of the variation of ASCVD (Table 2). Assessing the importance of groups of predictors (e.g. all age-related model terms) by evaluating the drop in explained variation caused by removing such a group, the FR2008 and the PC equations had similar rankings of the importance of groups of predictors. The ranking of risk factor importance was different for the FR1991 equation where women and men were not separately modelled. In all equations, predictors relating to age were identified as the most important ones.

## Discussion

We had previously shown that both Framingham general CVD equations slightly overestimated the average five-year risk, while the PC equations for ASCVD underestimated it in the general Austrian population^12^. As the predictive performance of these equations was not optimal and as such risk equations are currently used during standardized health-screenings in Austria, we evaluated to which extent risk prediction could be improved by updating the equations. Risk equations tailored to local and contemporary circumstances can improve the quality of predictions for individuals attending health-screenings in the future and can improve the decision making of healthcare professionals regarding lifestyle counseling and possible treatment initiation.

As expected, updating did not lead to large improvements in discriminative ability measured by c-statistics, as discrimination only assesses the order of the predicted risk in the study cohort. However, updating led to improved agreement of observed and predicted risk. Focusing on individuals in high-risk groups, e.g., the elderly, individuals with diabetes or with hypertension, the updated equations also performed better. The calibration of the three updated equations was superior to that of the original equations. Especially for the PC equations, re-estimated regression coefficients of some CVD predictors were considerably different from the original regression coefficients. This may be due to case-mix differences between the development cohorts, which mostly consisted of individuals who lived in the U.S. in the late 1980s, and the contemporary European study cohort. Additionally, changes over time with regard to the awareness of CVD risk, changes in lifestyle, or changes in utilization of lipid-lowering and antihypertensive medication, might be responsible for the observed differences. Finally, random variability, in particular in the smaller development cohorts, may be a cause of the discrepancies.

The evaluated equations only included traditional CVD risk factors that could not optimally predict the occurrence of CVD as the explained variation was 7.37% for general CVD and 5.81% for ASCVD in the re-estimated equations. The non-modifiable risk factor age was the most important predictor in all equations, explaining individually 5.01% of the outcome variation. Age can be interpreted as a proxy of exposure time to known risk factors. However, age is associated with many risk factors of overall health, which are not included in the equations. As such the high explained variation of age might also be interpreted as a proxy for unmeasured risk factors for CVD.

### More updating studies are required

External validation studies evaluating the transportability of a risk equation to a new population are still not common; e.g., in a recent systematic review, Ban *et al*. found that only 23% of 125 CVD equations were externally validated^32^. Updating studies combining evidence from published equations with new data to tailor the prediction tool to other populations or to update them because of changes over time are even rarer. In the field of cardiology, although many risk equations for CVD in the general population were developed, the advantages of updating are not utilized enough^6,8,11,33,34^. Updating does not require new selection of predictors, their functional forms (e.g., transformations) or inclusion of possible pair-wise interactions, and thus induces less uncertainty than developing a new equation from scratch.

### Opportunities and limitations of this study

This study was conducted with a large, contemporary cohort comprising almost a third of the Austrian population aged 30 to 79 years. All selected 1.7 M individuals met the inclusion criteria of the investigated equations and no further inclusion/exclusion criteria were applied. The random split of the data into training and test set did not have any impact on the results as the sample size was sufficiently large. Since the data were collected electronically as part of a standardized routine health-screening program, CVD risk factors were assessed in a homogenous way with almost no missing values. We only considered risk equations for the combined outcome of non-fatal and fatal CVD, because both have severe consequences and are of great relevance for individuals attending health screenings and for society. Morbidity and disability due to CVD are causes of major economic burden to societies and their health care systems^35^.

Our study had some limitations. We investigated the five-year risk because the data from the Austrian health-screening was available from 2009 to 2015 only. Risk equations for this time horizon are also currently applied in the standardized Austrian health-screenings. Assuming a constant hazard, the estimated ten-year risk can be extrapolated from the predicted five-year risk. The ten-year risk is roughly double the five-year risk (for five-year risk up to 10%). The extrapolation relies on constant hazard assumption, which seems plausible given that the observational period did not start with an intervention. A plot of the hazard for the (AS-)CVD outcome also substantiates this. For the FR2008 equation, only the information required to estimate the ten-year risk was reported. Therefore, we assumed a constant hazard over time to estimate the five-year baseline survival. Due to data availability of hospitalizations, we could not exclude individuals with CVD history prior to 2008. Misclassification of fatal events may have been introduced by the probabilistic assignment of ambiguous causes of deaths. Nonetheless, this potentially affected only 10.3% of the combined CVD outcomes. However, for this reason we did not re-estimate the SCORE equation, which focuses only on fatal CVD. In the context of preventive health-screenings in the general population, estimation of fatal and non-fatal CVD risk also seems more relevant for participants and their physicians. Although the health-screening program is free-of-charge and is offered by most general practioners, participation is voluntary and implies self-selection. Therefore, individuals in our study cohort may have had greater health awareness than the general population. Indeed, on average they were slightly younger and had a marginally better overall survival rate than the general Austrian population.

In general, the predictive performance of the equations could still be improved by considering additional non-linearities of or higher-order interactions among risk factors. Alternatively, one could leave the path of classical statistical modelling and apply machine learning methods (e.g. random forests or deep learning) to improve the accuracy of prediction. Previously, the application of such modern prediction methods was infeasible because of limited sample sizes. In our study, we aimed at making use of the existing knowledge from published equations as much as possible and focused on minimal changes resulting from recalibration and re-estimation approaches.

## Conclusions

CVD risk equations are developed to inform individuals about their CVD risk in a certain time frame and to support healthcare professionals in their decision making with regard to advocate lifestyle changes and possibly initiating treatment. To optimize preventive care, we recommend our re-estimated equations based on a contemporary cohort of one third of all Austrian inhabitants for the Austrian and similar general populations. The five-year general CVD risk based on the re-estimated Framingham 2008 equation and the five-year ASCVD risk based on the re-estimated Pooled Cohort equation can conveniently be calculated using an online risk calculator available at https://cemsiis.meduniwien.ac.at/en/kb/science-research/software/clinical-software/cvdrisk/.

## Supplementary information

Supplementary information.

## Data Availability

The Main Association of the Austrian Social Security Institutions provided the data supporting the findings of this study. Within a collaboration with the Main Association of the Austrian Social Security Institutions access to the data is possible. Contact the corresponding author for more information.
