# Publisher Correction: Re-estimation improved the performance of two Framingham cardiovascular risk equations and the Pooled Cohort equations: A nationwide registry analysis

**DOI:** 10.1038/s41598-020-67920-8

**Published:** 2020-06-26

**Authors:** Christine Wallisch, Georg Heinze, Christoph Rinner, Gerald Mundigler, Wolfgang C. Winkelmayer, Daniela Dunkler

**Affiliations:** 1 Medical University of Vienna, CEMSIIS, Section for Clinical Biometrics, Vienna, Austria; 2 Medical University of Vienna, CEMSIIS, Section for Medical Information Management, Vienna, Austria; 3 Medical University of Vienna, Department of Medicine 2, Division of Cardiology, Vienna, Austria; 4 Baylor College of Medicine, Section of Nephrology, Houston, TX USA

Correction to: *Scientific Reports* https://doi.org/10.1038/s41598-020-64629-6, published online 18 May 2020

This Article contains errors in Table 3. In the HTML and PDF versions of this Article, the grey colouration indicating the observed 5-year risk (in %) for CVD and ASCVD is incorrect. The correct Table 3 appears below as Table 1.

**Table 1:**
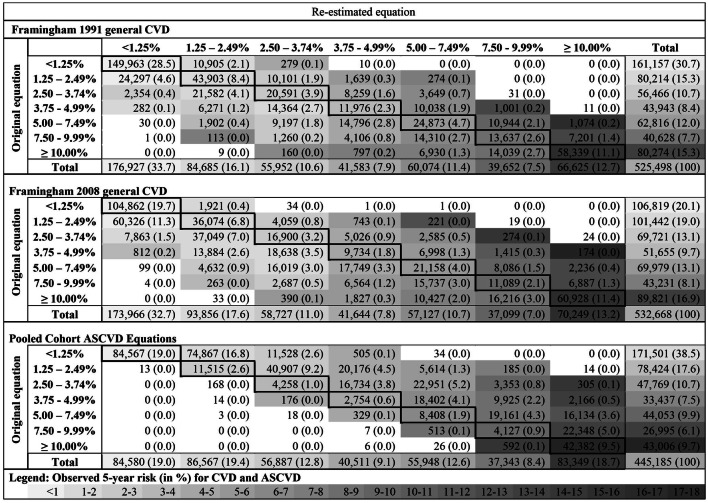
Risk reclassification tables. Risk reclassification tables for the estimates 5-year risk (in %) for general cardiovascular disease (CVD) for the two Framingham equations and for atherosclerotic CVD (ASCVD) for the Pooled Cohort equations. Assuming constant hazard, approximately twice the estimated 5-year risk corresponds to the 10-year risk. For more details on the appropriateness of this assumption in this context and on the conversion, see Supplementary Figure 1. Individuals classified to cells in the diagonal (cells with a black frame) remain in the same risk category, irrespective if the original or the re-estimated equation is applied. All other individuals are re-classified to another risk category. Grey colors indicate the observed 5-year risk. The darker the grey color in a cell, the higher the observed 5-year risk of the individuals classified to this cell. (The observed 5-year risk was computed only for cells with at least 100 observations and at least one event.) If a reestimated equation improves the discrimination of (AS-)CVD events, then separately for each row of Table 3, cells left of the diagonal should be colored in a lighter shade of grey compared to the cell in the diagonal, and cells right of the diagonal should be colored in a darker shade of grey compared to the cell in the diagonal. The observed 5-year risks and 95%-confidence intervals are reported in Supplementary Table 6. For a more precise view on the movement of participants between risk categories, we report reclassifications tables separate for women and men, individuals of different age groups, and for individuals with and without diabetes and hypertension in Supplementary Figure 3 and Table 7. Abbreviations: ASCVD, atherosclerotic cardiovascular disease; CVD, cardiovascular disease.

