# Supplementary material for: Re-estimation improved the performance of two Framingham cardiovascular risk equations and the Pooled Cohort equations: A nationwide registry analysis
Source: Sci Rep. 2020 May 18;10:8140. doi: 10.1038/s41598-020-64629-6 (PMC7235230; doi:10.1038/s41598-020-64629-6)
Supplement: Supplementary file 1 — Supplementary information. [file 41598_2020_64629_MOESM1_ESM.pdf]

Supplementary Material

Wallisch C, Heinze G, Rinner C, Mundigler G, Winkelmayer W.C., and Dunkler D. Re-estimation improved the performance of two Framingham cardiovascular risk equations and the Pooled Cohort equations: A nationwide registry analysis, 2020

Table of contents

Supplementary Methods S1. Data preparation 2
Supplementary Table S2a. The Framingham 1991 general CVD equation 3
Supplementary Table S2b. The Framingham 2008 general CVD equation 5
Supplementary Table S2c. The Pooled Cohort equations 7
Supplementary Figure 1. Compute the ten-year risk from the five-year risk 10
Supplementary Table S3. Baseline characteristics of individuals in the training and test set 11
Supplementary Table S4. The Austrian study cohort versus the general Austrian population 12
Supplementary Table S5. Calibration in-the-large and calibration slope for the original, recalibrated and re-estimated risk equations. 13
Supplementary Figure S2: Calibration plots for subgroups 14
Supplementary Figure S3: Differences in predicted five-year risk between re-estimated and original equations 16
Supplementary Table S6: Observed five-year risk of cardiovascular disease (CVD) and atherosclerotic cardiovascular disease (ASCVD) 18
Supplementary Table S7: Risk reclassification tables for subgroups 20
Supplementary references 53

Supplementary Methods S1. Data preparation

Distributions of blood pressure (BP) and blood parameter measurements were truncated at the respective 0.5th and 99.5th percentiles. Missing values in BP treatment (8.4%) were assumed to indicate no treatment. The low number of missing values in risk factors (0.08% of individuals) allowed a complete-case analysis.
Causes of death given in ICD-10 codes (International Classification of Diseases and Related Health Problems, 10th edition) were provided by the Austrian's federal institute for Statistics. Causes of death were split into CVD-related and CVD-unrelated death. The category definitions varied slightly for each equation because of different CVD definitions. For the assignment we used the date of death, gender, and birth year. For most deceased individuals in the health screening data base unambiguous assignment of CVD death was possible. The remaining deceased individuals, received a probability that his/her death was CVD-related. For an individual, this probability was the number of CVD-related deaths of his/her possible matchings divided by the total number of his/her possible matchings in the registry of deaths. Using these calculated probabilities, CVD-related death was randomly assigned to each deceased individual. Misclassification of CVD deaths may have been introduced by the probabilistic assignment of ambiguous causes of deaths. This potentially affected only 10.3% of the combined (fatal and non-fatal) CVD outcomes.
Individuals with a history of CVD were excluded. These events were identified by hospital stays (with information on the discharge diagnoses in ICD-10 codes) prior to the health screening. For discharge diagnoses using ICD-9 codes, we applied forward mapping to transform ICD-9 codes to ICD-10 codes. The exact exclusion criteria can be found in Supplementary Tables 2a-c. As the investigated equations had different exclusion criteria, different subsets of the final data set were used in the analysis. The following Venn diagram shows the overlap of individuals in the subsets for each equation.

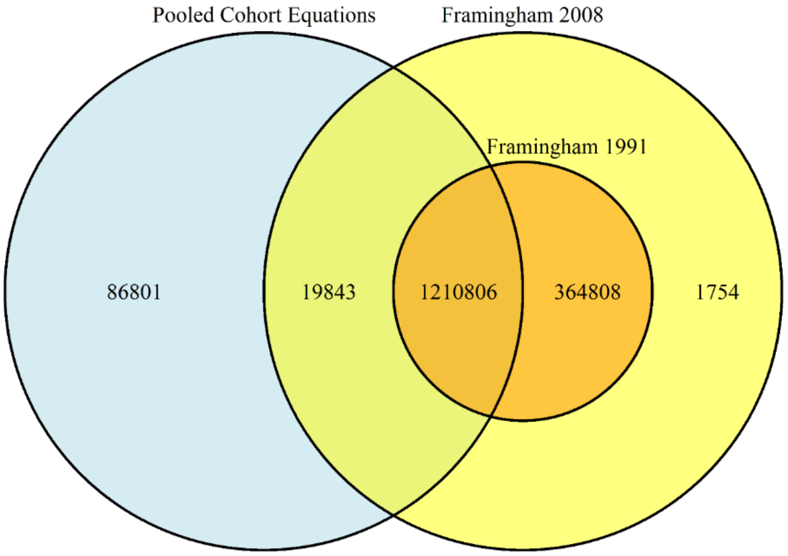

R 3.5.0 and SAS 9.4 were used for analysis and figure preparation.[1 2]

Supplementary Table S2a. The Framingham 1991 general CVD equation

Baseline characteristics

Baseline characteristics of the test set of the Austrian study cohort are compared to the characteristics of the derivation cohort of the Framingham 1991 equation.[3,4] The baseline characteristics in the training set and the test set of the Austrian study cohort are almost identical.
Individuals aged 30 to 74, and free of documented CVD and cancer (except basal cell carcinoma) were included. Values are median, 5% and 95% quantile, or *n* and percentage.

	Women		Men	
**Baseline Characteristics**	**Austrian test set (2009-2014)** *	**Framingham population (1968-1975)** *	**Austrian test set (2009-2014)** *	**Framingham population (1968-1975)** *
N	282,732	2,983	242,769	2,590
Age	49 (31, 71)	NA	49 (31, 70)	NA
Total cholesterol mmol	5.41 (3.86, 7.38)	5.49 (3.91, 7.64)	5.39 (3.76, 7.33)	5.44 (3.96, 7.33)
HDL cholesterol mmol	1.61 (1.01, 2.49)	1.45 (0.91, 2.23)	1.27 (0.80, 2.02)	1.11 (0.73, 1.74)
Systolic BP mmHg	125 (100, 161)	123 (100, 168)	130 (110, 165)	128 (109, 168)
Smoking	59,282 (20.97%)	1,159 (39%)	60,648 (24.98%)	1,055 (41%)
Diabetes	11,141 (3.94%)	154 (5%)	13,897 (5.72%)	183 (7%)
Median linear predictor †	4.32	4.19	3.91	3.85

* Time of recruitment is given in brackets.
† calculated for each group of age and weighted by group size
Abbreviations: BP, blood pressure; HDL, high-density lipoprotein; NA, not available; SD, standard deviation.

Exclusion criteria

In the Framingham 1991 equation, individuals were excluded if they were not aged 30 to 74 or experienced a cardiovascular event before their first health screening. Anderson et al. (1991) defined cardiovascular disease (CVD) as coronary heart disease (CHD), stroke, transient ischemia, congestive heart failure, and peripheral vascular disease.[4] We identified ICD-10 codes in the table below for this definition. Additionally, the authors excluded individuals with cancer other than basal cell carcinoma. Therefore, we excluded patients having ICD-10 codes C00-C97 (malignant neoplasms) without C44.3, D00-D09 (in situ neoplasms), or the corresponding ICD-9 codes. Finally, the Austrian study cohort for the Framingham 1991 equation comprised 1 575 614 individuals.

Code	Description
I11.0	Hypertensive heart disease with (congestive) heart failure
I13.0, I13.2	Hypertensive heart and renal disease with (congestive) heart failure
I20	Angina pectoris
I21	Acute myocardial infarction
I25	Chronic ischaemic heart disease (excl. I25.3,.4,.8)
I50	Heart failure
I60	Subarachnoid haemorrhage (excl. I60.8)
I61	Intracerebral haemorrhage
I62	Other nontraumatic intracranial haemorrhage
I63	Cerebral infarction (excl. I63.6)
I64	Stroke, not specified as haemorrhage or infarction
I65	Occlusion and stenosis of precerebral arteries, not resulting in cerebral infarction
I66	Occlusion and stenosis of cerebral arteries, not resulting in cerebral infarction
I67	Other cerebrovascular diseases (excl. I67.5,.6)
I70.2	Atherosclerosis of arteries of extremities
I73.9	Peripheral vascular disease, unspecified (Intermittent claudication)
G45	Transient cerebral ischaemic attacks and related syndromes

Definition of CVD

Anderson et al. defined CVD as myocardial infarction, death from coronary heart disease, angina pectoris, coronary insufficiency, stroke incl. transient ischemia, congestive heart failure and peripheral vascular disease.[4] This definition in ICD-10 codes was already described in the table above.

Regression coefficients & model formula

Predictors		Original equation	Re-estimated equation
Theta0	θ_0	0.6536	0.1793
Theta1	θ_1	-0.2402	-0.0556
Intercept	β_0	18.8144	24.6858
Female (Yes=1)	β_1	-1.2146	-0.2933
ln(age)	β_2	-1.8443	-3.7514
ln(age)*female	β_3	0.3668	0.1850
ln(Systolic BP)	β_4	-1.4032	-0.9237
Smoking (Yes =1)	β_5	-0.3899	-0.4785
ln(total / HDL cholesterol)	β_6	-0.5390	-0.4625
Diabetes (Yes =1)	β_7	-0.3036	-0.5031
Diabetes*female	β_8	-0.1697	0.0285

$$S(5) = -\exp(-\exp\left(\frac{\ln(5) - \mu}{\sigma}\right))$$

$$\mu = \beta_0 + \beta_1 * female + \beta_2 * \ln(age) + \beta_3 * \ln(\text{age}) * female + \beta_4 * \ln(systolic\ BP) + \beta_5 * smoking + \beta_6 * \ln\left(\frac{total\ chol}{HDL\ chol}\right) + \beta_7 * diabetes + \beta_8 * diabetes * female$$

$$\sigma = \exp(\theta_0 + \theta_1 * \mu)$$

R code to calculate the five-year risk

```
risk_fr91 <- function(age, sex, SBP, totchol, hdlchol, smoke, diabetes, time=5){

  # age: in years
  # sex: 0 = male, 1 = female
  # SBP: systolic blood pressure in mmHg
  # totchol: total cholesterol in mg/dl
  # HDL chol: HDL cholesterol in mg/dl
  # smoke: 0 = non-smoker, 1 = smoker
  # diabetes: 0 = no diabetes, 1 = diabetes
  # time: prediction time horizon in years

  est <- c(0.1793, -0.0556, 24.5658, -0.2933, -3.7514, 0.1850, -0.9237,
           -0.4785, -0.4625, -0.5031, 0.0285)


  mu <- est[3] + c(sex, log(age), log(age)*sex, log(SBP), smoke, log(totchol/hdlchol),
                   diabetes, diabetes*sex) %*% est[4:11]
  sigma <- exp(est[1] + mu*est[2])
  u <- (log(time) - mu)/sigma
  risk <- 1- exp(-exp(u))
  return(risk)
}
```

Supplementary Table S2b. The Framingham 2008 general CVD equation

Baseline characteristics

Baseline characteristics of the test set of the Austrian study cohort are compared to the characteristics of the derivation cohorts of the CVD risk equations.[5]
The Framingham 2008 equation was developed for individuals from 30 to 74 years free of documented CVD. Values are mean and standard deviation (SD), or *n* and percentage.

Baseline characteristics	Women		Men	
	**Austrian test set (2009-2014)**	**Framingham cohort (1968-1987)**	**Austrian test set (2009-2014)**	**Framingham cohort (1968-1987)**
N	286,729	4,522	245,939	3,969
Age	50.1 (12.2)	49.1 (11.1)	50.0 (11.9)	48.5 (10.8)
Total cholesterol mmol	5.49 (1.08)	5.57 (1.14)	5.44 (1.08)	5.50 (1.02)
HDL cholesterol mmol	1.66 (0.45)	1.49 (0.40)	1.33 (0.38)	1.16 (0.32)
Systolic BP mmHg	127.3 (18.7)	125.8 (20.0)	133.0 (17.3)	129.7 (17.6)
BP treatment	35,951 (12.54%)	532 (11.76%)	34,061 (13.85%)	402 (10.13%)
Smoking	59,977 (20.92%)	1,548 (34.23%)	61,158 (24.87%)	1,398 (35.22%)
Diabetes	11,401 (3.98%)	170 (3.76%)	14,225 (5.78%)	258 (6.50%)
Mean linear predictor	26.11	26.19	23.91	23.98

Abbreviations: BP, blood pressure; HDL, high-density lipoprotein; SD, standard deviation.

Exclusion criteria

For the Framingham 2008 equation, individuals were included if they were between 30 and 74 years and free of documented CVD. CVD was defined as in the Framingham 1991 equation. The Austrian study cohort for the Framingham 2008 equation comprised 1 597 211 individuals.

Outcome definition

D'Agostino et al. defined the outcome CVD as a composite of coronary heart disease (coronary death, myocardial infarction, coronary insufficiency, and angina), cerebrovascular events (including ischemic stroke, hemorrhagic stroke, and transient ischemic attack), peripheral artery disease (intermittent claudication) and heart failure.[5] We identified the same ICD-10 codes as used in Framingham1991 equation for this definition.

Regression coefficients & model formula

Predictors	Women		Men	
	**Original equation**	**Re-estimated equation**	**Original equation**	**Re-estimated equation**
ln(age)	2.32888	4.5998	3.06117	4.4064
ln(total cholesterol)	1.20904	0.1235	1.12370	0.3567
ln(HDL chol)	-0.70833	-0.7536	-0.93263	-0.5852
ln(systolic BP) if untreated	2.76157	0.9620	1.93303	0.9439
ln(systolic BP) if treated	2.82263	1.0217	1.99881	1.0044
Smoking (Yes=1)	0.52873	0.5780	0.65451	0.5805
Diabetes (Yes=1)	0.69154	0.4214	0.57367	0.4700
Mean LP	26.1931	20.2478	23.9802	21.5433
$S_0(5)^*$	0.9747	0.9840	0.9431	0.9658
$S_0(10)$	0.9501		0.8894	

* approximated by assuming a constant hazard over time for the original equation
Abbreviations: LP, linear predictor.

$$S(t) = S_0(t)^{-\exp(X\beta - mean\ LP)}$$

$$X\beta = \beta_1 * \ln(age) + \beta_2 * \ln(total\ chol) + \beta_3 * \ln\left(\frac{total\ chol}{HDL\ chol}\right) + \beta_4 * systolic\ BP * 1_{intake\ of\ BP\ medication} + \beta_5 * systolic\ BP * 1_{no\ intake\ of\ BP\ medication} + \beta_6 * smoking + \beta_7 * diabetes$$

R code to calculate the five-year risk

```
risk_fr08 <- function(age, sex, SBP, SBP_med, totchol, hdlchol, smoke, diabetes){

  # age: in years
  # sex: 0 = male, 1 = female
  # SBP: systolic blood pressure in mmHg
  # SBP_med: 0 = untreated BP, 1 = treated BP
  # totchol: total cholesterol in mg/dl
  # hdlchol: HDL cholesterol in mg/dl
  # smoke: 0 = non-smoker, 1 = smoker
  # diabetes: 0 = no diabetes, 1 = diabetes


  # for sex=0
  S5_m<-  0.9658
  estimates_m <- c(4.4064, 0.3567, -0.5852, 0.9439, 1.0044, 0.5805, 0.4700)
  lp_m <- 21.5433

  # for sex=1
  S5_w<- 0.9840
  estimates_w <- c(4.5998, 0.1235, -0.7536, 0.9620, 1.0217, 0.5780, 0.4214)
  lp_w<- 20.2478


  X <- c(log(age), log(totchol), log(hdlchol), log(SBP)*(SBP_med==0),
         log(SBP)*(SBP_med==1), smoke, diabetes)

  Xbeta<- NULL
  risk<-NULL
  if(sex==0){
    Xbeta <- X %*% estimates_m - lp_m
    risk  <- 1 - S5_m^(exp(Xbeta))
  }
  if(sex==1){
    Xbeta <- X %*% estimates_w - lp_w
    risk <- 1 - S5_w^(exp(Xbeta))
  }
  return(risk)
}
```

Supplementary Table S2c. The Pooled Cohort equations

Baseline characteristics

Baseline characteristics of the test set of the Austrian study cohort are compared to the characteristics of the derivation cohorts of the Pooled Cohort equations.[6]
The Pooled Cohort equations were derived using the following U.S.-cohort studies

- Atherosclerosis Risk in Communities (ARIC) study (1987-1989)
- Cardiovascular Health Study (CHS) (1989)
- Coronary Artery Risk Development in Young Adults (CARDIA) study (1984), including individuals aged 40 or older who attended the ten-year examinations (1995/1996)
- Framingham Original and Offspring cohort data (1948, 1971)

Sex- and race-specific models were developed.[5] Here, baseline characteristics of individuals used to develop the equation for non-Hispanic whites are compared to the test set of the Austrian study cohort.

Women

Baseline Characteristics	Austrian test set (2009-2014)	ARIC (1987-1989)	CARDIA (1995-1996)	CHS (1989)	Framingham (1948, 1971)
N	237,872	5,508	131	2,131	3,470
Age, *range*	40-79	44-65	40-42	65-79	40-74
Age, *mean (SD)*	56.2 (10.7)	53.9 (5.7)	40.1 (0.3)	70.8 (3.8)	53.5 (8.7)
Total cholesterol mmol	5.66 (1.07)	5.65 (1.09)	4.96 (0.75)	5.78 (0.98)	5.81 (1.12)
HDL cholesterol mmol	1.66 (0.45)	1.50 (0.44)	1.40 (0.33)	1.54 (0.41)	1.50 (0.41)
Untreated systolic BP mmHg	129.0 (18.6)	114.3 (16.4)	104.5 (10.5)	130.4 (20.0)	126.7 (18.8)
Treated systolic BP mmHg	141.6 (18.2)	129.1 (18.0)	108.0 (4.4)	140.8 (19.9)	147.9 (19.7)
BP treatment	17.8%	16.7%	2.3%	32.9%	13.2%
Smoking	18.8%	24.5%	17.6%	13.7%	32.8%
Diabetes	5.6%	6.1%	1.5%	9.9%	4.7%
Mean linear predictor	-29.26	-29.18			

Values are mean and standard deviation (SD) or *n* (%).
Abbreviations: BP, blood pressure; HDL, high density lipoprotein; SD, standard deviation.

Men

Baseline Characteristics	Austrian test set (2009-2014)	ARIC (1987-1989)	CARDIA (1995-1996)	CHS (1989)	Framingham (1948, 1971)
N	207,313	4,692	131	2,131	3,470
Age, *range*	40-79	44-65	40-42	65-79	40-74
Age, *mean (SD)*	55.7 (10.4)	54.5 (5.7)	40.2 (0.4)	71.2 (3.8)	52.8 (8.5)
Total cholesterol mmol	5.49 (1.09)	5.45 (0.99)	4.82 (0.87)	5.19 (0.90)	5.61 (1.00)
HDL cholesterol mmol	1.34 (0.39)	1.12 (0.32)	1.11 (0.29)	1.23 (0.32)	1.17 (0.32)
Untreated systolic BP mmHg	133.7 (17.5)	118.3 (15.0)	112.5 (13.2)	131.5 (19.1)	129.9 (17.4)
Treated systolic BP mmHg	141.6 (17.6)	128.6 (16.7)	114.0 (11.3)	142.0 (22.4)	145.8 (19.9)
BP treatment	18.9%	16.6%	1.9%	30.4%	11.9%
Smoking	22.1%	24.5%	23.3%	10.7%	33.6%
Diabetes	7.9%	7.8%	2.9%	15.4%	7.7%
Mean linear predictor	61.07	61.18			

Exclusion criteria

For the Pooled Cohort equations,[6] the authors included individuals aged 40 to 79 and excluded individuals with a history of non-fatal recognized or unrecognized myocardial infarction, stroke, heart failure, percutaneous coronary intervention, coronary artery bypass surgery, or atrial fibrillation. We translated this definition to ICD-10 codes as shown in the table below and excluded individuals with a hospital stay due to one of those ICD-10 (or mapped ICD-9) codes. In the Austrian study cohort 1 337 475 individuals fulfilled these criteria.

Code	Description
I11.0	Hypertensive heart disease with (congestive) heart failure
I13.0,.2	Hypertensive heart and renal disease with (congestive) heart failure
I21	Acute myocardial infarction
I22	Subsequent myocardial infarction
I25.2	Old myocardial infarction
I48	Atrial fibrillation and flutter
I50	Heart failure
I60	Subarachnoid haemorrhage (excl. I60.8)
I61	Intracerebral haemorrhage
I62	Other nontraumatic intracranial haemorrhage
I63	Cerebral infarction (excl. I63.6)
I64	Stroke, not specified as haemorrhage or infarction
Z95.1	Presence of aortocoronary bypass graft
Z95.5	Presence of coronary angioplasty implant and graft

Outcome definition

Goff et al. defined their outcome as the occurrence of atherosclerotic cardiovascular disease (ASCVD),[6] which corresponds to nonfatal myocardial infarction or coronary heart disease death, or fatal or nonfatal stroke. We selected the following ICD-10 codes for these diseases:

Code	Description
I21	Acute myocardial infarction
I60	Subarachnoid haemorrhage (excl. I60.8)
I61	Intracerebral haemorrhage
I62	Other nontraumatic intracranial haemorrhage
I63	Cerebral infarction (excl. I63.6)
I64	Stroke, not specified as haemorrhage or infarction

Regression coefficients & model formula

Predictors	Women		Men	
	Original equation	Re-estimated equation	Original equation	Re-estimated equation
ln(age)	-29.799	-17.5033	12.334	11.1931
ln(age)^2	4.884	3.1733		
ln(total cholesterol)	13.540	5.6155	11.853	8.9746
ln(HDL chol)	-13.578	-5.3616	-7.990	-5.4405
ln(systolic BP) if treated	2.019	0.9048	1.797	0.8813
ln(systolic BP) if untreated	1.957	0.8530	1.764	0.8302
Smoking (Yes=1)	7.574	3.4245	7.837	4.3491
Diabetes (Yes=1)	0.661	0.3902	0.658	0.3526
ln(age):ln(total chol)	-3.114	-1.4054	-2.664	-2.1610
ln(age):ln(HDL chol)	3.149	1.1326	1.769	1.2075
ln(age):smoking	-1.665	-0.7380	-1.795	-0.9637
Mean LP	-29.67	-18.2223	60.69	48.4377
$S_0(5)$	0.9890	0.9759	0.9625	0.9474
$S_0(10)$	0.9665		0.9144	

Abbreviations: BP, blood pressure; chol, cholesterol; HDL, high density lipoprotein; LP, linear predictor.

$$S(t) = S_0(t)^{-\exp(X\beta - mean\ LP)}$$

$$X\beta = \beta_1 * \ln(age) + \beta_2 * \ln(age)^2 * 1_{female} + \beta_3 * \ln(total\ chol) + \beta_4 * \ln(HDL\ chol) + \beta_5 * \ln(systolic\ BP) * 1_{intake\ of\ BP\ medication} + \beta_6 * \ln(systolic\ BP) * 1_{no\ intake\ of\ BP\ medication} + \beta_7 * smoking + \beta_8 * diabetes + \beta_9 * \ln(age) * \ln(total\ chol) + \beta_{10} * \ln(age) * \ln(HDL\ chol) + \beta_{11} * \ln(age) * smoking$$

R code to calculate the five-year risk

```
risk_pce <- function(age, sex, SBP, SBP_med, totchol, hdlchol, smoke, diabetes){

  # age: in years
  # sex: 0 = male, 1 = female
  # SBP: systolic blood pressure in mmHg
  # SBP_med: 0 = untreated BP, 1 = treated BP
  # totchol: total cholesterol in mg/dl
  # hdlchol: HDL cholesterol in mg/dl
  # smoke: 0 = non-smoker, 1 = smoker
  # diabetes: 0 = no diabetes, 1 = diabetes


  # for sex=0
  S5_m <- 0.94737
  estimates_m <- c(11.1931,0, 8.9746, -2.1610, -5.4405, 1.2075, 0.8813,
                   0.8302, 4.3491, -0.9637, 0.3526)
  lp_m <- 48.4377


  # for sex=1
  S5_w<- 0.97589
  estimates_w <- c(-17.5033, 3.1733, 5.6155, -1.4054, -5.3616, 1.1326,
                   0.9048, 0.8530, 3.4245, -0.7380, 0.3902)
  lp_w<-  -18.2223


  X <- c(log(age), log(age)^2, log(totchol), log(age)*log(totchol), log(hdlchol),
         log(age)*log(hdlchol), log(SBP)*(SBP_med==1), log(SBP)*(SBP_med==0), smoke,
         log(age)*smoke, diabetes)


  Xbeta<- NULL
  risk<-NULL

  if(sex==0){
    Xbeta <- X %*% estimates_m - lp_m
    risk  <- 1 - S5_m^(exp(Xbeta))
  }
  if(sex==1){
    Xbeta <- X %*% estimates_w - lp_w
    risk <- 1 - S5_w^(exp(Xbeta))
  }

  return(risk)
}
```

Supplementary Figure 1. Compute the ten-year risk from the five-year risk

The Figures below show the hazard (plus 95%-confidence intervals) for the training and the test set for general CVD as defined in the Framingham equations and for ASCVD as defined in the Pooled Cohort Equations. Given the very narrow scale, the assumption of constant hazards seems reasonable.

Training set

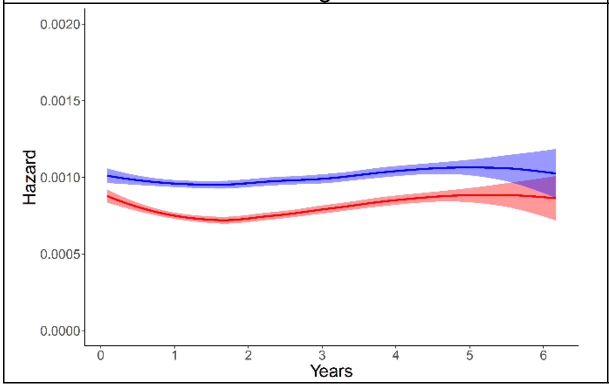

Test set

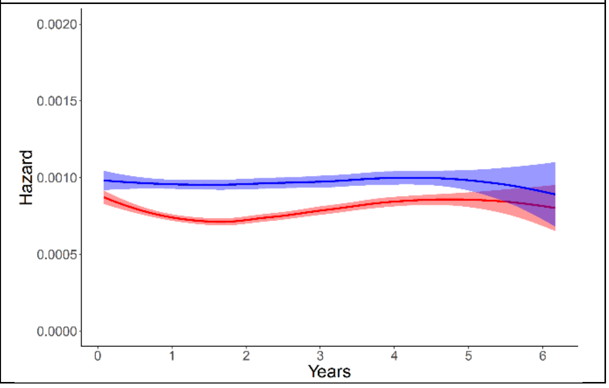

Figure: Plot of smoothed hazards (events/patient years) for a first CVD outcome (red) as predicted by the two Framingham risk equations and for a first ASCVD outcome (blue) as predicted by the Pooled Cohort equations. Shaded areas correspond to 95% pointwise confidence intervals.

If the assumption of constant hazards is reasonable, the risk at five years is assumed to be equal to the distribution function of an exponential distribution $F(t,\lambda) = 1 - e^{-\lambda t}$ with time t and hazard λ. The (assumed constant) hazard is given by $\lambda = f(t,\lambda)/S(t,\lambda)$, where $f(t,\lambda)$ is the density function and $S(t,\lambda)$ is the survivor function. These functions can be expressed as $f(t,\lambda) = \lambda e^{-\lambda t}$ and $S(t,\lambda) = 1 - F(t,\lambda)$. Hence, the hazard can be computed from $F(t)$ as $\lambda = -1/t * \log(1 - F(t))$, and $F(t = 10)$ can be derived from $F(t = 5)$ as $F(t = 10) = 1 - e^{-10\lambda}$. The relation between $F(t = 5)$ and $F(t = 10)$ is visualized in the Figure below.

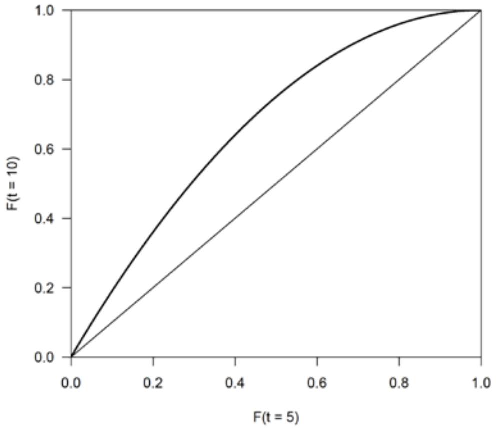

For the cut-offs used in the reclassification table in Table 3 and Supplementary Table 6, the risk at five years corresponds to the following risks at ten years.

Risk at 5 years	Risk at 10 years
0.00%	0.00%
1.25%	2.48%
2.50%	4.94%
3.75%	7.36%
5.00%	9.75%
7.50%	14.44%
10.00%	19.00%

Supplementary Table S3. Baseline characteristics of individuals in the training and test set

Baseline characteristics of 1,122,632 individuals in the training set and 561,380 in the test set attending health screenings in Austria between 1/2009 and 3/2014, aged 30-79, without a history of CVD, and their observed five-year general CVD and ASCVD risk.

Median (interquartile-range, IQR) or n (%) are stated.

	Training set		Test set	
	Women (n=603,671, 53.8%)	Men (n=518,961, 46.2%)	Women (n=302,135, 53.8%)	Men (n=259,245, 46.2%)
**Baseline characteristics**				
Age (years)	50 (41, 62)	50 (41, 61)	50 (41, 62)	50 (41, 61)
Total cholesterol (mmol/L)	5.40 (4.73, 6.18)	5.35 (4.65, 6.08)	5.40 (4.73, 6.15)	5.35 (4.65, 6.10)
HDL cholesterol (mmol/L)	1.60 (1.34, 1.91)	1.27 (1.06, 1.53)	1.60 (1.34, 1.91)	1.27 (1.06, 1.53)
Cholesterol ratio (total/HDL cholesterol)	3.3 (2.7, 4.1)	4.2 (3.4, 5.1)	3.3 (2.7, 4.1)	4.2 (3.4, 5.1)
Systolic BP (mmHg)	125 (115, 140)	130 (120, 141)	125 (115, 140)	130 (120, 142)
BP treatment	85,111 (14.1)	79,693 (15.4)	42,418 (14.0)	39,546 (15.2)
Smoking	121,797 (20.2)	125,264 (24.1)	61,011 (20.2)	62,632 (24.2)
Diabetes	26,767 (4.4)	32,449 (6.3)	13,526 (4.5)	16,461 (6.4)
**Observed five-year risk* in % for CVD as defined by**				
Framingham 1991 general CVD (31,280 events) †	3.28	6.21	3.30	6.10
Framingham 2008 general CVD (32,008 events) †	3.31	6.29	3.32	6.16
ASCVD Pooled Cohort equations (31,009 events)	4.35	7.56	4.28	7.33

* Kaplan-Meier estimate. The observed five-year risk is assessed for individuals who met the inclusion/exclusion criteria of the respective risk equation.

† The two Framingham equations have identical definitions of CVD. However, the inclusion/exclusion criteria are slightly different.

Abbreviations: ASCVD, atherosclerotic cardiovascular disease; BP, blood pressure; CVD, cardiovascular disease; HDL, high-density lipoprotein; IQR, interquartile-range.

Supplementary Table S4. The Austrian study cohort versus the general Austrian population

As part of the latest Austrian Health Interview Survey (2014) Statistics Austria reported characteristics of Austrian inhabitants.[7] Information on smoking status, diabetes, and blood pressure were extracted from the report focusing on Austrian inhabitants aged 15 years or older.
The study cohort included a subsample of individuals between 30 to 79 years. Hence, the numbers should be compared with caution. Furthermore, data from the Austrian Health Interview Survey are self-reported.
Mean and standard deviation (SD) or percentages are given.

Characteristics	WOMEN		MEN	
	**Austrian test set n= 302,135, 53.8%**	**Health survey n= 8,785; 55.7%**	**Austrian test set n=259,245, 46.2%**	**Health survey n= 6,986; 44.3%**
Range of age	30-79	15-	30-79	15-
Age; *mean (SD)*[*]	51.4 (13.1)	48.8 (20.6)	51.1 (12.7)	46.3 (19.4)
Smoking, *%*	20.2	22.1	24.2	26.5
Diabetes, *%*	4.5	4.3	6.4	6.0
High blood pressure, *%*	31.2	20.6	41.2	21.7

[*] obtained by Statistics Austria[7]
Abbreviation: SD, standard deviation.

Relative survival

The figure below shows the relative survival of individuals from the study cohort compared to the general Austrian population.[8]

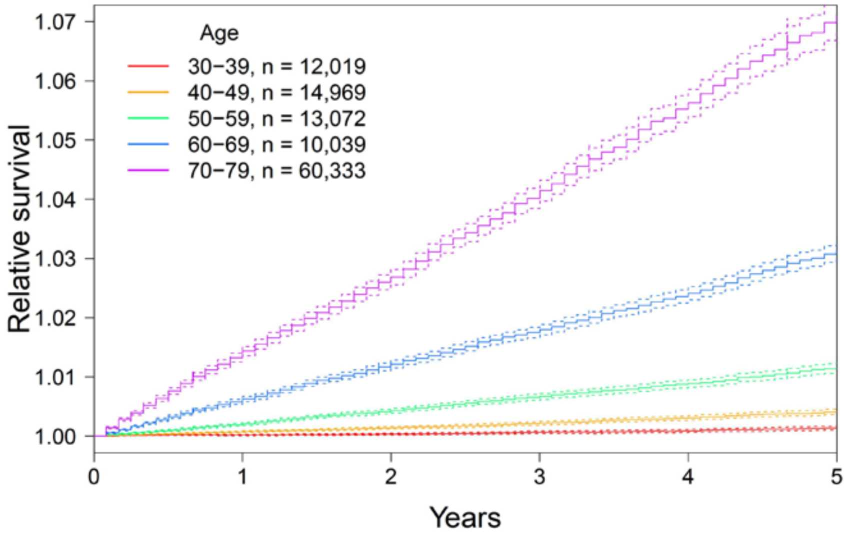

Distribution of age

The two figures below compare (separately for women and men) the distribution of age in the study cohort and the general Austrian population as of 2011.[9]

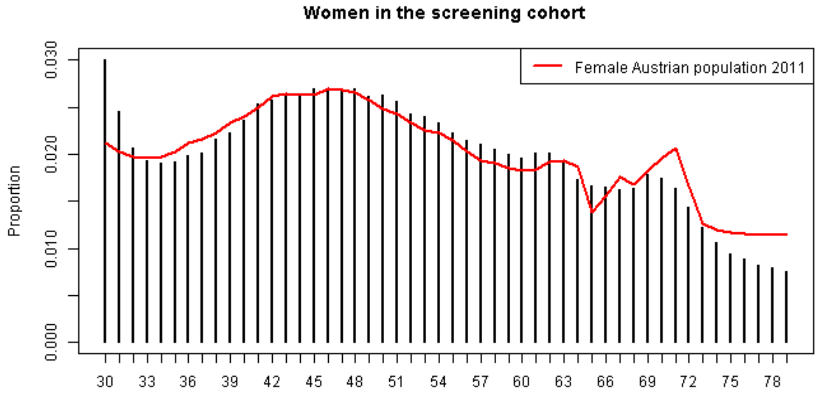

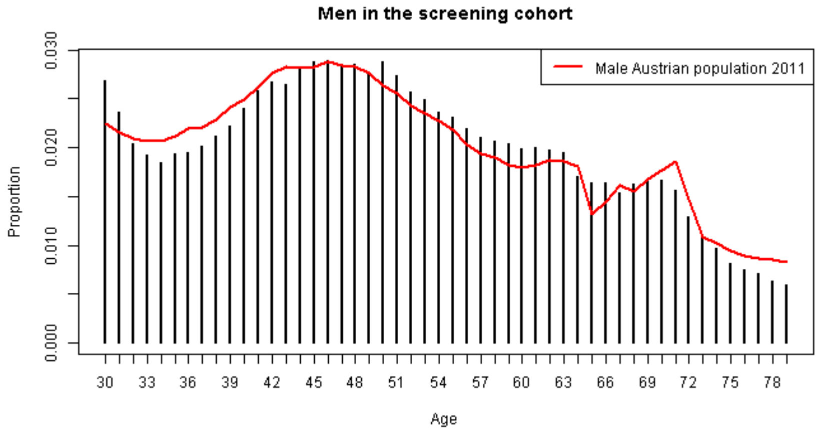

Supplementary Table S5. Calibration in-the-large and calibration slope for the original, recalibrated and re-estimated risk equations.

An optimal calibration-in-the-large is zero, while an optimal calibration slope is one.
Recalibrating an equation by updating the baseline risk does not change the original calibration slope.

Equation	Type	Calibration-in-the large (*100)		Calibration slope	
		Women	Men	Women	Men
Framingham 1991 CVD equation	original	0.119	0.666	1.000	0.997
	re-estimated	-0.163	-0.141	0.997	0.995
Framingham 2008 CVD equation	original	0.448	1.876	0.995	1.016
	recalibrated	-0.091	-0.034		
	re-estimated	-0.112	-0.061	0.992	0.995
Pooled Cohort ASCVD equations	original	-1.947	-1.893	0.819	0.806
	recalibrated	-0.077	0.118		
	re-estimated	-0.026	0.206	0.996	0.998

Supplementary Figure S2: Calibration plots for subgroups

Calibration plots for the five-year risk estimated by the original and the re-estimated equations for individuals in different age groups (first panel), for individuals with/without diabetes (second panel), and for individuals with/without hypertension (third panel) for a) the Framingham 1991, b) the Framingham 2008 equation and c) the Pooled Cohort equations. Error bars represent 95% confidence intervals. Hypertension was defined as a systolic blood pressure ≥140 and/or a diastolic blood pressure ≥90 mmHg.

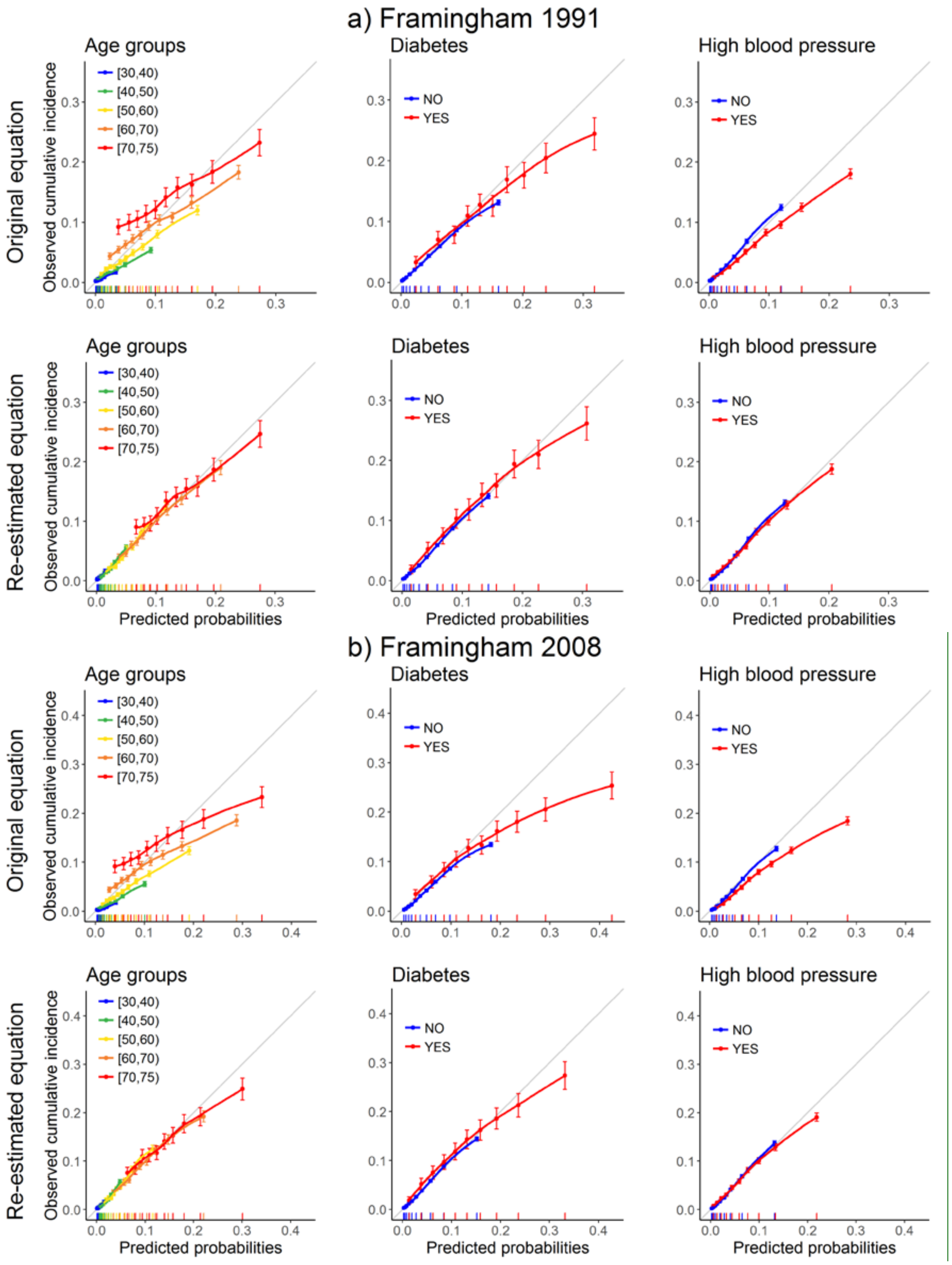

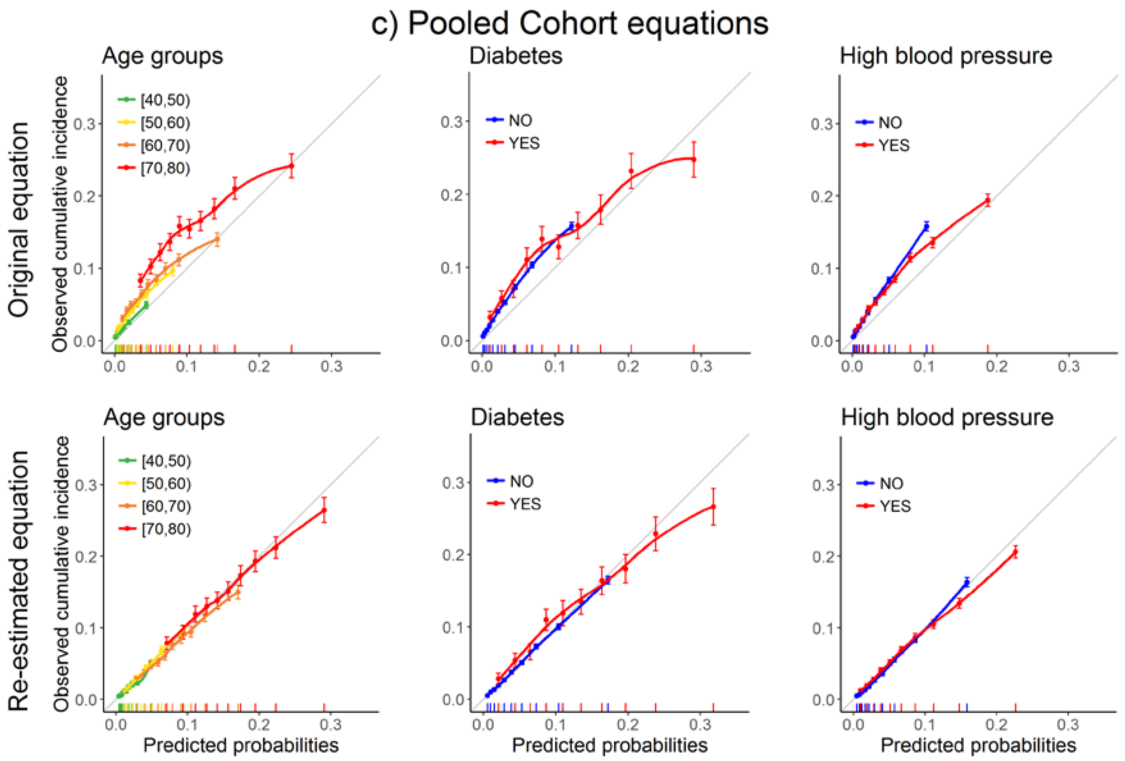
c) Pooled Cohort equations
Original equation
Re-estimated equation
Age groups
Diabetes
High blood pressure
Observed cumulative incidence
Predicted probabilities
[40,50)
[50,60)
[60,70)
[70,80)
NO
YES
0.0
0.1
0.2
0.3

Supplementary Figure S3: Differences in predicted five-year risk between re-estimated and original equations

The Figures below show the differences in the predicted 5-year risk of general CVD for the two Framingham equations and ASCVD for the Pooled Cohort equations for all individuals in the test set (n=561,380) and for all individuals separated for age groups.

```
Distribution of
re-estimated predicted probabilities minus original predicted probabilities
```

	0%	1%	5%	25%	50%	75%	95%	99%	100%
FR1991:	-21.63	-7.52	-4.14	-1.03	-0.13	0.22	2.07	3.70	10.78
FR2008:	-48.34	-10.83	-5.58	-1.77	-0.69	-0.20	1.93	4.29	19.90
PC :	-22.43	-1.05	0.30	0.71	1.34	2.68	6.14	9.58	27.61

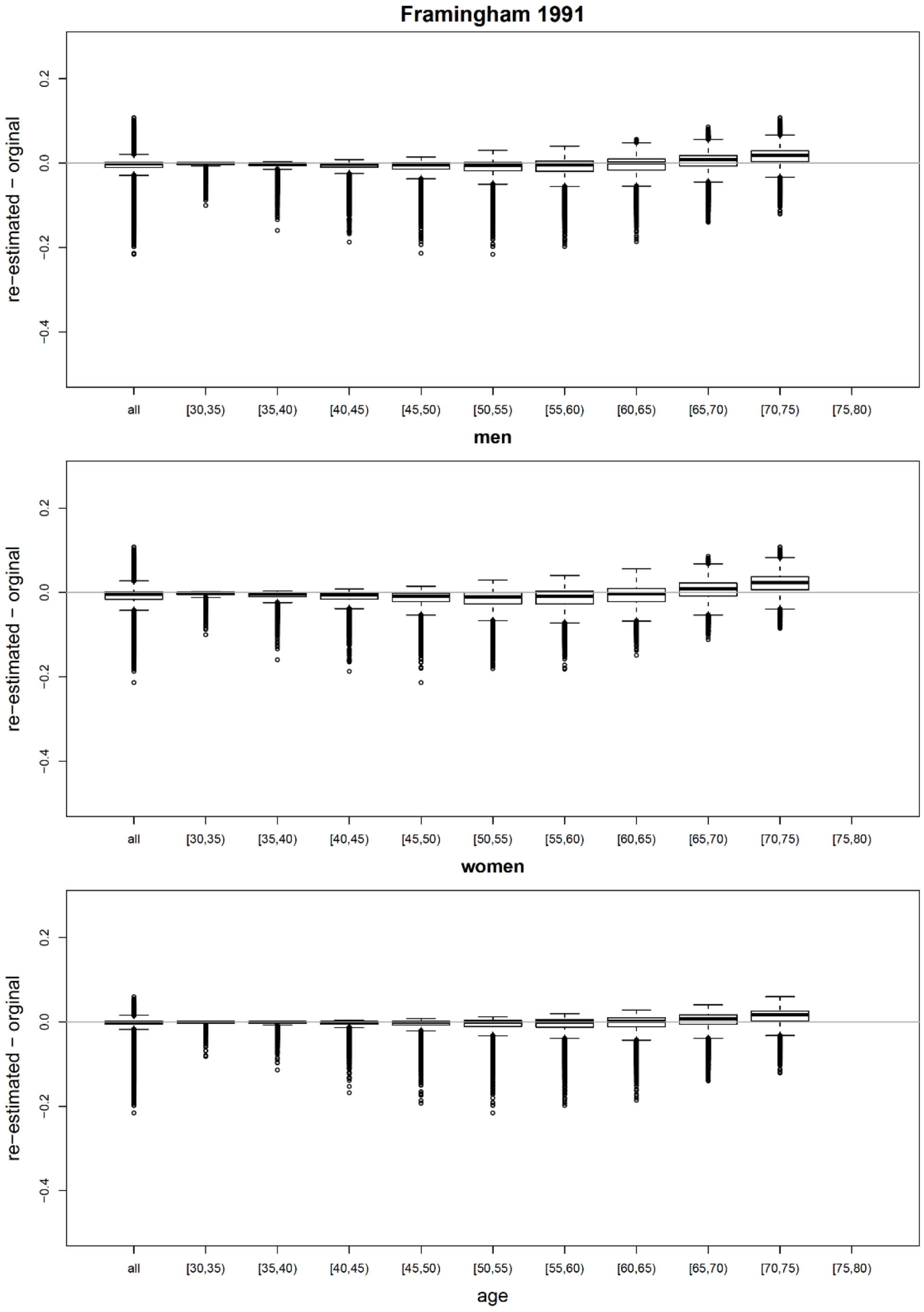

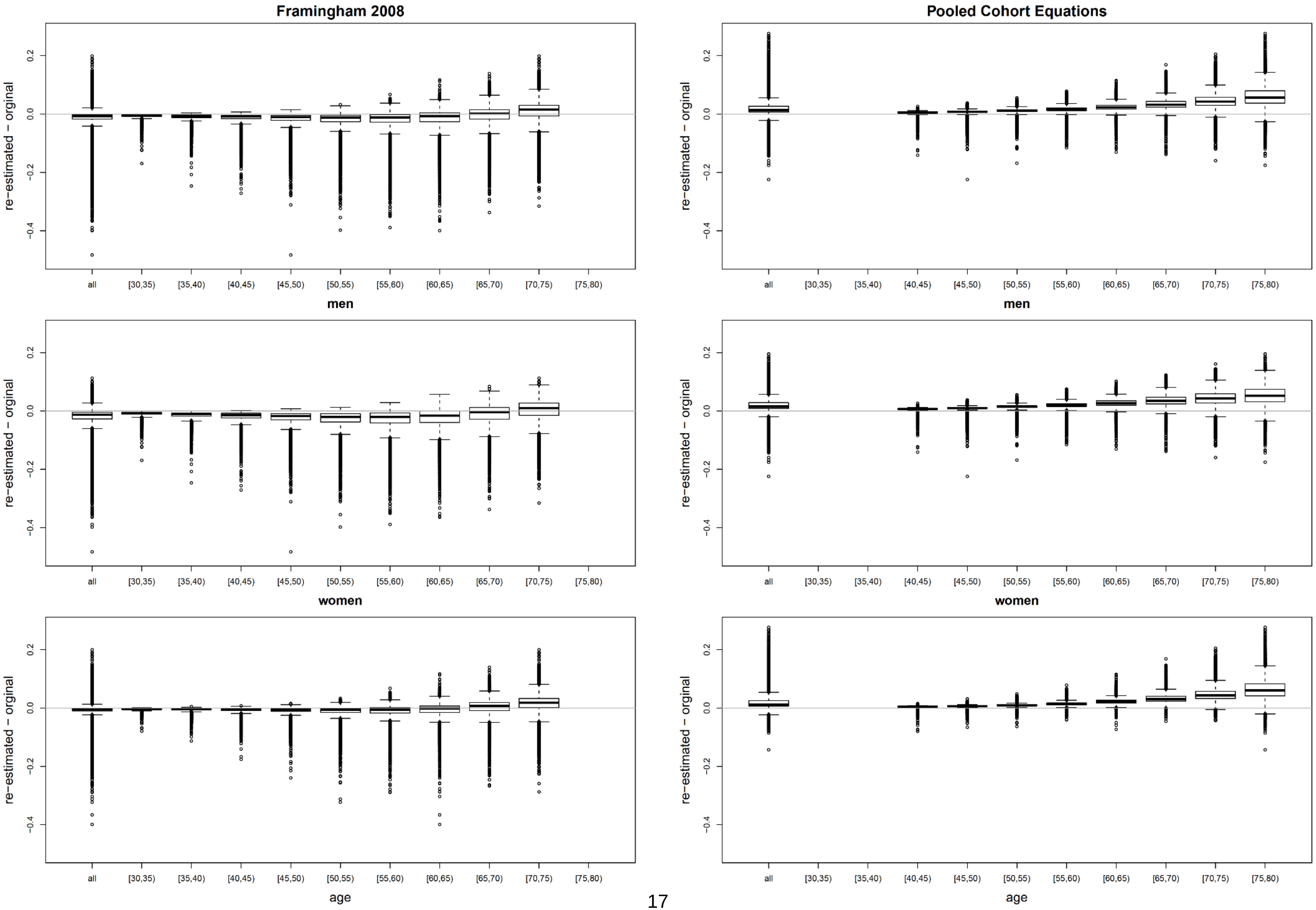
Framingham 2008
Pooled Cohort Equations
re−estimated − orginal
0.2
0.0
−0.2
−0.4
all
[30,35)
[35,40)
[40,45)
[45,50)
[50,55)
[55,60)
[60,65)
[65,70)
[70,75)
[75,80)
men
women
age

Supplementary Table S6: Observed five-year risk of cardiovascular disease (CVD) and atherosclerotic cardiovascular disease (ASCVD)

For the in Table 3 reported risk reclassifications, observed five-year risk in % of CVD based on the Framingham 1991 and 2008 risk equations and ASCVD based on the Pooled Cohort Equations with 95%-confidence intervals are shown. Grey colors indicate the observed 5-year risk of the point estimate. The darker the grey color in a cell, the higher the observed 5-year risk of the individuals classified to this cell.

The observed 5-year risk was computed only for cells with at least 100 observations and at least one event.

Framingham 1991 general CVD

Original equation	Re-estimated equation: <1.25%	1.25 – 2.49%	2.50 – 3.74%	3.75 - 4.99%	5.00 – 7.49%	7.50 - 9.99%	≥ 10.00%	Total
**<1.25%**	0.50% (0.46, 0.55)	1.36% (1.08, 1.64)	4.16% (1.12, 7.10)					0.57% (0.52, 0.62)
**1.25 – 2.49%**	0.96% (0.80, 1.12)	1.69% (1.53, 1.85)	2.70% (2.31, 3.10)	5.41% (4.16, 6.63)	7.32% (3.64, 10.87)			1.71% (1.59, 1.82)
**2.50 – 3.74%**	2.12% (1.27, 2.96)	1.67% (1.45, 1.89)	2.53% (2.27, 2.80)	4.65% (4.09, 5.20)	6.78% (5.82, 7.74)			2.81% (2.64, 2.98)
**3.75 - 4.99%**	1.93% (0.00, 3.86)	2.14% (1.67, 2.59)	2.66% (2.32, 2.99)	3.85% (3.43, 4.27)	6.44% (5.86, 7.02)	12.90% (10.28, 15.43)		4.06% (3.83, 4.29)
**5.00 – 7.49%**		3.19% (2.16, 4.20)	3.11% (2.66, 3.56)	4.46% (4.03, 4.89)	5.86% (5.51, 6.21)	9.25% (8.60, 9.89)	15.60% (12.94, 18.18)	5.87% (5.64, 6.10)
**7.50 - 9.99%**		7.75% (0.00, 15.18)	3.78% (2.48, 5.07)	4.26% (3.47, 5.04)	6.33% (5.82, 6.83)	8.95% (8.37, 9.52)	12.86 (11.94, 13.77)	8.18% (7.85, 8.51)
**≥ 10.00%**			7.16% (1.60, 12.42)	7.23% (4.88, 9.53)	7.36% (6.57, 8.14)	8.97% (8.39, 9.55)	15.15% (14.80, 15.50)	13.37% (13.08, 13.66)
**Total**	0.59% (0.54, 0.64)	1.72% (1.60, 1.83)	2.74% (2.57, 2.91)	4.39% (4.14, 4.63)	6.29% (6.05, 6.53)	9.15% (8.80, 9.49)	14.90% (14.58, 15.23)	4.58% (4.51, 4.66)

Legend

<1	1-2	2-3	3-4	4-5	5-6	6-7	7-8	8-9	9-10	10-11	11-12	12-13	13-14	14-15	15-16	16-17	17-18

Re-estimated equation

Framingham 2008 general CVD

Original equation								
**<1.25%**	0.41% (0.36, 0.46)	1.29% (0.64, 1.94)						0.43% (0.38, 0.48)
**1.25 – 2.49%**	0.77% (0.68, 0.86)	1.44% (1.29, 1.60)	2.97% (2.32, 3.61)	5.24% (3.43, 7.02)	11.97% (6.84, 16.82)			1.18% (1.09, 1.26)
**2.50 – 3.74%**	1.04% (0.75, 1.33)	1.66% (1.49, 1.84)	2.58% (2.28, 2.88)	4.82% (4.11, 5.53)	6.98% (5.80, 8.15)	14.34% (8.84, 19.50)		2.33% (2.19, 2.47)
**3.75 - 4.99%**	3.50% (1.59, 5.38)	1.95% (1.66, 2.23)	2.78% (2.48, 3.07)	4.15% (3.67, 4.62)	5.88% (5.23, 6.53)	10.09% (8.19, 11.95)	17.63% (10.47, 24.22)	3.53% (3.33, 3.73)
**5.00 – 7.49%**		2.72% (2.10, 3.34)	2.83% (2.50, 3.16)	4.21% (3.84, 4.59)	5.87% (5.48, 6.25)	9.93% (9.14, 10.71)	12.13% (10.54, 13.69)	5.28% (5.08, 5.49)
**7.50 - 9.99%**		3.89% (1.33, 6.39)	3.09% (2.28, 3.89)	5.06% (4.38, 5.73)	6.36% (5.88, 6.84)	9.10% (8.45, 9.75)	12.74% (11.81, 13.67)	7.75% (7.44, 8.07)
**≥ 10.00%**			6.98% (3.65, 10.19)	5.85% (4.48, 7.20)	7.57% (6.92, 8.21)	8.54% (8.02, 9.06)	15.41% (15.07, 15.76)	13.11% (12.84, 13.38)
**Total**	0.58% (0.54, 0.63)	1.67% (1.57, 1.78)	2.79% (2.62, 2.96)	4.49% (4.24, 4.73)	6.37% (6.13, 6.62)	9.15% (8.79, 9.50)	15.05% (14.73, 15.36)	4.63% (4.56, 4.70)

Pooled Cohort ASCVD Equations

Original equation								
**<1.25%**	0.78% (0.71, 0.86)	1.56% (1.45, 1.67)	2.77% (2.40, 3.13)	4.07% (2.01, 6.09)				1.28% (1.21, 1.35)
**1.25 – 2.49%**		2.26% (1.93, 2.60)	2.71% (2.52, 2.91)	4.50% (4.16, 4.85)	5.71% (5.01, 6.41)	7.17% (2.91, 11.24)		3.36% (3.20, 3.51)
**2.50 – 3.74%**			3.75% (3.06, 4.44)	4.23% (3.86, 4.61)	5.50% (5.15, 5.85)	9.74% (8.55, 10.92)	14.15% (9.67, 18.40)	5.278% (5.034, 5.52)
**3.75 - 4.99%**			5.39% (0.44, 10.10)	4.68% (3.67, 5.67)	6.16% (5.73, 6.59)	8.06% (7.42, 8.70)	13.34% (11.66, 14.99)	7.10% (6.76, 7.44)
**5.00 – 7.49%**				4.32% (1.95, 6.62)	6.99% (6.32, 7.65)	8.28% (7.81, 8.75)	11.84% (11.24, 12.44)	9.39% (9.06, 9.72)
**7.50 - 9.99%**					8.85% (6.02, 11.58)	9.92% (8.77, 11.06)	13.64% (13.10, 14.18)	13.02% (12.54, 13.51)
**≥ 10.00%**						14.59% (11.09, 17.96)	17.51% (17.08, 17.94)	17.46% (17.03, 17.89)
**Total**	0.78% (0.71, 0.86)	1.65% (1.55, 1.76)	2.82% (2.6%, 2.98)	4.40% (4.15, 4.64)	6.00% (5.76, 6.23)	8.62% (8.28, 8.97)	15.24% (14.95, 15.53)	5.70% (5.61, 5.78)

Supplementary Table S7: Risk reclassification tables for subgroups

Reclassification tables for the risk at five years for the three evaluated equations separated for women and men, for individuals of different age groups, and for individuals with and without diabetes and hypertension are shown. According to the inclusion/exclusion criteria of the respective risk equations, the results of the two Framingham equations are given for individuals from 30 to 75 years, while the Pooled Cohort Equations cover individuals from 40 to 79.

Reclassifications of the re-estimated Framingham 1991 equations versus the re-estimated Framingham 2008 equations are presented, as well.

Assuming constant hazards, the risk at ten years can be extrapolated from the predicted risk at five years (for details see Supplementary Figure 1). For the risk categories shown here, the ten-year risk is roughly twice the five-year risk.

Additionally, the percentage of individuals, who remained in the same risk category, who were up-classified, or who were down-classified are given.

Below each reclassification table, the observed 5-year risk with 95%-confidence intervals is also shown. The observed 5-year risk was computed only for cells with at least 100 observations and at least one event. Different shades of grey indicate the value of the observed 5-year risk. The darker the grey, the higher the observed risk.

Framingham 1991 general CVD – MEN

Reclassification table

Original equation	Re-estimated equation: **<1.25%**	**1.25 – 2.49%**	**2.50 – 3.74%**	**3.75 - 4.99%**	**5.00 – 7.49%**	**7.50 - 9.99%**	**≥ 10.00%**	**Total**
**<1.25%**	43,861 (18.1%)	2,656 (1.1%)	32 (0.0%)	0 (0.0%)	0 (0.0%)	0 (0.0%)	0 (0.0%)	46,549 (19.2%)
**1.25 – 2.49%**	12,581 (5.2%)	18,347 (7.6%)	2,750 (1.1%)	216 (0.1%)	21 (0.0%)	0 (0.0%)	0 (0.0%)	33,915 (14.0%)
**2.50 – 3.74%**	1,354 (0.6%)	11,777 (4.9%)	9,281 (3.8%)	2,398 (1.0%)	556 (0.2%)	24 (0.0%)	0 (0.0%)	25,390 (10.5%)
**3.75 - 4.99%**	152 (0.1%)	3,766 (1.6%)	7,960 (3.3%)	5,495 (2.3%)	2,806 (1.2%)	301 (0.1%)	10 (0.0%)	20,490 (8.4%)
**5.00 – 7.49%**	19 (0.0%)	1,139 (0.5%)	5,626 (2.3%)	8,657 (3.6%)	11,772 (4.8%)	3,991 (1.6%)	953 (0.4%)	32,157 (13.2%)
**7.50 - 9.99%**	0 (0.0%)	59 (0.0%)	780 (0.3%)	2,653 (1.1%)	8,822 (3.6%)	7,054 (2.9%)	5,063 (2.1%)	24,431 (10.1%)
**≥ 10.00%**	0 (0.0%)	5 (0.0%)	91 (0.0%)	473 (0.2%)	4,543 (1.9%)	9,129 (3.8%)	45,593 (18.8%)	59,834 (24.6%)
**Total**	57,967 (23.9%)	37,749 (15.5%)	26,520 (10.9%)	19,892 (8.2%)	28,520 (11.7%)	20,499 (8.4%)	51,619 (21.3%)	242,766 (100%)

Reclassification **NO: 58.2%** **UP: 9.0%** **DOWN: 32.8%**

5-year Kaplan Meier estimate of general CVD with 95% confidence intervals

Original equation	<1.25%	1.25 – 2.49%	2.50 – 3.74%	3.75 - 4.99%	5.00 – 7.49%	7.50 - 9.99%	≥ 10.00%	Total
**<1.25%**	0.48% (0.40, 0.57)	1.62% (1.00, 2.23)						0.56% (0.46, 0.65)
**1.25 – 2.49%**	0.73% (0.54, 0.92)	1.64% (1.40, 1.88)	2.28% (1.62, 2.94)	5.41% (2.24, 8.48)				1.40% (1.24, 1.56)
**2.50 – 3.74%**	1.31% (0.58, 2.04)	1.64% (1.34, 1.95)	2.57% (2.18, 2.97)	4.77% (3.70, 5.84)	7.88% (5.01, 10.67)			2.43% (2.19, 2.68)
**3.75 - 4.99%**	1.02% (0.00, 3.00)	1.86% (1.28, 2.43)	2.72% (2.26, 3.18)	3.86% (3.23, 4.49)	5.49% (4.45, 6.52)	17.31% (11.68, 22.58)		3.50% (3.18, 3.83)
**5.00 – 7.49%**		3.85% (2.36, 5.32)	2.72% (2.18, 3.26)	4.54% (3.97, 5.11)	5.76% (5.24, 6.27)	9.97% (8.87, 11.05)	15.48% (12.70, 18.16)	5.74% (5.43, 6.08)
**7.50 - 9.99%**			3.69% (2.01, 5.35)	4.62% (3.57, 5.65)	6.42% (5.75, 7.08)	8.81% (8.01, 9.61)	13.66% (12.54, 14.76)	8.46% (8.02, 8.90)
**≥ 10.00%**				8.48% (5.13, 11.72)	7.46% (6.46, 8.45)	9.45% (8.70, 10.19)	15.49% (15.09, 15.89)	13.97% (13.64, 14.31)
**Total**	0.56% (0.48, 0.64)	1.73% (1.56, 1.90)	2.66% (2.41, 2.90)	4.49% (4.12, 4.86)	6.22% (5.87, 6.58)	9.46% (8.97, 9.95)	15.30% (14.93, 15.67)	6.10% (5.98, 6.22)

Legend: Observed 5-year risk (in %) for CVD and ASCVD

<1	1-2	2-3	3-4	4-5	5-6	6-7	7-8	8-9	9-10	10-11	11-12	12-13	13-14	14-15	15-16	16-17	17-18

Framingham 1991 general CVD - WOMEN

Reclassification table

Original equation	Re-estimated equation: <1.25%	1.25 – 2.49%	2.50 – 3.74%	3.75 - 4.99%	5.00 – 7.49%	7.50 - 9.99%	≥ 10.00%	Total
**<1.25%**	106,102 (37.5%)	8,249 (2.9%)	247 (0.1%)	10 (0.0%)	0 (0.0%)	0 (0.0%)	0 (0.0%)	114,608 (40.5%)
**1.25 – 2.49%**	11,716 (4.1%)	25,556 (9.0%)	7,351 (2.6%)	1,423 (0.5%)	253 (0.1%)	0 (0.0%)	0 (0.0%)	46,299 (16.4%)
**2.50 – 3.74%**	1,000 (0.4%)	9,805 (3.5%)	11,310 (4.0%)	5,861 (2.1%)	3,093 (1.1%)	7 (0.0%)	0 (0.0%)	31,076 (11.0%)
**3.75 - 4.99%**	130 (0.0%)	2,505 (0.9%)	6,404 (2.3%)	6,481 (2.3%)	7,232 (2.6%)	700 (0.2%)	1 (0.0%)	23,453 (8.3%)
**5.00 – 7.49%**	11 (0.0%)	763 (0.3%)	3,571 (1.3%)	6,139 (2.2%)	13,101 (4.6%)	6,953 (2.5%)	121 (0.0%)	30,659 (10.8%)
**7.50 - 9.99%**	1 (0.0%)	54 (0.0%)	480 (0.2%)	1,453 (0.5%)	5,488 (1.9%)	6,583 (2.3%)	2,138 (0.8%)	16,197 (5.7%)
**≥ 10.00%**	0 (0.0%)	4 (0.0%)	69 (0.0%)	324 (0.1%)	2,387 (0.8%)	4,910 (1.7%)	12,746 (4.5%)	20,440 (7.2%)
**Total**	118,960 (42.1%)	46,936 (16.6%)	29,432 (10.4%)	21,691 (7.7%)	31,554 (11.2%)	19,153 (6.8%)	15,006 (5.3%)	28,2732 (100%)

**Reclassification**	**NO: 64.4%**	**UP: 15.4%**	**DOWN: 20.3%**

5-year Kaplan Meier estimate of general CVD with 95% confidence intervals

Original equation	<1.25%	1.25 – 2.49%	2.50 – 3.74%	3.75 - 4.99%	5.00 – 7.49%	7.50 - 9.99%	≥ 10.00%	Total
**<1.25%**	0.51% (0.45, 0.56)	1.28% (0.97, 1.58)	4.64% (1.26, 7.90)					0.58% (0.52, 0.63)
**1.25 – 2.49%**	1.19% (0.94, 1.44)	1.72% (1.52, 1.93)	2.86% (2.38, 3.33)	5.40% (4.05, 6.73)	7.41% (3.55, 11.12)			1.93% (1.77, 2.08)
**2.50 – 3.74%**	3.06% (1.43, 4.67)	1.71% (1.39, 2.03)	2.50% (2.15, 2.86)	4.6% (3.95, 5.25)	6.59% (5.57, 7.60)			3.1% (2.87, 3.34)
**3.75 - 4.99%**	2.98% (0.00, 6.38)	2.54% (1.78, 3.30)	2.59% (2.11, 3.06)	3.83% (3.27, 4.39)	6.79% (6.10, 7.49)	10.88% (8.05, 13.61)		4.52% (4.20, 4.84)
**5.00 – 7.49%**		2.22% (0.93, 3.50)	3.70% (2.90, 4.49)	4.35% (3.70, 4.99)	5.94% (5.46, 6.43)	8.82% (8.02, 9.61)	17.22% (7.27, 26.09)	6.00% (5.67, 6.32)
**7.50 - 9.99%**			3.92% (1.84, 5.96)	3.62% (2.45, 4.78)	6.22% (5.45, 6.99)	9.08% (8.24, 9.91)	10.87% (9.26, 12.46)	7.77% (7.27, 8.27)
**≥ 10.00%**				5.36% (2.29, 8.33)	7.18% (5.88, 8.46)	8.14% (7.22, 9.06)	13.91% (13.18, 14.64)	11.61% (11.07, 12.15)
**Total**	0.6% (0.55, 0.66)	1.7% (1.55, 1.85)	2.81% (2.57, 3.04)	4.3% (3.97, 4.63)	6.36% (6.03, 6.68)	8.82% (8.33, 9.30)	13.5% (12.83, 14.16)	3.3% (3.21, 3.38)

Legend: Observed 5-year risk (in %) for CVD and ASCVD

<1	1-2	2-3	3-4	4-5	5-6	6-7	7-8	8-9	9-10	10-11	11-12	12-13	13-14	14-15	15-16	16-17	17-18

Framingham 1991 general CVD – AGE 30 to 39

Reclassification table

Re-estimated equation

Original equation	<1.25%	1.25 – 2.49%	2.50 – 3.74%	3.75 - 4.99%	5.00 – 7.49%	7.50 - 9.99%	≥ 10.00%	Total
**<1.25%**	97,814 (81.1%)	6 (0.0%)	0 (0.0%)	0 (0.0%)	0 (0.0%)	0 (0.0%)	0 (0.0%)	97,820 (81.1%)
**1.25 – 2.49%**	13,899 (11.5%)	1,135 (0.9%)	0 (0.0%)	0 (0.0%)	0 (0.0%)	0 (0.0%)	0 (0.0%)	15,034 (12.5%)
**2.50 – 3.74%**	1,996 (1.7%)	2,467 (2.0%)	2 (0.0%)	0 (0.0%)	0 (0.0%)	0 (0.0%)	0 (0.0%)	4,465 (3.7%)
**3.75 - 4.99%**	268 (0.2%)	1,592 (1.3%)	18 (0.0%)	0 (0.0%)	0 (0.0%)	0 (0.0%)	0 (0.0%)	1,878 (1.6%)
**5.00 – 7.49%**	30 (0.0%)	907 (0.8%)	174 (0.1%)	2 (0.0%)	0 (0.0%)	0 (0.0%)	0 (0.0%)	1,113 (0.9%)
**7.50 - 9.99%**	1 (0.0%)	82 (0.1%)	143 (0.1%)	15 (0.0%)	0 (0.0%)	0 (0.0%)	0 (0.0%)	241 (0.2%)
**≥ 10.00%**	0 (0.0%)	8 (0.0%)	65 (0.1%)	21 (0.0%)	11 (0.0%)	0 (0.0%)	0 (0.0%)	105 (0.1%)
**Total**	114,008 (94.5%)	6,197 (5.1%)	402 (0.3%)	38 (0.0%)	11 (0.0%)	0 (0.0%)	0 (0.0%)	120,656 (100%)

Reclassification **NO: 82.0%** **UP: <0.1%** **DOWN: 18.0%**

5-year Kaplan Meier estimate of general CVD with 95% confidence intervals

Original equation	<1.25%	1.25 – 2.49%	2.50 – 3.74%	3.75 - 4.99%	5.00 – 7.49%	7.50 - 9.99%	≥ 10.00%	Total
**<1.25%**	0.39% (0.33, 0.44)							0.39% (0.33, 0.44)
**1.25 – 2.49%**	0.92% (0.71, 1.12)	1.77% (0.66, 2.86)						0.98% (0.78, 1.19)
**2.50 – 3.74%**	1.82% (0.99, 2.65)	1.46% (0.80, 2.11)						1.62% (1.10, 2.13)
**3.75 - 4.99%**	1.64% (0.00, 3.52)	1.93% (1.08, 2.78)						1.87% (1.10, 2.64)
**5.00 – 7.49%**		3.38% (1.85, 4.88)	3.27% (0.37, 6.09)					3.37% (2.03, 4.69)
**7.50 - 9.99%**			0.78% (0.00, 2.29)					3.25% (0.00, 6.50)
**≥ 10.00%**								4.46% (0.00, 9.75)
**Total**	0.48% (0.43, 0.54)	2% (1.53, 2.47)	2.25% (0.49, 3.98)					0.57% (0.52, 0.63)

Legend: Observed 5-year risk (in %) for CVD and ASCVD

<1	1-2	2-3	3-4	4-5	5-6	6-7	7-8	8-9	9-10	10-11	11-12	12-13	13-14	14-15	15-16	16-17	17-18

Framingham 1991 general CVD – AGE 40 to 49

Reclassification table

Original equation	Re-estimated equation: **<1.25%**	**1.25 – 2.49%**	**2.50 – 3.74%**	**3.75 - 4.99%**	**5.00 – 7.49%**	**7.50 - 9.99%**	**≥ 10.00%**	**Total**
**<1.25%**	50,282 (34.2%)	3,586 (2.4%)	0 (0.0%)	0 (0.0%)	0 (0.0%)	0 (0.0%)	0 (0.0%)	53,868 (36.6%)
**1.25 – 2.49%**	10,398 (7.1%)	27,396 (18.6%)	348 (0.2%)	0 (0.0%)	0 (0.0%)	0 (0.0%)	0 (0.0%)	38,142 (25.9%)
**2.50 – 3.74%**	358 (0.2%)	15,924 (10.8%)	5,186 (3.5%)	41 (0.0%)	1 (0.0%)	0 (0.0%)	0 (0.0%)	21,510 (14.6%)
**3.75 - 4.99%**	14 (0.0%)	4,375 (3.0%)	7,214 (4.9%)	594 (0.4%)	1 (0.0%)	0 (0.0%)	0 (0.0%)	12,198 (8.3%)
**5.00 – 7.49%**	0 (0.0%)	994 (0.7%)	7,005 (4.8%)	3,551 (2.4%)	440 (0.3%)	1 (0.0%)	0 (0.0%)	11,991 (8.2%)
**7.50 - 9.99%**	0 (0.0%)	31 (0.0%)	1,038 (0.7%)	2,448 (1.7%)	1,603 (1.1%)	15 (0.0%)	0 (0.0%)	5,135 (3.5%)
**≥ 10.00%**	0 (0.0%)	1 (0.0%)	95 (0.1%)	684 (0.5%)	2,549 (1.7%)	653 (0.4%)	211 (0.1%)	4,193 (2.9%)
**Total**	61,052 (41.5%)	52,307 (35.6%)	20,886 (14.2%)	7,318 (5.0%)	4,594 (3.1%)	669 (0.5%)	211 (0.1%)	147,037 (100%)

Reclassification **NO: 57.2%** **UP: 2.7%** **DOWN: 40.1%**

5-year Kaplan Meier estimate of general CVD with 95% confidence intervals

Original equation	**<1.25%**	**1.25 – 2.49%**	**2.50 – 3.74%**	**3.75 - 4.99%**	**5.00 – 7.49%**	**7.50 - 9.99%**	**≥ 10.00%**	**Total**
**<1.25%**	0.68% (0.59, 0.77)	1.47% (0.96, 1.98)						0.73% (0.64, 0.82)
**1.25 – 2.49%**	1.01% (0.77, 1.26)	1.5% (1.32, 1.68)	1.7% (0.18, 3.19)					1.37% (1.22, 1.52)
**2.50 – 3.74%**	3.72% (0.60, 6.73)	1.59% (1.34, 1.83)	2.59% (2.07, 3.11)					1.88% (1.65, 2.11)
**3.75 - 4.99%**		2.16% (1.60, 2.72)	2.69% (2.20, 3.18)	4.21% (2.21, 6.17)				2.58% (2.21, 2.95)
**5.00 – 7.49%**		3.01% (1.63, 4.38)	2.91% (2.41, 3.41)	4.11% (3.29, 4.93)	3.23% (1.33, 5.10)			3.28% (2.88, 3.68)
**7.50 - 9.99%**			4.10% (2.60, 5.58)	4.47% (3.45, 5.47)	6.49% (4.93, 8.03)			5.03% (4.28, 5.78)
**≥ 10.00%**				7.85% (5.16, 10.47)	7.78% (6.42, 9.12)	11.03% (7.67, 14.26)	15.37% (8.96, 21.32)	8.72% (7.59, 9.83)
**Total**	0.75% (0.67, 0.84)	1.61% (1.47, 1.75)	2.82% (2.54, 3.10)	4.63% (4.02, 5.24)	6.86% (5.92, 7.79)	10.89% (7.62, 14.05)	15.37% (8.96, 21.32)	1.79% (1.71, 1.88)

Legend: Observed 5-year risk (in %) for CVD and ASCVD

<1	1-2	2-3	3-4	4-5	5-6	6-7	7-8	8-9	9-10	10-11	11-12	12-13	13-14	14-15	15-16	16-17	17-18

Framingham 1991 general CVD – AGE50 to 59

Reclassification table

Original equation		Re-estimated equation							
		**<1.25%**	**1.25 – 2.49%**	**2.50 – 3.74%**	**3.75 - 4.99%**	**5.00 – 7.49%**	**7.50 - 9.99%**	**≥ 10.00%**	**Total**
	**<1.25%**	1,867 (1.5%)	7,209 (5.6%)	38 (0.0%)	0 (0.0%)	0 (0.0%)	0 (0.0%)	0 (0.0%)	9,114 (7.1%)
	**1.25 – 2.49%**	0 (0.0%)	15,371 (12.0%)	7,010 (5.5%)	184 (0.1%)	0 (0.0%)	0 (0.0%)	0 (0.0%)	22,565 (17.7%)
	**2.50 – 3.74%**	0 (0.0%)	3,191 (2.5%)	14,448 (11.3%)	2,930 (2.3%)	116 (0.1%)	0 (0.0%)	0 (0.0%)	20,685 (16.2%)
	**3.75 - 4.99%**	0 (0.0%)	304 (0.2%)	7,132 (5.6%)	7,983 (6.3%)	1,798 (1.4%)	3 (0.0%)	0 (0.0%)	17,220 (13.5%)
	**5.00 – 7.49%**	0 (0.0%)	1 (0.0%)	2,018 (1.6%)	10,494 (8.2%)	11,113 (8.7%)	273 (0.2%)	0 (0.0%)	23,899 (18.7%)
	**7.50 - 9.99%**	0 (0.0%)	0 (0.0%)	79 (0.1%)	1,643 (1.3%)	9,883 (7.7%)	2,417 (1.9%)	115 (0.1%)	14,137 (11.1%)
	**≥ 10.00%**	0 (0.0%)	0 (0.0%)	0 (0.0%)	92 (0.1%)	3,978 (3.1%)	7,984 (6.3%)	7,998 (6.3%)	20,052 (15.7%)
	**Total**	1,867 (1.5%)	26,076 (20.4%)	30,725 (24.1%)	23,326 (18.3%)	26,888 (21.1%)	10,677 (8.4%)	8,113 (6.4%)	127,672 (100%)

**Reclassification**	**NO: 47.9%**	**UP: 15.4%**	**DOWN: 36.7%**

5-year Kaplan Meier estimate of general CVD with 95% confidence intervals

Original equation		<1.25%	1.25 – 2.49%	2.50 – 3.74%	3.75 - 4.99%	5.00 – 7.49%	7.50 - 9.99%	≥ 10.00%	Total
	**<1.25%**	1.17% (0.53, 1.80)	1.19% (0.88, 1.50)						1.18% (0.91, 1.46)
	**1.25 – 2.49%**		2.02% (1.73, 2.31)	2.54% (2.09, 3.00)	6.35% (2.63, 9.92)				2.22% (1.98, 2.47)
	**2.50 – 3.74%**		2.27% (1.59, 2.95)	2.48% (2.17, 2.80)	4.52% (3.59, 5.45)	5.1% (0.00, 10.24)			2.76% (2.48, 3.03)
	**3.75 - 4.99%**		2.65% (0.65, 4.62)	2.63% (2.18, 3.08)	3.50% (3.00, 4.00)	5.27% (4.00, 6.53)			3.32% (2.99, 3.64)
	**5.00 – 7.49%**			3.77% (2.67, 4.86)	4.62% (4.10, 5.14)	5.49% (4.97, 6.01)	10.52% (6.07, 14.76)		5.03% (4.68, 5.38)
	**7.50 - 9.99%**				3.98% (2.71, 5.23)	6.29% (5.67, 6.90)	8.95% (7.53, 10.35)	12.47% (4.11, 20.09)	6.53% (6.01, 7.05)
	**≥ 10.00%**					7.51% (6.46, 8.54)	9.24% (8.45, 10.03)	13.75% (12.82, 14.67)	10.68% (10.15, 11.21)
	**Total**	1.17% (0.53, 1.80)	1.83% (1.62, 2.04)	2.62% (2.40, 2.84)	4.18% (3.85, 4.51)	6.05% (5.70, 6.41)	9.21% (8.53, 9.89)	13.73% (12.80, 14.65)	4.7% (4.55, 4.84)

Legend: Observed 5-year risk (in %) for CVD and ASCVD

<1	1-2	2-3	3-4	4-5	5-6	6-7	7-8	8-9	9-10	10-11	11-12	12-13	13-14	14-15	15-16	16-17	17-18

Framingham 1991 general CVD – AGE 60 to 69

Reclassification table

		Re-estimated equation							
		**<1.25%**	**1.25 – 2.49%**	**2.50 – 3.74%**	**3.75 - 4.99%**	**5.00 – 7.49%**	**7.50 - 9.99%**	**≥ 10.00%**	**Total**
**Original equation**	**<1.25%**	0 (0.0%)	104 (0.1%)	240 (0.3%)	2 (0.0%)	0 (0.0%)	0 (0.0%)	0 (0.0%)	346 (0.4%)
	**1.25 – 2.49%**	0 (0.0%)	1 (0.0%)	2,743 (2.9%)	1,405 (1.5%)	67 (0.1%)	0 (0.0%)	0 (0.0%)	4,216 (4.5%)
	**2.50 – 3.74%**	0 (0.0%)	0 (0.0%)	955 (1.0%)	5,288 (5.6%)	2,372 (2.5%)	10 (0.0%)	0 (0.0%)	8,625 (9.1%)
	**3.75 - 4.99%**	0 (0.0%)	0 (0.0%)	0 (0.0%)	3,399 (3.6%)	6,537 (6.9%)	244 (0.3%)	0 (0.0%)	10,180 (10.8%)
	**5.00 – 7.49%**	0 (0.0%)	0 (0.0%)	0 (0.0%)	749 (0.8%)	12,906 (13.6%)	5,503 (5.8%)	370 (0.4%)	19,528 (20.6%)
	**7.50 - 9.99%**	0 (0.0%)	0 (0.0%)	0 (0.0%)	0 (0.0%)	2,824 (3.0%)	8,917 (9.4%)	3,583 (3.8%)	15,324 (16.2%)
	**≥ 10.00%**	0 (0.0%)	0 (0.0%)	0 (0.0%)	0 (0.0%)	392 (0.4%)	5,270 (5.6%)	30,762 (32.5%)	36,424 (38.5%)
	**Total**	0 (0.0%)	105 (0.1%)	3,938 (4.2%)	10,843 (11.5%)	25,098 (26.5%)	19,944 (21.1%)	34,715 (36.7%)	94,643 (100%)

**Reclassification**	**NO: 60.2%**	**UP: 30.0%**	**DOWN: 9.8%**

5-year Kaplan Meier estimate of general CVD with 95% confidence intervals

**Original equation**	**<1.25%**	9.38% (0.82, 17.20)	4.76% (1.30, 8.10)					6.05% (2.62, 9.36)	9.38% (0.82, 17.20)
	**1.25 – 2.49%**		3.22% (2.38, 4.05)	4.99% (3.68, 6.29)				3.92% (3.20, 4.62)	
	**2.50 – 3.74%**		2.94% (1.68, 4.19)	4.68% (3.98, 5.37)	5.92% (4.81, 7.03)			4.86% (4.31, 5.40)	
	**3.75 - 4.99%**			4.56% (3.72, 5.38)	6.03% (5.32, 6.72)	16.64% (10.56, 22.30)		5.79% (5.24, 6.34)	
	**5.00 – 7.49%**			3.78% (1.94, 5.58)	6.10% (5.61, 6.59)	9.18% (8.28, 10.07)	14.72% (10.46, 18.78)	7.07% (6.64, 7.50)	
	**7.50 - 9.99%**				6.40% (5.30, 7.48)	8.63% (7.92, 9.32)	13.21% (11.90, 14.50)	9.33% (8.78, 9.88)	
	**≥ 10.00%**				3.42% (0.97, 5.81)	8.37% (7.46, 9.26)	14.23% (13.76, 14.69)	13.28% (12.86, 13.69)	
	**Total**	9.38% (0.82, 17.20)	3.24% (2.55, 3.93)	4.61% (4.14, 5.09)	6.06% (5.71, 6.42)	8.83% (8.35, 9.30)	14.12% (13.69, 14.56)	9.33% (9.11, 9.55)	9.38% (0.82, 17.20)

Legend: Observed 5-year risk (in %) for CVD and ASCVD

<1	1-2	2-3	3-4	4-5	5-6	6-7	7-8	8-9	9-10	10-11	11-12	12-13	13-14	14-15	15-16	16-17	17-18

Framingham 1991 general CVD – AGE 70 to 75

Reclassification table

Original equation	Re-estimated equation: <1.25%	1.25 – 2.49%	2.50 – 3.74%	3.75 - 4.99%	5.00 – 7.49%	7.50 - 9.99%	≥ 10.00%	Total
**<1.25%**	0 (0.0%)	0 (0.0%)	1 (0.0%)	8 (0.0%)	0 (0.0%)	0 (0.0%)	0 (0.0%)	9 (0.0%)
**1.25 – 2.49%**	0 (0.0%)	0 (0.0%)	0 (0.0%)	50 (0.1%)	207 (0.6%)	0 (0.0%)	0 (0.0%)	257 (0.7%)
**2.50 – 3.74%**	0 (0.0%)	0 (0.0%)	0 (0.0%)	0 (0.0%)	1,160 (3.3%)	21 (0.1%)	0 (0.0%)	1,181 (3.3%)
**3.75 - 4.99%**	0 (0.0%)	0 (0.0%)	0 (0.0%)	0 (0.0%)	1,702 (4.8%)	754 (2.1%)	11 (0.0%)	2,467 (7.0%)
**5.00 – 7.49%**	0 (0.0%)	0 (0.0%)	0 (0.0%)	0 (0.0%)	414 (1.2%)	5,167 (14.6%)	704 (2.0%)	6,285 (17.7%)
**7.50 - 9.99%**	0 (0.0%)	0 (0.0%)	0 (0.0%)	0 (0.0%)	0 (0.0%)	2,288 (6.4%)	3,503 (9.9%)	5,791 (16.3%)
**≥ 10.00%**	0 (0.0%)	0 (0.0%)	0 (0.0%)	0 (0.0%)	0 (0.0%)	132 (0.4%)	19,368 (54.6%)	19,500 (54.9%)
**Total**	0 (0.0%)	0 (0.0%)	1 (0.0%)	58 (0.2%)	3,483 (9.8%)	8,362 (23.6%)	23,586 (66.5%)	35,490 (100%)

Reclassification **NO: 62.2%** **UP: 37.4%** **DOWN: 0.4%**

Original equation	<1.25%	1.25 – 2.49%	2.50 – 3.74%	3.75 - 4.99%	5.00 – 7.49%	7.50 - 9.99%	≥ 10.00%	Total
**<1.25%**								
**1.25 – 2.49%**					6.36% (2.52, 10.06)			7.68% (4.01, 11.20)
**2.50 – 3.74%**					8.8% (6.84, 10.72)			8.66% (6.73, 10.55)
**3.75 - 4.99%**					9.22% (7.58, 10.83)	11.66% (8.80, 14.43)		9.97% (8.54, 11.37)
**5.00 – 7.49%**					10.18% (6.77, 13.46)	9.24% (8.29, 10.17)	16.05% (12.63, 19.33)	10.06% (9.17, 10.95)
**7.50 - 9.99%**						10.18% (8.67, 11.67)	12.51% (11.19, 13.81)	11.58% (10.59, 12.56)
**≥ 10.00%**						8.85% (2.83, 14.50)	17.15% (16.51, 17.79)	17.1% (16.46, 17.73)
**Total**					9.04% (7.91, 10.16)	9.68% (8.92, 10.44)	16.43% (15.86, 16.99)	14.1% (13.67, 14.54)

Legend: Observed 5-year risk (in %) for CVD and ASCVD

<1	1-2	2-3	3-4	4-5	5-6	6-7	7-8	8-9	9-10	10-11	11-12	12-13	13-14	14-15	15-16	16-17	17-18

Framingham 1991 general CVD – individuals without diabetes

Reclassification table

Original equation	Re-estimated equation: **<1.25%**	**1.25 – 2.49%**	**2.50 – 3.74%**	**3.75 - 4.99%**	**5.00 – 7.49%**	**7.50 - 9.99%**	**≥ 10.00%**	**Total**
**<1.25%**	149,421 (29.9%)	10,885 (2.2%)	279 (0.1%)	10 (0.0%)	0 (0.0%)	0 (0.0%)	0 (0.0%)	160,595 (32.1%)
**1.25 – 2.49%**	23,899 (4.8%)	43,645 (8.7%)	10,064 (2.0%)	1,635 (0.3%)	274 (0.1%)	0 (0.0%)	0 (0.0%)	79,517 (15.9%)
**2.50 – 3.74%**	2,249 (0.4%)	21,230 (4.2%)	20,417 (4.1%)	8,194 (1.6%)	3,631 (0.7%)	31 (0.0%)	0 (0.0%)	55,752 (11.1%)
**3.75 - 4.99%**	257 (0.1%)	6,037 (1.2%)	14,040 (2.8%)	11,781 (2.4%)	9,928 (2.0%)	993 (0.2%)	11 (0.0%)	43,047 (8.6%)
**5.00 – 7.49%**	22 (0.0%)	1,747 (0.3%)	8,819 (1.8%)	14,291 (2.9%)	24,133 (4.8%)	10,672 (2.1%)	1,009 (0.2%)	60,693 (12.1%)
**7.50 - 9.99%**	0 (0.0%)	86 (0.0%)	1,137 (0.2%)	3,825 (0.8%)	13,415 (2.7%)	12,835 (2.6%)	6,701 (1.3%)	37,999 (7.6%)
**≥ 10.00%**	0 (0.0%)	4 (0.0%)	117 (0.0%)	658 (0.1%)	6,068 (1.2%)	12,214 (2.4%)	43,796 (8.8%)	62,857 (12.6%)
**Total**	175,848 (35.1%)	83,634 (16.7%)	54,873 (11.0%)	40,394 (8.1%)	57,449 (11.5%)	36,745 (7.3%)	51,517 (10.3%)	500,460 (100%)

**Reclassification**	**NO: 61.1%**	**UP: 12.9%**	**DOWN: 26.0%**

5-year Kaplan Meier estimate of general CVD with 95% confidence intervals

Original equation	<1.25%	1.25 – 2.49%	2.50 – 3.74%	3.75 - 4.99%	5.00 – 7.49%	7.50 - 9.99%	≥ 10.00%	Total
**<1.25%**	0.5% (0.45, 0.55)	1.36% (1.09, 1.64)	4.16% (1.12, 7.10)					0.57% (0.52, 0.62)
**1.25 – 2.49%**	0.97% (0.81, 1.12)	1.68% (1.53, 1.84)	2.69% (2.29, 3.08)	5.36% (4.12, 6.59)	7.32% (3.64, 10.87)			1.70% (1.59, 1.82)
**2.50 – 3.74%**	2.04% (1.22, 2.86)	1.66% (1.44, 1.88)	2.50% (2.24, 2.77)	4.57% (4.02, 5.12)	6.72% (5.76, 7.68)			2.78% (2.61, 2.95)
**3.75 - 4.99%**	2.14% (0.00, 4.27)	2.12% (1.65, 2.59)	2.66% (2.32, 3.00)	3.78% (3.36, 4.20)	6.38% (5.80, 6.96)	12.91% (10.27, 15.46)		4.04% (3.81, 4.27)
**5.00 – 7.49%**		3.38% (2.28, 4.46)	3.10% (2.64, 3.56)	4.42% (3.99, 4.86)	5.81% (5.45, 6.16)	9.10% (8.45, 9.74)	14.65% (12.01, 17.20)	5.81% (5.58, 6.04)
**7.50 - 9.99%**			3.70% (2.34, 5.03)	4.34% (3.51, 5.17)	6.31% (5.79, 6.83)	9.04% (8.44, 9.64)	12.85% (11.90, 13.78)	8.21% (7.87, 8.55)
**≥ 10.00%**			10.23% (2.21, 17.60)	7.53% (4.88, 10.10)	7.38% (6.52, 8.23)	8.91% (8.28, 9.53)	14.13% (13.73, 14.52)	12.45% (12.14, 12.77)
**Total**	0.59% (0.54, 0.64)	1.70% (1.59, 1.82)	2.72% (2.55, 2.89)	4.34% (4.09, 4.59)	6.24% (6, 6.48)	9.13% (8.77, 9.49)	13.96% (13.60, 14.32)	4.11% (4.04, 4.18)

Legend: Observed 5-year risk (in %) for CVD and ASCVD

<1	1-2	2-3	3-4	4-5	5-6	6-7	7-8	8-9	9-10	10-11	11-12	12-13	13-14	14-15	15-16	16-17	17-18

Framingham 1991 general CVD – – individuals with diabetes

Reclassification table

Original equation	Re-estimated equation: **<1.25%**	**1.25 – 2.49%**	**2.50 – 3.74%**	**3.75 - 4.99%**	**5.00 – 7.49%**	**7.50 - 9.99%**	**≥ 10.00%**	**Total**
**<1.25%**	542 (2.2%)	20 (0.1%)	0 (0.0%)	0 (0.0%)	0 (0.0%)	0 (0.0%)	0 (0.0%)	562 (2.2%)
**1.25 – 2.49%**	398 (1.6%)	258 (1.0%)	37 (0.1%)	4 (0.0%)	0 (0.0%)	0 (0.0%)	0 (0.0%)	697 (2.8%)
**2.50 – 3.74%**	105 (0.4%)	352 (1.4%)	174 (0.7%)	65 (0.3%)	18 (0.1%)	0 (0.0%)	0 (0.0%)	714 (2.9%)
**3.75 - 4.99%**	25 (0.1%)	234 (0.9%)	324 (1.3%)	195 (0.8%)	110 (0.4%)	8 (0.0%)	0 (0.0%)	896 (3.6%)
**5.00 – 7.49%**	8 (0.0%)	155 (0.6%)	378 (1.5%)	505 (2.0%)	740 (3.0%)	272 (1.1%)	65 (0.3%)	2123 (8.5%)
**7.50 - 9.99%**	1 (0.0%)	27 (0.1%)	123 (0.5%)	281 (1.1%)	895 (3.6%)	802 (3.2%)	500 (2.0%)	2629 (10.5%)
**≥ 10.00%**	0 (0.0%)	5 (0.0%)	43 (0.2%)	139 (0.6%)	862 (3.4%)	1,825 (7.3%)	14,543 (58.1%)	17,417 (69.6%)
**Total**	1,079 (4.3%)	1,051 (4.2%)	1,079 (4.3%)	1,189 (4.7%)	2,625 (10.5%)	2,907 (11.6%)	15,108 (60.3%)	25,038 (100%)

**Reclassification**	**NO: 68.9%**	**UP: 4.4%**	**DOWN: 26.7%**

5-year Kaplan Meier estimate of general CVD with 95% confidence intervals

Original equation	<1.25%	1.25 – 2.49%	2.50 – 3.74%	3.75 - 4.99%	5.00 – 7.49%	7.50 - 9.99%	≥ 10.00%	Total
**<1.25%**	0.6% (0.00, 1.27)							0.58% (0.00, 1.23)
**1.25 – 2.49%**	0.53% (0.00, 1.27)	3.12% (0.32, 5.84)						2.09% (0.75, 3.41)
**2.50 – 3.74%**	3.45% (0.00, 9.87)	2.17% (0.18, 4.12)	5.62% (1.18, 9.86)					4.70% (2.55, 6.79)
**3.75 - 4.99%**		2.59% (0.23, 4.90)	2.48% (0.60, 4.32)	8.31% (3.71, 12.68)	11.57% (4.17, 18.39)			4.96% (3.29, 6.59)
**5.00 – 7.49%**		1.06% (0.00, 3.12)	3.24% (0.98, 5.45)	5.35% (2.81, 7.82)	7.54% (5.19, 9.82)	14.97% (9.77, 19.88)		7.46% (6.06, 8.83)
**7.50 - 9.99%**			4.74% (0.00, 9.58)	3.17% (0.74, 5.54)	6.56% (4.56, 8.52)	7.32% (5.16, 9.44)	13.19% (9.25, 16.95)	7.67% (6.39, 8.93)
**≥ 10.00%**				5.70% (0.82, 10.34)	7.27% (5.15, 9.33)	9.30% (7.70, 10.88)	18.17% (17.42, 18.91)	16.58% (15.92, 17.23)
**Total**	0.82% (0.05, 1.59)	2.52% (1.25, 3.77)	3.58% (2.21, 4.93)	5.93% (4.22, 7.62)	7.38% (6.16, 8.59)	9.31% (8.04, 10.56)	18.06% (17.32, 18.78)	13.44% (12.93, 13.95)

Legend: Observed 5-year risk (in %) for CVD and ASCVD

<1	1-2	2-3	3-4	4-5	5-6	6-7	7-8	8-9	9-10	10-11	11-12	12-13	13-14	14-15	15-16	16-17	17-18

Framingham 1991 general CVD – individuals without hypertension

Reclassification table

Original equation	Re-estimated equation: **<1.25%**	**1.25 – 2.49%**	**2.50 – 3.74%**	**3.75 - 4.99%**	**5.00 – 7.49%**	**7.50 - 9.99%**	**≥ 10.00%**	**Total**
**<1.25%**	135,560 (39.4%)	10571 (3.1%)	275 (0.1%)	10 (0.0%)	0 (0.0%)	0 (0.0%)	0 (0.0%)	146,416 (42.6%)
**1.25 – 2.49%**	14,959 (4.3%)	35,753 (10.4%)	9,412 (2.7%)	1,594 (0.5%)	273 (0.1%)	0 (0.0%)	0 (0.0%)	61,991 (18.0%)
**2.50 – 3.74%**	782 (0.2%)	12,080 (3.5%)	15,490 (4.5%)	7,123 (2.1%)	3,433 (1.0%)	31 (0.0%)	0 (0.0%)	38,939 (11.3%)
**3.75 - 4.99%**	31 (0.0%)	2,185 (0.6%)	7,622 (2.2%)	8,009 (2.3%)	7,991 (2.3%)	938 (0.3%)	10 (0.0%)	26,786 (7.8%)
**5.00 – 7.49%**	3 (0.0%)	327 (0.1%)	2,994 (0.9%)	6,481 (1.9%)	13,656 (4.0%)	7,423 (2.2%)	1,013 (0.3%)	31,897 (9.3%)
**7.50 - 9.99%**	0 (0.0%)	7 (0.0%)	155 (0.0%)	952 (0.3%)	4,834 (1.4%)	5,956 (1.7%)	4,993 (1.5%)	16,897 (4.9%)
**≥ 10.00%**	0 (0.0%)	0 (0.0%)	8 (0.0%)	64 (0.0%)	1,109 (0.3%)	3,028 (0.9%)	16,842 (4.9%)	21,051 (6.1%)
**Total**	15,1335 (44.0%)	60,923 (17.7%)	35,956 (10.5%)	24,233 (7.0%)	31,296 (9.1%)	17,376 (5.1%)	22,858 (6.6%)	343,977 (100%)

**Reclassification**	**NO: 67.2%**	**UP: 16.0%**	**DOWN: 16.8%**

5-year Kaplan Meier estimate of general CVD with 95% confidence intervals

Original equation	<1.25%	1.25 – 2.49%	2.50 – 3.74%	3.75 - 4.99%	5.00 – 7.49%	7.50 - 9.99%	≥ 10.00%	Total
**<1.25%**	0.47% (0.43, 0.52)	1.40% (1.12, 1.69)	3.76% (0.81, 6.62)					0.55% (0.50, 0.60)
**1.25 – 2.49%**	0.94% (0.74, 1.14)	1.69% (1.51, 1.86)	2.69% (2.28, 3.10)	5.46% (4.19, 6.71)	7.33% (3.64, 10.88)			1.80% (1.66, 1.93)
**2.50 – 3.74%**	2.77% (1.13, 4.39)	1.40% (1.14, 1.66)	2.55% (2.25, 2.85)	4.56% (3.96, 5.15)	6.66% (5.67, 7.63)			2.97% (2.76, 3.18)
**3.75 - 4.99%**		2.03% (1.24, 2.82)	2.69% (2.22, 3.15)	3.73% (3.23, 4.24)	6.40% (5.75, 7.05)	12.24% (9.59, 14.82)		4.44% (4.13, 4.75)
**5.00 – 7.49%**		2.60% (0.22, 4.92)	2.90% (2.16, 3.64)	4.79% (4.12, 5.45)	6.22% (5.74, 6.71)	9.20% (8.43, 9.97)	15.25% (12.56, 17.86)	6.63% (6.29, 6.96)
**7.50 - 9.99%**			5.81% (1.13, 10.26)	5.25% (3.47, 7.00)	6.92% (6, 7.83)	8.32% (7.49, 9.15)	13.13% (12.02, 14.23)	9.26% (8.73, 9.79)
**≥ 10.00%**					7.24% (5.30, 9.15)	9.27% (8.01, 10.51)	15.54% (14.88, 16.19)	14.26% (13.69, 14.83)
**Total**	0.53% (0.49, 0.58)	1.61% (1.48, 1.74)	2.67% (2.46, 2.88)	4.45% (4.13, 4.77)	6.46% (6.13, 6.79)	9.08% (8.57, 9.59)	15.00% (14.44, 15.55)	3.31% (3.23, 3.38)

Legend: Observed 5-year risk (in %) for CVD and ASCVD

<1	1-2	2-3	3-4	4-5	5-6	6-7	7-8	8-9	9-10	10-11	11-12	12-13	13-14	14-15	15-16	16-17	17-18

Framingham 1991 general CVD – individuals with hypertension

Reclassification table

Re-estimated equation (columns); Original equation (rows)

Original equation	<1.25%	1.25 – 2.49%	2.50 – 3.74%	3.75 - 4.99%	5.00 – 7.49%	7.50 - 9.99%	≥ 10.00%	Total
**<1.25%**	14,403 (7.9%)	334 (0.2%)	4 (0.0%)	0 (0.0%)	0 (0.0%)	0 (0.0%)	0 (0.0%)	14,741 (8.1%)
**1.25 – 2.49%**	9,338 (5.1%)	8,150 (4.5%)	689 (0.4%)	45 (0.0%)	1 (0.0%)	0 (0.0%)	0 (0.0%)	18,223 (10.0%)
**2.50 – 3.74%**	1,572 (0.9%)	9,502 (5.2%)	5,101 (2.8%)	1,136 (0.6%)	216 (0.1%)	0 (0.0%)	0 (0.0%)	17,527 (9.7%)
**3.75 - 4.99%**	251 (0.1%)	4,086 (2.3%)	6,742 (3.7%)	3,967 (2.2%)	2,047 (1.1%)	63 (0.0%)	1 (0.0%)	17,157 (9.5%)
**5.00 – 7.49%**	27 (0.0%)	1,575 (0.9%)	6,203 (3.4%)	8,315 (4.6%)	11,217 (6.2%)	3,521 (1.9%)	61 (0.0%)	30,919 (17.0%)
**7.50 - 9.99%**	1 (0.0%)	106 (0.1%)	1,105 (0.6%)	3,154 (1.7%)	9,476 (5.2%)	7,681 (4.2%)	2,208 (1.2%)	23,731 (13.1%)
**≥ 10.00%**	0 (0.0%)	9 (0.0%)	152 (0.1%)	733 (0.4%)	5,821 (3.2%)	11,011 (6.1%)	41,497 (22.9%)	59,223 (32.6%)
**Total**	25,592 (14.1%)	23,762 (13.1%)	19,996 (11.0%)	17,350 (9.6%)	28,778 (15.9%)	22,276 (12.3%)	43,767 (24.1%)	181,521 (100%)

**Reclassification**	**NO: 50.7%**	**UP: 5.7%**	**DOWN: 43.6%**

5-year Kaplan Meier estimate of general CVD with 95% confidence intervals

Original equation	<1.25%	1.25 – 2.49%	2.50 – 3.74%	3.75 - 4.99%	5.00 – 7.49%	7.50 - 9.99%	≥ 10.00%	Total
**<1.25%**	0.77% (0.58, 0.97)							0.76% (0.57, 0.96)
**1.25 – 2.49%**	0.99% (0.74, 1.23)	1.69% (1.35, 2.04)	2.83% (1.36, 4.29)					1.38% (1.18, 1.59)
**2.50 – 3.74%**	1.77% (0.80, 2.73)	2.03% (1.65, 2.41)	2.47% (1.94, 3)	5.23% (3.65, 6.77)	8.89% (4.29, 13.27)			2.44% (2.14, 2.73)
**3.75 - 4.99%**	2.19% (0.00, 4.36)	2.18% (1.62, 2.75)	2.62% (2.15, 3.08)	4.08% (3.33, 4.83)	6.58% (5.30, 7.85)			3.43% (3.10, 3.77)
**5.00 – 7.49%**		3.30% (2.17, 4.42)	3.22% (2.64, 3.78)	4.02% (3.64, 4.76)	5.41% (4.90, 5.92)	9.35% (8.18, 10.50)		5.07% (4.77, 5.38)
**7.50 - 9.99%**		3.06% (0.00, 6.43)	3.51% (2.16, 4.84)	3.96% (3.08, 4.83)	6.03% (5.43, 6.63)	9.41% (8.61, 10.21)	12.23% (10.58, 13.84)	7.40% (6.99, 7.81)
**≥ 10.00%**			7.45% (1.68, 12.89)	6.75% (4.37, 9.07)	7.38% (6.51, 8.24)	8.89% (8.23, 9.54)	14.98% (14.57, 15.40)	13.05% (12.72, 13.38)
**Total**	0.93% (0.77, 1.08)	2.00% (1.77, 2.22)	2.86% (2.57, 3.15)	4.30% (3.91, 4.68)	6.10% (5.76, 6.44)	9.20% (8.73, 9.67)	14.85% (14.45, 15.25)	6.97% (6.82, 7.11)

Legend: Observed 5-year risk (in %) for CVD and ASCVD

<1	1-2	2-3	3-4	4-5	5-6	6-7	7-8	8-9	9-10	10-11	11-12	12-13	13-14	14-15	15-16	16-17	17-18

Framingham 2008 general CVD - MEN

Reclassification table

Re-estimated equation (columns); Original equation (rows)

Original equation	<1.25%	1.25 – 2.49%	2.50 – 3.74%	3.75 - 4.99%	5.00 – 7.49%	7.50 - 9.99%	≥ 10.00%	Total
**<1.25%**	20,453 (8.3%)	48 (0.0%)	0 (0.0%)	0 (0.0%)	0 (0.0%)	0 (0.0%)	0 (0.0%)	20,501 (8.3%)
**1.25 – 2.49%**	27,595 (11.2%)	10,226 (4.2%)	287 (0.1%)	9 (0.0%)	0 (0.0%)	0 (0.0%)	0 (0.0%)	38,117 (15.5%)
**2.50 – 3.74%**	4,825 (2.0%)	19,887 (8.1%)	5447 (2.2%)	575 (0.2%)	78 (0.0%)	2 (0.0%)	0 (0.0%)	30,814 (12.5%)
**3.75 - 4.99%**	449 (0.2%)	9,033 (3.7%)	10,495 (4.3%)	3,452 (1.4%)	1,080 (0.4%)	69 (0.0%)	2 (0.0%)	24,580 (10.0%)
**5.00 – 7.49%**	45 (0.0%)	3,001 (1.2%)	10,789 (4.4%)	10,836 (4.4%)	9,425 (3.8%)	2,256 (0.9%)	360 (0.1%)	36,712 (14.9%)
**7.50 - 9.99%**	0 (0.0%)	137 (0.1%)	1,788 (0.7%)	4,613 (1.9%)	10,482 (4.3%)	6,142 (2.5%)	3,273 (1.3%)	26,435 (10.7%)
**≥ 10.00%**	0 (0.0%)	15 (0.0%)	219 (0.1%)	1,232 (0.5%)	7,565 (3.1%)	11,553 (4.7%)	48,196 (19.6%)	68,780 (28.0%)
**Total**	53,367 (21.7%)	42,347 (17.2%)	29,025 (11.8%)	20,717 (8.4%)	28,630 (11.6%)	20,022 (8.1%)	51,831 (21.1%)	245,939 (100%)

Reclassification **NO: 42.0%** **UP: 3.3%** **DOWN: 54.7%**

5-year Kaplan Meier estimate of general CVD with 95% confidence intervals

Original equation	<1.25%	1.25 – 2.49%	2.50 – 3.74%	3.75 - 4.99%	5.00 – 7.49%	7.50 - 9.99%	≥ 10.00%	Total
**<1.25%**	0.31% (0.21, 0.40)							0.31% (0.21, 0.40)
**1.25 – 2.49%**	0.62% (0.49, 0.74)	1.29% (1, 1.57)	2.42% (0.17, 4.62)					0.82% (0.70, 0.94)
**2.50 – 3.74%**	0.90% (0.56, 1.23)	1.5% (1.27, 1.72)	2.31% (1.83, 2.78)	5.2% (3.12, 7.24)				1.65% (1.47, 1.83)
**3.75 - 4.99%**	1.98% (0.36, 3.57)	1.62% (1.29, 1.95)	2.69% (2.30, 3.09)	4.1% (3.28, 4.90)	5.27% (3.71, 6.81)			2.67% (2.42, 2.93)
**5.00 – 7.49%**		2.64% (1.88, 3.40)	2.79% (2.39, 3.18)	4.23% (3.74, 4.71)	5.90% (5.31, 6.48)	11.48% (9.86, 13.07)	15.19% (10.60, 19.55)	4.73% (4.46, 5.01)
**7.50 - 9.99%**		4.66% (0.94, 8.24)	3.19% (2.18, 4.20)	4.78% (3.99, 5.55)	6.18% (5.60, 6.76)	9.47% (8.58, 10.34)	14.44% (13.01, 15.85)	7.64% (7.24, 8.03)
**≥ 10.00%**			8.53% (3.56, 13.24)	6.02% (4.33, 7.68)	7.95% (7.15, 8.74)	8.65% (8.02, 9.27)	15.79% (15.39, 16.17)	13.62% (13.31, 13.93)
**Total**	0.54% (0.46, 0.62)	1.56% (1.41, 1.72)	2.72% (2.48, 2.95)	4.46% (4.11, 4.81)	6.52% (6.16, 6.87)	9.30% (8.81, 9.79)	15.69% (15.32, 16.07)	6.16% (6.04, 6.28)

Legend: Observed 5-year risk (in %) for CVD and ASCVD

<1	1-2	2-3	3-4	4-5	5-6	6-7	7-8	8-9	9-10	10-11	11-12	12-13	13-14	14-15	15-16	16-17	17-18

Framingham 2008 general CVD - WOMEN

Reclassification table

Original equation	Re-estimated equation: <1.25%	1.25 – 2.49%	2.50 – 3.74%	3.75 - 4.99%	5.00 – 7.49%	7.50 - 9.99%	≥ 10.00%	Total
**<1.25%**	84,409 (29.4%)	1,873 (0.7%)	34 (0.0%)	1 (0.0%)	1 (0.0%)	0 (0.0%)	0 (0.0%)	86,318 (30.1%)
**1.25 – 2.49%**	32,731 (11.4%)	25,848 (9.0%)	3,772 (1.3%)	734 (0.3%)	221 (0.1%)	19 (0.0%)	0 (0.0%)	63,325 (22.1%)
**2.50 – 3.74%**	3,038 (1.1%)	17,162 (6.0%)	11,453 (4.0%)	4,451 (1.6%)	2,507 (0.9%)	272 (0.1%)	24 (0.0%)	38,907 (13.6%)
**3.75 - 4.99%**	363 (0.1%)	4,851 (1.7%)	8,143 (2.8%)	6,282 (2.2%)	5,918 (2.1%)	1,346 (0.5%)	172 (0.1%)	27,075 (9.4%)
**5.00 – 7.49%**	54 (0.0%)	1,631 (0.6%)	5,230 (1.8%)	6,913 (2.4%)	11,733 (4.1%)	5,830 (2.0%)	1,876 (0.7%)	33,267 (11.6%)
**7.50 - 9.99%**	4 (0.0%)	126 (0.0%)	899 (0.3%)	1,951 (0.7%)	5,255 (1.8%)	4,947 (1.7%)	3,614 (1.3%)	16,796 (5.9%)
**≥ 10.00%**	0 (0.0%)	18 (0.0%)	171 (0.1%)	595 (0.2%)	2,862 (1.0%)	4,663 (1.6%)	12,732 (4.4%)	21,041 (7.3%)
**Total**	120,599 (42.1%)	51,509 (18.0%)	29,702 (10.4%)	20,927 (7.3%)	28,497 (9.9%)	17,077 (6.0%)	18,418 (6.4%)	286,729 (100%)

Reclassification **NO: 54.9%** **UP: 11.4%** **DOWN: 33.7%**

5-year Kaplan Meier estimate of general CVD with 95% confidence intervals

Original equation	<1.25%	1.25 – 2.49%	2.50 – 3.74%	3.75 - 4.99%	5.00 – 7.49%	7.50 - 9.99%	≥ 10.00%	Total
**<1.25%**	0.43% (0.38, 0.49)	1.32% (0.65, 1.98)						0.46% (0.40, 0.52)
**1.25 – 2.49%**	0.89% (0.76, 1.02)	1.51% (1.32, 1.69)	3.02% (2.34, 3.69)	5.17% (3.36, 6.94)	11.97% (6.83, 16.82)			1.38% (1.27, 1.49)
**2.50 – 3.74%**	1.25% (0.74, 1.76)	1.86% (1.60, 2.12)	2.71% (2.33, 3.08)	4.77% (4.01, 5.52)	6.82% (5.63, 7.98)	14.42% (8.89, 19.61)		2.85% (2.64, 3.06)
**3.75 - 4.99%**	4.88% (1.56, 8.08)	2.55% (1.99, 3.10)	2.88% (2.43, 3.33)	4.17% (3.58, 4.76)	5.99% (5.27, 6.70)	9.43% (7.56, 11.26)	17.73% (10.54, 24.33)	4.28% (3.99, 4.57)
**5.00 – 7.49%**		2.85% (1.78, 3.91)	2.91% (2.34, 3.48)	4.19% (3.61, 4.77)	5.84% (5.33, 6.35)	9.31% (8.41, 10.20)	11.54% (9.86, 13.19)	5.87% (5.56, 6.17)
**7.50 - 9.99%**		3.09% (0.00, 6.52)	2.89% (1.56, 4.21)	5.7% (4.38, 7.01)	6.69% (5.86, 7.52)	8.64% (7.67, 9.60)	11.17% (9.95, 12.38)	7.93% (7.43, 8.44)
**≥ 10.00%**			5.09% (0.76, 9.22)	5.46% (3.13, 7.74)	6.64% (5.53, 7.74)	8.30% (7.37, 9.22)	14.00% (13.26, 14.72)	11.45% (10.93, 11.97)
**Total**	0.60% (0.54, 0.66)	1.76% (1.61, 1.90)	2.85% (2.62, 3.09)	4.51% (4.17, 4.86)	6.24% (5.90, 6.58)	8.97% (8.45, 9.48)	13.23% (12.64, 13.82)	3.32% (3.24, 3.40)

Legend: Observed 5-year risk (in %) for CVD and ASCVD

<1	1-2	2-3	3-4	4-5	5-6	6-7	7-8	8-9	9-10	10-11	11-12	12-13	13-14	14-15	15-16	16-17	17-18

Framingham 2008 general CVD – AGE 30 to 39

Reclassification table

Re-estimated equation

Original equation	<1.25%	1.25 – 2.49%	2.50 – 3.74%	3.75 - 4.99%	5.00 – 7.49%	7.50 - 9.99%	≥ 10.00%	Total
**<1.25%**	75,548 (62.3%)	0 (0.0%)	0 (0.0%)	0 (0.0%)	0 (0.0%)	0 (0.0%)	0 (0.0%)	75,548 (62.3%)
**1.25 – 2.49%**	30,807 (25.4%)	515 (0.4%)	0 (0.0%)	0 (0.0%)	0 (0.0%)	0 (0.0%)	0 (0.0%)	31,322 (25.8%)
**2.50 – 3.74%**	5,577 (4.6%)	3,193 (2.6%)	0 (0.0%)	0 (0.0%)	0 (0.0%)	0 (0.0%)	0 (0.0%)	8,770 (7.2%)
**3.75 - 4.99%**	618 (0.5%)	2,550 (2.1%)	3 (0.0%)	0 (0.0%)	0 (0.0%)	0 (0.0%)	0 (0.0%)	3,171 (2.6%)
**5.00 – 7.49%**	75 (0.1%)	1,698 (1.4%)	145 (0.1%)	0 (0.0%)	0 (0.0%)	0 (0.0%)	0 (0.0%)	1,918 (1.6%)
**7.50 - 9.99%**	4 (0.0%)	131 (0.1%)	203 (0.2%)	4 (0.0%)	0 (0.0%)	0 (0.0%)	0 (0.0%)	342 (0.3%)
**≥ 10.00%**	0 (0.0%)	19 (0.0%)	96 (0.1%)	30 (0.0%)	6 (0.0%)	0 (0.0%)	0 (0.0%)	151 (0.1%)
**Total**	112,629 (92.9%)	8,106 (6.7%)	447 (0.4%)	34 (0.0%)	6 (0.0%)	0 (0.0%)	0 (0.0%)	121,222 (100%)

**Reclassification**	**NO: 62.7%**	**UP: 0.0%**	**DOWN: 37.3%**

5-year Kaplan Meier estimate of general CVD with 95% confidence intervals

Original equation	<1.25%	1.25 – 2.49%	2.50 – 3.74%	3.75 - 4.99%	5.00 – 7.49%	7.50 - 9.99%	≥ 10.00%	Total
**<1.25%**	0.32% (0.27, 0.38)							0.32% (0.27, 0.38)
**1.25 – 2.49%**	0.71% (0.58, 0.83)	1.11% (0.01, 2.20)						0.71% (0.59, 0.84)
**2.50 – 3.74%**	1.00% (0.67, 1.32)	1.18% (0.66, 1.69)						1.06% (0.78, 1.34)
**3.75 - 4.99%**	3.4% (1.22, 5.54)	1.62% (0.93, 2.30)						1.95% (1.26, 2.64)
**5.00 – 7.49%**		2.45% (1.48, 3.41)	1.60% (0.00, 3.78)					2.30% (1.42, 3.17)
**7.50 - 9.99%**		4.86% (0.98, 8.59)	3.79% (0.70, 6.79)					4.10% (1.75, 6.39)
**≥ 10.00%**								5.10% (0.32, 9.65)
**Total**	0.48% (0.43, 0.54)	1.66% (1.28, 2.03)	3.53% (1.47, 5.55)					0.58% (0.52, 0.63)

Legend: Observed 5-year risk (in %) for CVD and ASCVD

<1	1-2	2-3	3-4	4-5	5-6	6-7	7-8	8-9	9-10	10-11	11-12	12-13	13-14	14-15	15-16	16-17	17-18

Framingham 2008 general CVD – AGE 40 to 49

Reclassification table

Original equation	Re-estimated equation: **<1.25%**	**1.25 – 2.49%**	**2.50 – 3.74%**	**3.75 - 4.99%**	**5.00 – 7.49%**	**7.50 - 9.99%**	**≥ 10.00%**	**Total**
**<1.25%**	28,088 (19.0%)	594 (0.4%)	0 (0.0%)	0 (0.0%)	0 (0.0%)	0 (0.0%)	0 (0.0%)	28,682 (19.4%)
**1.25 – 2.49%**	28,200 (19.1%)	19,204 (13.0%)	118 (0.1%)	0 (0.0%)	0 (0.0%)	0 (0.0%)	0 (0.0%)	47,522 (32.1%)
**2.50 – 3.74%**	2,264 (1.5%)	24,304 (16.4%)	2,174 (1.5%)	9 (0.0%)	0 (0.0%)	0 (0.0%)	0 (0.0%)	28,751 (19.4%)
**3.75 - 4.99%**	194 (0.1%)	9,197 (6.2%)	6,918 (4.7%)	205 (0.1%)	0 (0.0%)	0 (0.0%)	0 (0.0%)	16,514 (11.2%)
**5.00 – 7.49%**	24 (0.0%)	2,414 (1.6%)	9,879 (6.7%)	2,768 (1.9%)	114 (0.1%)	0 (0.0%)	0 (0.0%)	15,199 (10.3%)
**7.50 - 9.99%**	0 (0.0%)	110 (0.1%)	1,892 (1.3%)	3,128 (2.1%)	1,062 (0.7%)	1 (0.0%)	0 (0.0%)	6,193 (4.2%)
**≥ 10.00%**	0 (0.0%)	14 (0.0%)	225 (0.2%)	1,246 (0.8%)	2,934 (2.0%)	544 (0.4%)	174 (0.1%)	5,137 (3.5%)
**Total**	58,770 (39.7%)	55,837 (37.7%)	21,206 (14.3%)	7,356 (5.0%)	4,110 (2.8%)	545 (0.4%)	174 (0.1%)	147,998 (100%)

**Reclassification**	**NO: 33.8%**	**UP: 0.5%**	**DOWN: 65.7%**

5-year Kaplan Meier estimate of general CVD with 95% confidence intervals

Original equation	<1.25%	1.25 – 2.49%	2.50 – 3.74%	3.75 - 4.99%	5.00 – 7.49%	7.50 - 9.99%	≥ 10.00%	Total
**<1.25%**	0.58% (0.46, 0.69)	1.64% (0.25, 3.00)						0.60% (0.49, 0.72)
**1.25 – 2.49%**	0.83% (0.70, 0.96)	1.29% (1.09, 1.50)	4.35% (0.00, 9.72)					1.03% (0.91, 1.14)
**2.50 – 3.74%**	1.15% (0.57, 1.73)	1.52% (1.32, 1.72)	2.21% (1.50, 2.92)					1.55% (1.37, 1.73)
**3.75 - 4.99%**	3.71% (0.00, 7.51)	1.93% (1.58, 2.27)	2.86% (2.37, 3.34)	2.52% (0.00, 5.06)				2.34% (2.06, 2.63)
**5.00 – 7.49%**		2.80% (1.93, 3.67)	2.78% (2.37, 3.19)	4.24% (3.26, 5.21)	4.30% (0.00, 9.09)			3.07% (2.71, 3.42)
**7.50 - 9.99%**		3.37% (0.00, 7.07)	3.29% (2.28, 4.29)	4.90% (3.95, 5.83)	6.46% (4.60, 8.29)			4.65% (3.99, 5.30)
**≥ 10.00%**			8.25% (3.51, 12.76)	6.19% (4.46, 7.88)	8.23% (6.93, 9.51)	9.27% (6.36, 12.09)	18.10% (9.93, 25.52)	8.20% (7.23, 9.16)
**Total**	0.73% (0.65, 0.82)	1.57% (1.43, 1.70)	2.86% (2.57, 3.14)	4.84% (4.21, 5.45)	7.68% (6.62, 8.72)	9.26% (6.35, 12.07)	18.10% (9.93, 25.52)	1.79% (1.71, 1.88)

Legend: Observed 5-year risk (in %) for CVD and ASCVD

<1	1-2	2-3	3-4	4-5	5-6	6-7	7-8	8-9	9-10	10-11	11-12	12-13	13-14	14-15	15-16	16-17	17-18

Framingham 2008 general CVD – AGE 50 to 59

Reclassification table

Original equation	Re-estimated equation: **<1.25%**	**1.25 – 2.49%**	**2.50 – 3.74%**	**3.75 - 4.99%**	**5.00 – 7.49%**	**7.50 - 9.99%**	**≥ 10.00%**	**Total**
**<1.25%**	1,226 (0.9%)	1,299 (1.0%)	8 (0.0%)	0 (0.0%)	0 (0.0%)	0 (0.0%)	0 (0.0%)	2,533 (2.0%)
**1.25 – 2.49%**	1,319 (1.0%)	16,070 (12.4%)	2,337 (1.8%)	112 (0.1%)	5 (0.0%)	0 (0.0%)	0 (0.0%)	19,843 (15.3%)
**2.50 – 3.74%**	22 (0.0%)	9,504 (7.3%)	11,672 (9.0%)	1,516 (1.2%)	140 (0.1%)	3 (0.0%)	0 (0.0%)	22,857 (17.7%)
**3.75 - 4.99%**	0 (0.0%)	2,136 (1.7%)	10,590 (8.2%)	5,562 (4.3%)	837 (0.6%)	13 (0.0%)	0 (0.0%)	19,138 (14.8%)
**5.00 – 7.49%**	0 (0.0%)	520 (0.4%)	5,707 (4.4%)	12,183 (9.4%)	8,208 (6.3%)	208 (0.2%)	3 (0.0%)	26,829 (20.7%)
**7.50 - 9.99%**	0 (0.0%)	22 (0.0%)	581 (0.4%)	3,064 (2.4%)	10,074 (7.8%)	1,524 (1.2%)	53 (0.0%)	15,318 (11.8%)
**≥ 10.00%**	0 (0.0%)	0 (0.0%)	68 (0.1%)	507 (0.4%)	6,194 (4.8%)	8,330 (6.4%)	7,774 (6.0%)	22,873 (17.7%)
**Total**	2,567 (2.0%)	29,551 (22.8%)	30,963 (23.9%)	22,944 (17.7%)	25,458 (19.7%)	10,078 (7.8%)	7,830 (6.1%)	129,391 (100%)

Reclassification **NO: 40.2%** **UP: 5.1%** **DOWN: 54.7%**

5-year Kaplan Meier estimate of general CVD with 95% confidence intervals

Original equation	<1.25%	1.25 – 2.49%	2.50 – 3.74%	3.75 - 4.99%	5.00 – 7.49%	7.50 - 9.99%	≥ 10.00%	Total
**<1.25%**	1.48% (0.59, 2.36)	1.15% (0.43, 1.87)						1.30% (0.74, 1.86)
**1.25 – 2.49%**	0.92% (0.26, 1.57)	1.60% (1.35, 1.85)	2.79% (1.98, 3.60)	4.07% (0.05, 7.92)				1.72% (1.49, 1.95)
**2.50 – 3.74%**		2.19% (1.81, 2.58)	2.46% (2.11, 2.81)	4.63% (3.40, 5.85)	6.51% (1.37, 11.39)			2.52% (2.26, 2.77)
**3.75 - 4.99%**		2.41% (1.61, 3.21)	2.66% (2.26, 3.05)	4.03% (3.39, 4.66)	5.23% (3.39, 7.04)			3.14% (2.83, 3.45)
**5.00 – 7.49%**		3.18% (1.09, 5.22)	3.00% (2.43, 3.56)	4.22% (3.77, 4.67)	5.81% (5.18, 6.44)	11.38% (5.66, 16.76)		4.49% (4.18, 4.80)
**7.50 - 9.99%**			1.96% (0.65, 3.25)	5.38% (4.34, 6.41)	6.38% (5.77, 6.99)	10.12% (8.24, 11.96)		6.40% (5.90, 6.89)
**≥ 10.00%**				5.95% (3.36, 8.48)	7.60% (6.75, 8.44)	9.16% (8.40, 9.91)	14.07% (13.12, 15.01)	10.34% (9.85, 10.82)
**Total**	1.18% (0.64, 1.72)	1.86% (1.66, 2.05)	2.64% (2.42, 2.87)	4.39% (4.06, 4.71)	6.45% (6.07, 6.83)	9.34% (8.64, 10.03)	14.01% (13.07, 14.95)	4.71% (4.57, 4.86)

Legend: Observed 5-year risk (in %) for CVD and ASCVD

<1	1-2	2-3	3-4	4-5	5-6	6-7	7-8	8-9	9-10	10-11	11-12	12-13	13-14	14-15	15-16	16-17	17-18

Framingham 2008 general CVD – AGE 60 to 69

Reclassification table

		Re-estimated equation							
		**<1.25%**	**1.25 – 2.49%**	**2.50 – 3.74%**	**3.75 - 4.99%**	**5.00 – 7.49%**	**7.50 - 9.99%**	**≥ 10.00%**	**Total**
**Original equation**	**<1.25%**	0 (0.0%)	28 (0.0%)	26 (0.0%)	0 (0.0%)	0 (0.0%)	0 (0.0%)	0 (0.0%)	54 (0.1%)
	**1.25 – 2.49%**	0 (0.0%)	285 (0.3%)	1,603 (1.6%)	578 (0.6%)	120 (0.1%)	3 (0.0%)	0 (0.0%)	2,589 (2.7%)
	**2.50 – 3.74%**	0 (0.0%)	48 (0.0%)	3,054 (3.1%)	3,411 (3.5%)	1,619 (1.7%)	69 (0.1%)	3 (0.0%)	8,204 (8.4%)
	**3.75 - 4.99%**	0 (0.0%)	1 (0.0%)	1,127 (1.2%)	3,946 (4.1%)	4,821 (5.0%)	504 (0.5%)	39 (0.0%)	10,438 (10.7%)
	**5.00 – 7.49%**	0 (0.0%)	0 (0.0%)	288 (0.3%)	2,797 (2.9%)	11,475 (11.8%)	4,689 (4.8%)	620 (0.6%)	19,869 (20.4%)
	**7.50 - 9.99%**	0 (0.0%)	0 (0.0%)	11 (0.0%)	368 (0.4%)	4,366 (4.5%)	7,894 (8.1%)	3,049 (3.1%)	15,688 (16.1%)
	**≥ 10.00%**	0 (0.0%)	0 (0.0%)	1 (0.0%)	44 (0.0%)	1,273 (1.3%)	6,649 (6.8%)	32,385 (33.3%)	40,352 (41.5%)
	**Total**	0 (0.0%)	362 (0.4%)	6,110 (6.3%)	11,144 (11.5%)	23,674 (24.4%)	19,808 (20.4%)	36,096 (37.1%)	97,194 (100%)

**Reclassification**	**NO: 60.7%**	**UP: 21.8%**	**DOWN: 17.5%**

5-year Kaplan Meier estimate of general CVD with 95% confidence intervals

**Original equation**	**<1.25%**								
	**1.25 – 2.49%**		3.41% (0.57, 6.17)	3.12% (2.08, 4.15)	4.98% (2.96, 6.96)	12.10% (5.21, 18.48)			4.18% (3.25, 5.10)
	**2.50 – 3.74%**			3.27% (2.45, 4.09)	4.77% (3.89, 5.63)	6.62% (5.17, 8.05)			4.68% (4.12, 5.24)
	**3.75 - 4.99%**			3.37% (2.15, 4.58)	4.38% (3.63, 5.13)	5.59% (4.83, 6.34)	10.54% (7.29, 13.68)		5.16% (4.66, 5.65)
	**5.00 – 7.49%**			1.72% (0.00, 3.46)	4.15% (3.22, 5.08)	5.89% (5.37, 6.41)	9.70% (8.68, 10.72)	11.95% (8.95, 14.85)	6.70% (6.28, 7.12)
	**7.50 - 9.99%**				3.91% (1.37, 6.39)	5.93% (5.09, 6.76)	8.94% (8.18, 9.69)	12.92% (11.50, 14.31)	8.81% (8.28, 9.34)
	**≥ 10.00%**					6.06% (4.46, 7.63)	7.69% (6.92, 8.46)	14.6% (14.14, 15.05)	13.21% (12.81, 13.60)
	**Total**		3.17% (0.74, 5.55)	3.22% (2.66, 3.77)	4.44% (3.98, 4.90)	5.93% (5.57, 6.29)	8.81% (8.33, 9.28)	14.4% (13.97, 14.83)	9.34% (9.12, 9.56)

Legend: Observed 5-year risk (in %) for CVD and ASCVD

<1	1-2	2-3	3-4	4-5	5-6	6-7	7-8	8-9	9-10	10-11	11-12	12-13	13-14	14-15	15-16	16-17	17-18

Framingham 2008 general CVD – AGE 70 to 75

Reclassification table

Original equation	Re-estimated equation:	<1.25%	1.25 – 2.49%	2.50 – 3.74%	3.75 - 4.99%	5.00 – 7.49%	7.50 - 9.99%	≥ 10.00%	Total
	**<1.25%**	0 (0.0%)	0 (0.0%)	0 (0.0%)	1 (0.0%)	1 (0.0%)	0 (0.0%)	0 (0.0%)	2 (0.0%)
	**1.25 – 2.49%**	0 (0.0%)	0 (0.0%)	1 (0.0%)	53 (0.1%)	96 (0.3%)	16 (0.0%)	0 (0.0%)	166 (0.5%)
	**2.50 – 3.74%**	0 (0.0%)	0 (0.0%)	0 (0.0%)	90 (0.2%)	826 (2.2%)	202 (0.5%)	21 (0.1%)	1,139 (3.1%)
	**3.75 - 4.99%**	0 (0.0%)	0 (0.0%)	0 (0.0%)	21 (0.1%)	1,340 (3.6%)	898 (2.4%)	135 (0.4%)	2,394 (6.5%)
	**5.00 – 7.49%**	0 (0.0%)	0 (0.0%)	0 (0.0%)	1 (0.0%)	1,361 (3.7%)	3,189 (8.7%)	1,613 (4.4%)	6,164 (16.7%)
	**7.50 - 9.99%**	0 (0.0%)	0 (0.0%)	0 (0.0%)	0 (0.0%)	235 (0.6%)	1,670 (4.5%)	3,785 (10.3%)	5,690 (15.4%)
	**≥ 10.00%**	0 (0.0%)	0 (0.0%)	0 (0.0%)	0 (0.0%)	20 (0.1%)	693 (1.9%)	20,595 (55.9%)	21,308 (57.8%)
	**Total**	0 (0.0%)	0 (0.0%)	1 (0.0%)	166 (0.5%)	3,879 (10.5%)	6,668 (18.1%)	26,149 (70.9%)	36,863 (100%)

**Reclassification**	**NO: 64.1%**	**UP: 33.3%**	**DOWN: 2.6%**

5-year Kaplan Meier estimate of general CVD with 95% confidence intervals

Original equation		<1.25%	1.25 – 2.49%	2.50 – 3.74%	3.75 - 4.99%	5.00 – 7.49%	7.50 - 9.99%	≥ 10.00%	Total
	**<1.25%**								
	**1.25 – 2.49%**								10.57% (4.98, 15.83)
	**2.50 – 3.74%**					7.81% (5.58, 9.99)	13.53% (6.95, 19.65)		9.22% (7.09, 11.31)
	**3.75 - 4.99%**					7.38% (5.68, 9.05)	9.99% (7.60, 12.33)	20.14% (11.29, 28.12)	9.04% (7.65, 10.41)
	**5.00 – 7.49%**					6.07% (4.57, 7.54)	10.18% (8.90, 11.44)	12.21% (10.32, 14.06)	9.81% (8.92, 10.69)
	**7.50 - 9.99%**					13.35% (7.45, 18.87)	9.03% (7.24, 10.78)	12.66% (11.40, 13.90)	11.62% (10.60, 12.62)
	**≥ 10.00%**						8.84% (6.34, 11.27)	17.17% (16.55, 17.79)	16.90% (16.29, 17.51)
	**Total**				7.34% (3.03, 11.47)	7.44% (6.44, 8.43)	9.83% (8.95, 10.71)	16.23% (15.69, 16.76)	14.13% (13.70, 14.56)

Legend: Observed 5-year risk (in %) for CVD and ASCVD

<1	1-2	2-3	3-4	4-5	5-6	6-7	7-8	8-9	9-10	10-11	11-12	12-13	13-14	14-15	15-16	16-17	17-18

Framingham 2008 general CVD – individuals without diabetes

Reclassification table

Original equation	Re-estimated equation: <1.25%	1.25 – 2.49%	2.50 – 3.74%	3.75 - 4.99%	5.00 – 7.49%	7.50 - 9.99%	≥ 10.00%	Total
**<1.25%**	104,573 (20.6%)	1,921 (0.4%)	34 (0.0%)	1 (0.0%)	1 (0.0%)	0 (0.0%)	0 (0.0%)	106,530 (21.0%)
**1.25 – 2.49%**	59,743 (11.8%)	35,943 (7.1%)	4,048 (0.8%)	740 (0.1%)	220 (0.0%)	19 (0.0%)	0 (0.0%)	100,713 (19.9%)
**2.50 – 3.74%**	7,620 (1.5%)	36,630 (7.2%)	16,788 (3.3%)	4,991 (1.0%)	2,564 (0.5%)	270 (0.1%)	23 (0.0%)	68,886 (13.6%)
**3.75 - 4.99%**	760 (0.1%)	13,503 (2.7%)	18,345 (3.6%)	9,585 (1.9%)	6,892 (1.4%)	1,391 (0.3%)	165 (0.0%)	50,641 (10.0%)
**5.00 – 7.49%**	86 (0.0%)	4,365 (0.9%)	15,435 (3.0%)	17,238 (3.4%)	20,524 (4.0%)	7,837 (1.5%)	2,101 (0.4%)	67,586 (13.3%)
**7.50 - 9.99%**	2 (0.0%)	219 (0.0%)	2,486 (0.5%)	6,192 (1.2%)	14,890 (2.9%)	10,505 (2.1%)	6,356 (1.3%)	40,650 (8.0%)
**≥ 10.00%**	0 (0.0%)	20 (0.0%)	316 (0.1%)	1,573 (0.3%)	9,199 (1.8%)	14,270 (2.8%)	46,658 (9.2%)	72,036 (14.2%)
**Total**	172,784 (34.1%)	92,601 (18.3%)	57,452 (11.3%)	40,320 (8.0%)	54,290 (10.7%)	34,292 (6.8%)	55,303 (10.9%)	507,042 (100%)

**Reclassification**	**NO: 48.2%**	**UP: 7.8%**	**DOWN: 44.0%**

5-year Kaplan Meier estimate of general CVD with 95% confidence intervals

Original equation	<1.25%	1.25 – 2.49%	2.50 – 3.74%	3.75 - 4.99%	5.00 – 7.49%	7.50 - 9.99%	≥ 10.00%	Total
**<1.25%**	0.41% (0.36, 0.46)	1.29% (0.64, 1.94)						0.43% (0.38, 0.48)
**1.25 – 2.49%**	0.77% (0.68, 0.86)	1.45% (1.29, 1.60)	2.98% (2.33, 3.62)	5.12% (3.33, 6.89)	11.51% (6.44, 16.31)			1.18% (1.09, 1.26)
**2.50 – 3.74%**	1.01% (0.73, 1.29)	1.65% (1.48, 1.82)	2.57% (2.27, 2.87)	4.77% (4.06, 5.48)	7.01% (5.81, 8.18)	13.66% (8.21, 18.78)		2.32% (2.17, 2.46)
**3.75 - 4.99%**	3.75% (1.70, 5.75)	1.94% (1.65, 2.23)	2.67% (2.38, 2.96)	4.11% (3.63, 4.59)	5.8% (5.14, 6.44)	9.86% (7.97, 11.72)	17.87% (10.42, 24.71)	3.47% (3.28, 3.67)
**5.00 – 7.49%**		2.67% (2.04, 3.29)	2.83% (2.49, 3.16)	4.23% (3.85, 4.61)	5.82% (5.43, 6.21)	9.93% (9.13, 10.72)	11.58% (9.99, 13.14)	5.25% (5.04, 5.46)
**7.50 - 9.99%**		4.7% (1.60, 7.70)	3.04% (2.21, 3.86)	4.99% (4.30, 5.68)	6.36% (5.87, 6.85)	8.99% (8.32, 9.65)	12.72% (11.74, 13.68)	7.71% (7.39, 8.03)
**≥ 10.00%**			7.92% (3.94, 11.74)	5.48% (4.09, 6.85)	7.38% (6.70, 8.06)	8.47% (7.91, 9.02)	14.41% (14.02, 14.79)	12.20% (11.90, 12.49)
**Total**	0.58% (0.53, 0.63)	1.66% (1.55, 1.77)	2.75% (2.58, 2.92)	4.45% (4.20, 4.69)	6.29% (6.04, 6.54)	9.1% (8.73, 9.47)	14.12% (13.77, 14.46)	4.15% (4.08, 4.22)

Legend: Observed 5-year risk (in %) for CVD and ASCVD

<1	1-2	2-3	3-4	4-5	5-6	6-7	7-8	8-9	9-10	10-11	11-12	12-13	13-14	14-15	15-16	16-17	17-18

Framingham 2008 general CVD – individuals with diabetes

Reclassification table

Original equation	Re-estimated equation: <1.25%	1.25 – 2.49%	2.50 – 3.74%	3.75 - 4.99%	5.00 – 7.49%	7.50 - 9.99%	≥ 10.00%	Total
**<1.25%**	289 (1.1%)	0 (0.0%)	0 (0.0%)	0 (0.0%)	0 (0.0%)	0 (0.0%)	0 (0.0%)	289 (1.1%)
**1.25 – 2.49%**	583 (2.3%)	131 (0.5%)	11 (0.0%)	3 (0.0%)	1 (0.0%)	0 (0.0%)	0 (0.0%)	729 (2.8%)
**2.50 – 3.74%**	243 (0.9%)	419 (1.6%)	112 (0.4%)	35 (0.1%)	21 (0.1%)	4 (0.0%)	1 (0.0%)	835 (3.3%)
**3.75 - 4.99%**	52 (0.2%)	381 (1.5%)	293 (1.1%)	149 (0.6%)	106 (0.4%)	24 (0.1%)	9 (0.0%)	1,014 (4.0%)
**5.00 – 7.49%**	13 (0.1%)	267 (1.0%)	584 (2.3%)	511 (2.0%)	634 (2.5%)	249 (1.0%)	135 (0.5%)	2,393 (9.3%)
**7.50 - 9.99%**	2 (0.0%)	44 (0.2%)	201 (0.8%)	372 (1.5%)	847 (3.3%)	584 (2.3%)	531 (2.1%)	2,581 (10.1%)
**≥ 10.00%**	0 (0.0%)	13 (0.1%)	74 (0.3%)	254 (1.0%)	1,228 (4.8%)	1,946 (7.6%)	14,270 (55.7%)	17,785 (69.4%)
**Total**	1,182 (4.6%)	1,255 (4.9%)	1,275 (5.0%)	1,324 (5.2%)	2,837 (11.1%)	2,807 (11.0%)	14,946 (58.3%)	25,626 (100%)

Reclassification **NO: 63.1%** **UP: 4.4%** **DOWN: 32.5%**

5-year Kaplan Meier estimate of general CVD with 95% confidence intervals

Original equation	<1.25%	1.25 – 2.49%	2.50 – 3.74%	3.75 - 4.99%	5.00 – 7.49%	7.50 - 9.99%	≥ 10.00%	Total
**<1.25%**	0.44% (0.00, 1.29)							0.44% (0.00, 1.29)
**1.25 – 2.49%**	0.71% (0.01, 1.40)	0.76% (0.00, 2.24)						1.02% (0.26, 1.77)
**2.50 – 3.74%**	2.00% (0.00, 4.88)	2.89% (0.98, 4.77)	3.51% (0.00, 7.55)					3.39% (1.84, 4.92)
**3.75 - 4.99%**		2.18% (0.30, 4.03)	9.1% (4.46, 13.51)	6.38% (1.82, 10.73)	11.33% (3.71, 18.34)			6.30% (4.37, 8.20)
**5.00 – 7.49%**		3.46% (0.33, 6.50)	2.82% (1.04, 4.58)	3.79% (1.83, 5.70)	7.29% (4.76, 9.76)	9.96% (5.35, 14.34)	21.38% (11.56, 30.12)	6.08% (4.87, 7.28)
**7.50 - 9.99%**			3.8% (0.26, 7.21)	5.91% (2.91, 8.81)	6.29% (4.22, 8.31)	11.03% (7.89, 14.05)	13.05% (9.61, 16.35)	8.45% (7.13, 9.75)
**≥ 10.00%**				8.2% (3.31, 12.84)	8.86% (6.89, 10.80)	9.07% (7.55, 10.56)	18.65% (17.89, 19.41)	16.73% (16.07, 17.38)
**Total**	0.89% (0.13, 1.64)	2.45% (1.37, 3.53)	4.48% (2.96, 5.99)	5.73% (4.18, 7.26)	7.86% (6.63, 9.07)	9.76% (8.44, 11.06)	18.47% (17.73, 19.21)	13.52% (13.01, 14.02)

Legend: Observed 5-year risk (in %) for CVD and ASCVD

<1	1-2	2-3	3-4	4-5	5-6	6-7	7-8	8-9	9-10	10-11	11-12	12-13	13-14	14-15	15-16	16-17	17-18

Framingham 2008 general CVD – individuals without hypertension

Reclassification table

Original equation \ Re-estimated equation	<1.25%	1.25 – 2.49%	2.50 – 3.74%	3.75 - 4.99%	5.00 – 7.49%	7.50 - 9.99%	≥ 10.00%	Total
**<1.25%**	99,009 (28.4%)	1,910 (0.5%)	34 (0.0%)	1 (0.0%)	1 (0.0%)	0 (0.0%)	0 (0.0%)	100,955 (29.0%)
**1.25 – 2.49%**	46,259 (13.3%)	32,766 (9.4%)	3,938 (1.1%)	725 (0.2%)	215 (0.1%)	19 (0.0%)	0 (0.0%)	83,922 (24.1%)
**2.50 – 3.74%**	3,494 (1.0%)	25,546 (7.3%)	14,394 (4.1%)	4,623 (1.3%)	2,445 (0.7%)	266 (0.1%)	23 (0.0%)	50,791 (14.6%)
**3.75 - 4.99%**	163 (0.0%)	6,161 (1.8%)	11,858 (3.4%)	7,280 (2.1%)	5,836 (1.7%)	1,246 (0.4%)	161 (0.0%)	32,705 (9.4%)
**5.00 – 7.49%**	10 (0.0%)	1,117 (0.3%)	6,513 (1.9%)	9,004 (2.6%)	12,604 (3.6%)	5,475 (1.6%)	1,715 (0.5%)	36,438 (10.5%)
**7.50 - 9.99%**	0 (0.0%)	13 (0.0%)	540 (0.2%)	1,907 (0.5%)	6,052 (1.7%)	5,347 (1.5%)	4,204 (1.2%)	18,063 (5.2%)
**≥ 10.00%**	0 (0.0%)	0 (0.0%)	33 (0.0%)	263 (0.1%)	2,280 (0.7%)	4,150 (1.2%)	18,619 (5.3%)	25,345 (7.3%)
**Total**	148,935 (42.8%)	67,513 (19.4%)	37,310 (10.7%)	23,803 (6.8%)	29,433 (8.5%)	16,503 (4.7%)	24,722 (7.1%)	348,219 (100%)

**Reclassification**	**NO: 54.6%**	**UP: 9.4%**	**DOWN: 36.0%**

5-year Kaplan Meier estimate of general CVD with 95% confidence intervals

Original equation	<1.25%	1.25 – 2.49%	2.50 – 3.74%	3.75 - 4.99%	5.00 – 7.49%	7.50 - 9.99%	≥ 10.00%	Total
**<1.25%**	0.40% (0.34, 0.45)	1.30% (0.64, 1.95)						0.42% (0.37, 0.47)
**1.25 – 2.49%**	0.75% (0.65, 0.86)	1.42% (1.26, 1.59)	2.89% (2.25, 3.54)	5.02% (3.23, 6.78)	12.18% (6.97, 17.10)			1.20% (1.11, 1.30)
**2.50 – 3.74%**	1.11% (0.64, 1.59)	1.66% (1.46, 1.86)	2.59% (2.26, 2.91)	4.82% (4.08, 5.56)	6.91% (5.70, 8.10)	14.65% (9.08, 19.88)		2.54% (2.36, 2.71)
**3.75 - 4.99%**	3.74% (0.02, 7.33)	1.73% (1.30, 2.15)	2.83% (2.46, 3.21)	3.93% (3.39, 4.46)	5.78% (5.06, 6.49)	10.27% (8.22, 12.27)	16.21% (8.93, 22.90)	3.79% (3.53, 4.04)
**5.00 – 7.49%**		2.67% (1.35, 3.97)	2.85% (2.34, 3.35)	4.24% (3.71, 4.76)	6.18% (5.67, 6.69)	9.67% (8.72, 10.60)	11.85% (10.05, 13.61)	5.86% (5.56, 6.15)
**7.50 - 9.99%**			3.06% (1.32, 4.77)	5.02% (3.78, 6.25)	6.6% (5.83, 7.37)	9.43% (8.49, 10.36)	12.86% (11.66, 14.04)	8.71% (8.21, 9.21)
**≥ 10.00%**				9.41% (5.27, 13.38)	7.84% (6.37, 9.29)	9.06% (8.02, 10.08)	15.87% (15.24, 16.49)	14.03% (13.51, 14.54)
**Total**	0.53% (0.48, 0.58)	1.56% (1.44, 1.68)	2.76% (2.55, 2.96)	4.40% (4.08, 4.72)	6.41% (6.06, 6.75)	9.60% (9.06, 10.13)	15.08% (14.55, 15.61)	3.35% (3.27, 3.42)

Legend: Observed 5-year risk (in %) for CVD and ASCVD

<1	1-2	2-3	3-4	4-5	5-6	6-7	7-8	8-9	9-10	10-11	11-12	12-13	13-14	14-15	15-16	16-17	17-18

Framingham 2008 general CVD – individuals without hypertension

Reclassification table

Original equation	Re-estimated equation: <1.25%	1.25 – 2.49%	2.50 – 3.74%	3.75 - 4.99%	5.00 – 7.49%	7.50 - 9.99%	≥ 10.00%	Total
**<1.25%**	5,853 (3.2%)	11 (0.0%)	0 (0.0%)	0 (0.0%)	0 (0.0%)	0 (0.0%)	0 (0.0%)	5,864 (3.2%)
**1.25 – 2.49%**	14,067 (7.6%)	3,308 (1.8%)	121 (0.1%)	18 (0.0%)	6 (0.0%)	0 (0.0%)	0 (0.0%)	17,520 (9.5%)
**2.50 – 3.74%**	4,369 (2.4%)	11,503 (6.2%)	2,506 (1.4%)	403 (0.2%)	140 (0.1%)	8 (0.0%)	1 (0.0%)	18,930 (10.3%)
**3.75 - 4.99%**	649 (0.4%)	7,723 (4.2%)	6,780 (3.7%)	2,454 (1.3%)	1,162 (0.6%)	169 (0.1%)	13 (0.0%)	18,950 (10.3%)
**5.00 – 7.49%**	89 (0.0%)	3,515 (1.9%)	9,506 (5.2%)	8,745 (4.7%)	8,554 (4.6%)	2,611 (1.4%)	521 (0.3%)	33,541 (18.2%)
**7.50 - 9.99%**	4 (0.0%)	250 (0.1%)	2,147 (1.2%)	4,657 (2.5%)	9,685 (5.3%)	5,742 (3.1%)	2,683 (1.5%)	25,168 (13.6%)
**≥ 10.00%**	0 (0.0%)	33 (0.0%)	357 (0.2%)	1,564 (0.8%)	8,147 (4.4%)	12,066 (6.5%)	42,309 (22.9%)	64,476 (35.0%)
**Total**	25,031 (13.6%)	26,343 (14.3%)	21,417 (11.6%)	17,841 (9.7%)	27,694 (15.0%)	20,596 (11.2%)	45,527 (24.7%)	184,449 (100%)

**Reclassification**	**NO: 38.3%**	**UP: 4.3%**	**DOWN: 57.4%**

5-year Kaplan Meier estimate of general CVD with 95% confidence intervals

Original equation	<1.25%	1.25 – 2.49%	2.50 – 3.74%	3.75 - 4.99%	5.00 – 7.49%	7.50 - 9.99%	≥ 10.00%	Total
**<1.25%**	0.67% (0.41, 0.94)							0.67% (0.41, 0.94)
**1.25 – 2.49%**	0.82% (0.63, 1.02)	1.67% (1.14, 2.21)	5.51% (0.47, 10.30)					1.04% (0.85, 1.23)
**2.50 – 3.74%**	0.98% (0.63, 1.33)	1.67% (1.35, 1.99)	2.51% (1.80, 3.23)	4.74% (2.12, 7.29)	8.19% (2.31, 13.72)			1.76% (1.52, 2.01)
**3.75 - 4.99%**	3.41% (1.23, 5.55)	2.12% (1.73, 2.51)	2.67% (2.18, 3.16)	4.79% (3.75, 5.81)	6.39% (4.79, 7.96)	8.85% (3.55, 13.86)		3.07% (2.77, 3.38)
**5.00 – 7.49%**		2.71% (2.02, 3.41)	2.82% (2.39, 3.24)	4.19% (3.66, 4.72)	5.40% (4.82, 5.98)	10.49% (9.05, 11.91)	12.99% (9.53, 16.31)	4.64% (4.36, 4.92)
**7.50 - 9.99%**		4.09% (1.39, 6.71)	3.10% (2.19, 4.01)	5.07% (4.27, 5.87)	6.20% (5.59, 6.81)	8.80% (7.88, 9.70)	12.55% (11.04, 14.04)	7.06% (6.66, 7.46)
**≥ 10.00%**			6.97% (3.47, 10.34)	5.25% (3.81, 6.68)	7.50% (6.78, 8.22)	8.36% (7.76, 8.96)	15.21% (14.80, 15.62)	12.74% (12.43, 13.06)
**Total**	0.89% (0.74, 1.04)	1.97% (1.75, 2.18)	2.84% (2.56, 3.12)	4.62% (4.23, 5.00)	6.33% (5.98, 6.69)	8.78% (8.30, 9.25)	15.03% (14.63, 15.43)	7.01% (6.86, 7.15)

Legend: Observed 5-year risk (in %) for CVD and ASCVD

<1	1-2	2-3	3-4	4-5	5-6	6-7	7-8	8-9	9-10	10-11	11-12	12-13	13-14	14-15	15-16	16-17	17-18

Pooled Cohort Equations for ASCVD - MEN

Reclassification table

Original equation	Re-estimated equation: **<1.25%**	**1.25 – 2.49%**	**2.50 – 3.74%**	**3.75 - 4.99%**	**5.00 – 7.49%**	**7.50 - 9.99%**	**≥ 10.00%**	**Total**
**<1.25%**	10,403 (5.0%)	28,031 (13.5%)	2,144 (1.0%)	9 (0.0%)	0 (0.0%)	0 (0.0%)	0 (0.0%)	40,587 (19.6%)
**1.25 – 2.49%**	0 (0.0%)	5,718 (2.8%)	24,111 (11.6%)	7,731 (3.7%)	705 (0.3%)	6 (0.0%)	0 (0.0%)	38,271 (18.5%)
**2.50 – 3.74%**	0 (0.0%)	0 (0.0%)	2,840 (1.4%)	12,600 (6.1%)	10,824 (5.2%)	482 (0.2%)	12 (0.0%)	26,758 (12.9%)
**3.75 - 4.99%**	0 (0.0%)	0 (0.0%)	31 (0.0%)	2,312 (1.1%)	13,512 (6.5%)	4,085 (2.0%)	317 (0.2%)	20,257 (9.8%)
**5.00 – 7.49%**	0 (0.0%)	0 (0.0%)	0 (0.0%)	234 (0.1%)	7,219 (3.5%)	13,922 (6.7%)	7,686 (3.7%)	29,061 (14.0%)
**7.50 - 9.99%**	0 (0.0%)	0 (0.0%)	0 (0.0%)	0 (0.0%)	458 (0.2%)	3,600 (1.7%)	14,894 (7.2%)	18,952 (9.1%)
**≥ 10.00%**	0 (0.0%)	0 (0.0%)	0 (0.0%)	0 (0.0%)	21 (0.0%)	548 (0.3%)	32,858 (15.8%)	33,427 (16.1%)
**Total**	10,403 (5.0%)	33,749 (16.3%)	29,126 (14.0%)	22,886 (11.0%)	32,739 (15.8%)	22,643 (10.9%)	55,767 (26.9%)	207,313 (100%)

**Reclassification**	**NO: 31.3%**	**UP: 68.1%**	**DOWN: 0.6%**

5-year Kaplan Meier estimate of general CVD with 95% confidence intervals

Original equation	<1.25%	1.25 – 2.49%	2.50 – 3.74%	3.75 - 4.99%	5.00 – 7.49%	7.50 - 9.99%	≥ 10.00%	Total
**<1.25%**	1.00% (0.76, 1.25)	1.64% (1.45, 1.84)	3.86% (2.82, 4.90)					1.60% (1.44, 1.76)
**1.25 – 2.49%**		1.85% (1.41, 2.29)	2.56% (2.31, 2.81)	4.69% (4.11, 5.27)	6.78% (4.56, 8.94)			2.97% (2.76, 3.18)
**2.50 – 3.74%**			2.97% (2.22, 3.70)	4.20% (3.76, 4.63)	5.21% (4.71, 5.72)	10.33% (7.12, 13.43)		4.64% (4.33, 4.94)
**3.75 - 4.99%**				3.51% (2.54, 4.47)	6.13% (5.62, 6.63)	9.04% (7.98, 10.09)	19.3% (14.39, 23.93)	6.65% (6.23, 7.08)
**5.00 – 7.49%**				4.35% (1.47, 7.15)	6.62% (5.91, 7.32)	8.11% (7.56, 8.65)	11.74% (10.89, 12.58)	8.75% (8.36, 9.14)
**7.50 - 9.99%**					8.21% (5.29, 11.04)	9.54% (8.35, 10.72)	12.70% (12.06, 13.33)	12.04% (11.48, 12.60)
**≥ 10.00%**						12.68% (9.37, 15.87)	17.16% (16.67, 17.64)	17.08% (16.60, 17.56)
**Total**	1.00% (0.76, 1.25)	1.68% (1.50, 1.86)	2.70% (2.47, 2.93)	4.30% (3.97, 4.63)	5.97% (5.65, 6.28)	8.65% (8.21, 9.10)	15.22% (14.87, 15.57)	7.33% (7.19, 7.47)

Legend: Observed 5-year risk (in %) for CVD and ASCVD

<1	1-2	2-3	3-4	4-5	5-6	6-7	7-8	8-9	9-10	10-11	11-12	12-13	13-14	14-15	15-16	16-17	17-18

Pooled Cohort Equations for ASCVD – WOMEN

Reclassification table

Re-estimated equation

Original equation	<1.25%	1.25 – 2.49%	2.50 – 3.74%	3.75 - 4.99%	5.00 – 7.49%	7.50 - 9.99%	≥ 10.00%	Total
**<1.25%**	74,164 (31.2%)	46,836 (19.7%)	9,384 (3.9%)	496 (0.2%)	34 (0.0%)	0 (0.0%)	0 (0.0%)	130,914 (55.0%)
**1.25 – 2.49%**	13 (0.0%)	5,797 (2.4%)	16,796 (7.1%)	12,445 (5.2%)	4,909 (2.1%)	179 (0.1%)	14 (0.0%)	40,153 (16.9%)
**2.50 – 3.74%**	0 (0.0%)	168 (0.1%)	1,418 (0.6%)	4,134 (1.7%)	12,127 (5.1%)	2,871 (1.2%)	293 (0.1%)	21,011 (8.8%)
**3.75 - 4.99%**	0 (0.0%)	14 (0.0%)	145 (0.1%)	442 (0.2%)	4,890 (2.1%)	5,840 (2.5%)	1,849 (0.8%)	13,180 (5.5%)
**5.00 – 7.49%**	0 (0.0%)	3 (0.0%)	18 (0.0%)	95 (0.0%)	1,189 (0.5%)	5,239 (2.2%)	8,448 (3.6%)	14,992 (6.3%)
**7.50 - 9.99%**	0 (0.0%)	0 (0.0%)	0 (0.0%)	7 (0.0%)	55 (0.0%)	527 (0.2%)	7,454 (3.1%)	8,043 (3.4%)
**≥ 10.00%**	0 (0.0%)	0 (0.0%)	0 (0.0%)	6 (0.0%)	5 (0.0%)	44 (0.0%)	9,524 (4.0%)	9,579 (4.0%)
**Total**	74,177 (31.2%)	52,818 (22.2%)	27,761 (11.7%)	17,625 (7.4%)	23,209 (9.8%)	14,700 (6.2%)	27,582 (11.6%)	237,872 (100%)

Reclassification **NO: 39.1%** **UP: 60.7%** **DOWN: 0.2%**

5-year Kaplan Meier estimate of general CVD with 95% confidence intervals

Original equation	<1.25%	1.25 – 2.49%	2.50 – 3.74%	3.75 - 4.99%	5.00 – 7.49%	7.50 - 9.99%	≥ 10.00%	Total
**<1.25%**	0.75% (0.67, 0.83)	1.52% (1.38, 1.65)	2.53% (2.14, 2.92)	3.88% (1.85, 5.87)				1.18% (1.11, 1.25)
**1.25 – 2.49%**		2.66% (2.15, 3.17)	2.93% (2.62, 3.24)	4.38% (3.95, 4.81)	5.56% (4.82, 6.29)	7.43% (3.01, 11.64)		3.70% (3.48, 3.93)
**2.50 – 3.74%**			5.30% (3.87, 6.72)	4.36% (3.62, 5.09)	5.73% (5.24, 6.22)	9.64% (8.36, 10.91)	12.19% (7.92, 16.26)	6.05% (5.67, 6.43)
**3.75 - 4.99%**			6.36% (0.52, 11.86)	10.66% (7.07, 14.11)	6.24% (5.42, 7.06)	7.38% (6.57, 8.18)	12.33% (10.54, 14.07)	7.77% (7.22, 8.31)
**5.00 – 7.49%**					9.2% (7.22, 11.13)	8.72% (7.81, 9.63)	11.97% (11.11, 12.82)	10.61% (10.01, 11.21)
**7.50 - 9.99%**						12.57% (8.65, 16.32)	15.55% (14.56, 16.53)	15.33% (14.37, 16.28)
**≥ 10.00%**							18.68% (17.73, 19.61)	18.74% (17.80, 19.67)
**Total**	0.75% (0.67, 0.83)	1.64% (1.51, 1.77)	2.94% (2.70, 3.18)	4.51% (4.14, 4.87)	6.03% (5.67, 6.39)	8.57% (8.02, 9.11)	15.28% (14.77, 15.79)	4.28% (4.18, 4.38)

Legend: Observed 5-year risk (in %) for CVD and ASCVD

<1	1-2	2-3	3-4	4-5	5-6	6-7	7-8	8-9	9-10	10-11	11-12	12-13	13-14	14-15	15-16	16-17	17-18

Pooled Cohort Equations for ASCVD – AGE 40 to 49

Reclassification table

Original equation	Re-estimated equation: <1.25%	1.25 – 2.49%	2.50 – 3.74%	3.75 - 4.99%	5.00 – 7.49%	7.50 - 9.99%	≥ 10.00%	Total
**<1.25%**	72,272 (48.5%)	37,377 (25.1%)	664 (0.4%)	0 (0.0%)	0 (0.0%)	0 (0.0%)	0 (0.0%)	110,313 (74.0%)
**1.25 – 2.49%**	13 (0.0%)	8,823 (5.9%)	13,800 (9.3%)	920 (0.6%)	8 (0.0%)	0 (0.0%)	0 (0.0%)	23,564 (15.8%)
**2.50 – 3.74%**	0 (0.0%)	161 (0.1%)	2,926 (2.0%)	4,429 (3.0%)	387 (0.3%)	0 (0.0%)	0 (0.0%)	7,903 (5.3%)
**3.75 - 4.99%**	0 (0.0%)	14 (0.0%)	149 (0.1%)	1,794 (1.2%)	1,678 (1.1%)	5 (0.0%)	0 (0.0%)	3,640 (2.4%)
**5.00 – 7.49%**	0 (0.0%)	3 (0.0%)	18 (0.0%)	273 (0.2%)	2,161 (1.4%)	160 (0.1%)	0 (0.0%)	2,615 (1.8%)
**7.50 - 9.99%**	0 (0.0%)	0 (0.0%)	0 (0.0%)	7 (0.0%)	351 (0.2%)	289 (0.2%)	11 (0.0%)	658 (0.4%)
**≥ 10.00%**	0 (0.0%)	0 (0.0%)	0 (0.0%)	6 (0.0%)	23 (0.0%)	205 (0.1%)	143 (0.1%)	377 (0.3%)
**Total**	72,285 (48.5%)	46,378 (31.1%)	17,557 (11.8%)	7,429 (5.0%)	4,608 (3.1%)	659 (0.4%)	154 (0.1%)	149,070 (100%)

**Reclassification**	**NO: 59.3%**	**UP: 39.9%**	**DOWN: 0.8%**

5-year Kaplan Meier estimate of general CVD with 95% confidence intervals

Original equation	<1.25%	1.25 – 2.49%	2.50 – 3.74%	3.75 - 4.99%	5.00 – 7.49%	7.50 - 9.99%	≥ 10.00%	Total
**<1.25%**	0.74% (0.66, 0.81)	1.43% (1.28, 1.59)	3.31% (1.64, 4.96)					0.99% (0.91, 1.06)
**1.25 – 2.49%**		2.14% (1.76, 2.51)	2.22% (1.91, 2.52)	3.4% (2.14, 4.65)				2.24% (2.01, 2.47)
**2.50 – 3.74%**			3.21% (2.45, 3.97)	4.29% (3.59, 5.00)	1.14% (0.02, 2.26)			3.66% (3.17, 4.15)
**3.75 - 4.99%**			6.58% (0.48, 12.30)	3.2% (2.17, 4.22)	6.74% (5.10, 8.36)			5.09% (4.13, 6.04)
**5.00 – 7.49%**				4.42% (1.78, 6.98)	6.29% (4.94, 7.62)	19.03% (11.91, 25.57)		6.97% (5.73, 8.20)
**7.50 - 9.99%**					8.53% (5.25, 11.69)	7.75% (4.07, 11.29)		8.02% (5.62, 10.35)
**≥ 10.00%**						10.67% (5.24, 15.79)	9.17% (3.57, 14.45)	9.2% (5.63, 12.64)
**Total**	0.74% (0.66, 0.81)	1.57% (1.42, 1.71)	2.48% (2.19, 2.76)	3.92% (3.39, 4.43)	6.21% (5.29, 7.12)	11.59% (8.67, 14.41)	8.57% (3.31, 13.55)	1.58% (1.50, 1.66)

Legend: Observed 5-year risk (in %) for CVD and ASCVD

<1	1-2	2-3	3-4	4-5	5-6	6-7	7-8	8-9	9-10	10-11	11-12	12-13	13-14	14-15	15-16	16-17	17-18

Pooled Cohort Equations for ASCVD – AGE50 to 59

Reclassification table

Re-estimated equation (columns); Original equation (rows)

Original equation	<1.25%	1.25 – 2.49%	2.50 – 3.74%	3.75 - 4.99%	5.00 – 7.49%	7.50 - 9.99%	≥ 10.00%	Total
**<1.25%**	12,295 (9.3%)	35,379 (26.7%)	6,642 (5.0%)	146 (0.1%)	3 (0.0%)	0 (0.0%)	0 (0.0%)	54,465 (41.2%)
**1.25 – 2.49%**	0 (0.0%)	2,567 (1.9%)	20,191 (15.3%)	9,011 (6.8%)	787 (0.6%)	2 (0.0%)	0 (0.0%)	32,558 (24.6%)
**2.50 – 3.74%**	0 (0.0%)	7 (0.0%)	1,234 (0.9%)	9,684 (7.3%)	8,258 (6.2%)	124 (0.1%)	1 (0.0%)	19,308 (14.6%)
**3.75 - 4.99%**	0 (0.0%)	0 (0.0%)	26 (0.0%)	872 (0.7%)	8,792 (6.6%)	1,019 (0.8%)	10 (0.0%)	10,719 (8.1%)
**5.00 – 7.49%**	0 (0.0%)	0 (0.0%)	0 (0.0%)	53 (0.0%)	4,345 (3.3%)	4,701 (3.6%)	505 (0.4%)	9,604 (7.3%)
**7.50 - 9.99%**	0 (0.0%)	0 (0.0%)	0 (0.0%)	0 (0.0%)	152 (0.1%)	1,924 (1.5%)	1,262 (1.0%)	3,338 (2.5%)
**≥ 10.00%**	0 (0.0%)	0 (0.0%)	0 (0.0%)	0 (0.0%)	3 (0.0%)	308 (0.2%)	1,977 (1.5%)	2,288 (1.7%)
**Total**	12,295 (9.3%)	37,953 (28.7%)	28,093 (21.2%)	19,766 (14.9%)	22,340 (16.9%)	8,078 (6.1%)	3,755 (2.8%)	132,280 (100%)

Reclassification — **NO: 19.1%** **UP: 80.5%** **DOWN: 0.4%**

5-year Kaplan Meier estimate of general CVD with 95% confidence intervals

Original equation	<1.25%	1.25 – 2.49%	2.50 – 3.74%	3.75 - 4.99%	5.00 – 7.49%	7.50 - 9.99%	≥ 10.00%	Total
**<1.25%**	1.06% (0.81, 1.31)	1.66% (1.49, 1.82)	2.97% (2.48, 3.45)	1.64% (0.00, 3.89)				1.69% (1.56, 1.83)
**1.25 – 2.49%**		2.65% (1.89, 3.41)	2.93% (2.64, 3.21)	4.54% (4.01, 5.06)	5.82% (3.93, 7.68)			3.43% (3.18, 3.67)
**2.50 – 3.74%**			4.95% (3.44, 6.44)	4.34% (3.83, 4.86)	5.69% (5.07, 6.30)	15.69% (8.49, 22.33)		5.04% (4.66, 5.42)
**3.75 - 4.99%**				6.05% (3.94, 8.11)	6.12% (5.50, 6.75)	8.10% (6.01, 10.15)		6.30% (5.72, 6.87)
**5.00 – 7.49%**					7.60% (6.65, 8.54)	9.18% (8.19, 10.16)	9.15% (6.26, 11.94)	8.45% (7.79, 9.12)
**7.50 - 9.99%**					9.47% (3.72, 14.87)	9.67% (7.99, 11.32)	11.79% (9.71, 13.83)	10.48% (9.21, 11.73)
**≥ 10.00%**						16.63% (11.50, 21.46)	10.98% (9.26, 12.67)	11.74% (10.10, 13.34)
**Total**	1.06% (0.81, 1.31)	1.72% (1.56, 1.88)	3.02% (2.78, 3.27)	4.48% (4.12, 4.84)	6.27% (5.88, 6.66)	9.56% (8.78, 10.33)	10.96% (9.76, 12.14)	3.86% (3.74, 3.99)

Legend: Observed 5-year risk (in %) for CVD and ASCVD

<1	1-2	2-3	3-4	4-5	5-6	6-7	7-8	8-9	9-10	10-11	11-12	12-13	13-14	14-15	15-16	16-17	17-18

Pooled Cohort Equations for ASCVD – AGE 60 to 69

Reclassification table

Original equation		Re-estimated equation: <1.25%	1.25 – 2.49%	2.50 – 3.74%	3.75 - 4.99%	5.00 – 7.49%	7.50 - 9.99%	≥ 10.00%	Total
	**<1.25%**	0 (0.0%)	2,111 (2.1%)	4,222 (4.2%)	359 (0.4%)	31 (0.0%)	0 (0.0%)	0 (0.0%)	6,723 (6.6%)
	**1.25 – 2.49%**	0 (0.0%)	125 (0.1%)	6,916 (6.8%)	10,203 (10.1%)	4,616 (4.5%)	155 (0.2%)	7 (0.0%)	22,022 (21.7%)
	**2.50 – 3.74%**	0 (0.0%)	0 (0.0%)	98 (0.1%)	2,573 (2.5%)	12,330 (12.1%)	2,056 (2.0%)	134 (0.1%)	17,191 (16.9%)
	**3.75 - 4.99%**	0 (0.0%)	0 (0.0%)	1 (0.0%)	87 (0.1%)	6,734 (6.6%)	5,805 (5.7%)	762 (0.8%)	13,389 (13.2%)
	**5.00 – 7.49%**	0 (0.0%)	0 (0.0%)	0 (0.0%)	3 (0.0%)	1,658 (1.6%)	11,148 (11.0%)	7,300 (7.2%)	20,109 (19.8%)
	**7.50 - 9.99%**	0 (0.0%)	0 (0.0%)	0 (0.0%)	0 (0.0%)	8 (0.0%)	1,637 (1.6%)	9,543 (9.4%)	11,188 (11.0%)
	**≥ 10.00%**	0 (0.0%)	0 (0.0%)	0 (0.0%)	0 (0.0%)	0 (0.0%)	64 (0.1%)	10,831 (10.7%)	10,895 (10.7%)
	**Total**	0 (0.0%)	2,236 (2.2%)	11,237 (11.1%)	13,225 (13.0%)	25377 (25.0%)	20,865 (20.6%)	28,577 (28.1%)	101,517 (100%)

**Reclassification**	**NO: 14.2%**	**UP: 85.7%**	**DOWN: 0.1%**

5-year Kaplan Meier estimate of general CVD with 95% confidence intervals

Original equation		<1.25%	1.25 – 2.49%	2.50 – 3.74%	3.75 - 4.99%	5.00 – 7.49%	7.50 - 9.99%	≥ 10.00%	Total
	**<1.25%**		2.26% (1.56, 2.97)	2.36% (1.77, 2.94)	5.03% (2.32, 7.68)				2.59% (2.12, 3.06)
	**1.25 – 2.49%**		2.89% (0.00, 6.17)	3.06% (2.58, 3.54)	4.54% (4.05, 5.03)	5.36% (4.61, 6.10)	4.37% (0.87, 7.74)		4.25% (3.93, 4.57)
	**2.50 – 3.74%**				3.84% (2.96, 4.70)	5.16% (4.71, 5.62)	9.56% (8.06, 11.03)	16.01% (8.83, 22.62)	5.60% (5.20, 6.00)
	**3.75 - 4.99%**					6.18% (5.48, 6.87)	8.15% (7.30, 8.99)	11.97% (9.41, 14.46)	7.46% (6.93, 7.99)
	**5.00 – 7.49%**					5.85% (4.53, 7.14)	7.68% (7.07, 8.27)	10.93% (10.07, 11.77)	8.74% (8.28, 9.21)
	**7.50 - 9.99%**						9.94% (8.11, 11.73)	11.84% (11.07, 12.61)	11.57% (10.86, 12.27)
	**≥ 10.00%**							13.62% (12.86, 14.38)	13.64% (12.88, 14.39)
	**Total**		2.3% (1.60, 2.99)	2.81% (2.44, 3.18)	4.53% (4.10, 4.95)	5.54% (5.21, 5.87)	8.17% (7.72, 8.62)	12.3% (11.85, 12.75)	7.52% (7.32, 7.71)

Legend: Observed 5-year risk (in %) for CVD and ASCVD

<1	1-2	2-3	3-4	4-5	5-6	6-7	7-8	8-9	9-10	10-11	11-12	12-13	13-14	14-15	15-16	16-17	17-18

Pooled Cohort Equations for ASCVD – AGE 70 to 79

Reclassification table

Original equation	Re-estimated equation: <1.25%	1.25 – 2.49%	2.50 – 3.74%	3.75 - 4.99%	5.00 – 7.49%	7.50 - 9.99%	≥ 10.00%	Total
**<1.25%**	0 (0.0%)	0 (0.0%)	0 (0.0%)	0 (0.0%)	0 (0.0%)	0 (0.0%)	0 (0.0%)	0 (0.0%)
**1.25 – 2.49%**	0 (0.0%)	0 (0.0%)	0 (0.0%)	42 (0.1%)	203 (0.3%)	28 (0.0%)	7 (0.0%)	280 (0.4%)
**2.50 – 3.74%**	0 (0.0%)	0 (0.0%)	0 (0.0%)	48 (0.1%)	1,976 (3.2%)	1,173 (1.9%)	170 (0.3%)	3,367 (5.4%)
**3.75 - 4.99%**	0 (0.0%)	0 (0.0%)	0 (0.0%)	1 (0.0%)	1,198 (1.9%)	3,096 (5.0%)	1,394 (2.2%)	5,689 (9.1%)
**5.00 – 7.49%**	0 (0.0%)	0 (0.0%)	0 (0.0%)	0 (0.0%)	244 (0.4%)	3,152 (5.1%)	8,329 (13.4%)	11,725 (18.8%)
**7.50 - 9.99%**	0 (0.0%)	0 (0.0%)	0 (0.0%)	0 (0.0%)	2 (0.0%)	277 (0.4%)	11,532 (18.5%)	11,811 (19.0%)
**≥ 10.00%**	0 (0.0%)	0 (0.0%)	0 (0.0%)	0 (0.0%)	0 (0.0%)	15 (0.0%)	29,431 (47.2%)	29,446 (47.3%)
**Total**	0 (0.0%)	0 (0.0%)	0 (0.0%)	91 (0.1%)	3,623 (5.8%)	7,741 (12.4%)	50,863 (81.6%)	62,318 (100%)

Reclassification **NO: 48.1%** **UP: 51.9%** **DOWN: <0.1%**

5-year Kaplan Meier estimate of general CVD with 95% confidence intervals

Original equation	<1.25%	1.25 – 2.49%	2.50 – 3.74%	3.75 - 4.99%	5.00 – 7.49%	7.50 - 9.99%	≥ 10.00%	Total
**<1.25%**								
**1.25 – 2.49%**					12.19% (6.84, 17.23)			11.88% (7.42, 16.12)
**2.50 – 3.74%**					7.62% (6.14, 9.07)	9.41% (7.36, 11.42)	12.73% (6.93, 18.17)	8.41% (7.25, 9.56)
**3.75 - 4.99%**					5.51% (4.01, 7.00)	7.85% (6.72, 8.97)	14.17% (11.95, 16.33)	8.93% (8.04, 9.80)
**5.00 – 7.49%**					10.42% (5.39, 15.19)	8.49% (7.32, 9.64)	12.83% (11.95, 13.70)	11.67% (10.96, 12.38)
**7.50 - 9.99%**						13.03% (8.08, 17.71)	15.36% (14.56, 16.15)	15.31% (14.52, 16.09)
**≥ 10.00%**							19.37% (18.83, 19.91)	19.37% (18.83, 19.91)
**Total**					7.40% (6.35, 8.45)	8.62% (7.87, 9.36)	17.22% (16.83, 17.61)	15.6% (15.25, 15.94)

Legend: Observed 5-year risk (in %) for CVD and ASCVD

<1	1-2	2-3	3-4	4-5	5-6	6-7	7-8	8-9	9-10	10-11	11-12	12-13	13-14	14-15	15-16	16-17	17-18

Pooled Cohort Equations for ASCVD – individuals without diabetes

Reclassification table

Original equation	Re-estimated equation: **<1.25%**	**1.25 – 2.49%**	**2.50 – 3.74%**	**3.75 - 4.99%**	**5.00 – 7.49%**	**7.50 - 9.99%**	**≥ 10.00%**	**Total**
**<1.25%**	84,091 (20.2%)	73,800 (17.8%)	11,319 (2.7%)	493 (0.1%)	33 (0.0%)	0 (0.0%)	0 (0.0%)	169,736 (40.8%)
**1.25 – 2.49%**	13 (0.0%)	11,156 (2.7%)	39,763 (9.6%)	19,519 (4.7%)	5,363 (1.3%)	172 (0.0%)	13 (0.0%)	75,999 (18.3%)
**2.50 – 3.74%**	0 (0.0%)	155 (0.0%)	4,009 (1.0%)	15,989 (3.8%)	21,924 (5.3%)	3,177 (0.8%)	290 (0.1%)	45,544 (11.0%)
**3.75 - 4.99%**	0 (0.0%)	13 (0.0%)	145 (0.0%)	2,505 (0.6%)	17,307 (4.2%)	9,312 (2.2%)	2,027 (0.5%)	31,309 (7.5%)
**5.00 – 7.49%**	0 (0.0%)	3 (0.0%)	11 (0.0%)	248 (0.1%)	7,437 (1.8%)	17,659 (4.2%)	14,874 (3.6%)	40,232 (9.7%)
**7.50 - 9.99%**	0 (0.0%)	0 (0.0%)	0 (0.0%)	2 (0.0%)	358 (0.1%)	3,353 (0.8%)	19,933 (4.8%)	23,646 (5.7%)
**≥ 10.00%**	0 (0.0%)	0 (0.0%)	0 (0.0%)	4 (0.0%)	8 (0.0%)	337 (0.1%)	28,787 (6.9%)	29,136 (7.0%)
**Total**	84,104 (20.2%)	85,127 (20.5%)	55,247 (13.3%)	38,760 (9.3%)	52,430 (12.6%)	34,010 (8.2%)	65,924 (15.9%)	415,602 (100%)

**Reclassification**	**NO: 34.0%**	**UP: 65.7%**	**DOWN: 0.3%**

5-year Kaplan Meier estimate of general CVD with 95% confidence intervals

Original equation	<1.25%	1.25 – 2.49%	2.50 – 3.74%	3.75 - 4.99%	5.00 – 7.49%	7.50 - 9.99%	≥ 10.00%	Total
**<1.25%**	0.78% (0.71, 0.86)	1.55% (1.44, 1.66)	2.70% (2.33, 3.06)	4.18% (2.07, 6.25)				1.27% (1.20, 1.33)
**1.25 – 2.49%**		2.24% (1.90, 2.57)	2.68% (2.49, 2.88)	4.52% (4.17, 4.88)	5.66% (4.94, 6.37)	6.88% (2.59, 10.98)		3.33% (3.18, 3.49)
**2.50 – 3.74%**			3.63% (2.94, 4.31)	4.20% (3.82, 4.59)	5.39% (5.03, 5.75)	9.41% (8.20, 10.60)	14.91% (10.20, 19.37)	5.19% (4.94, 5.43)
**3.75 - 4.99%**			3.85% (0.00, 8.08)	4.30% (3.29, 5.30)	6.14% (5.69, 6.59)	8.02% (7.36, 8.68)	13.81% (12.05, 15.53)	7.08% (6.73, 7.43)
**5.00 – 7.49%**				4.35% (1.61, 7.01)	7.14% (6.42, 7.85)	8.15% (7.66, 8.63)	11.55% (10.95, 12.16)	9.27% (8.93, 9.61)
**7.50 - 9.99%**					8.95% (5.47, 12.30)	9.42% (8.15, 10.67)	13.57% (13, 14.14)	12.96% (12.44, 13.47)
**≥ 10.00%**						10.87% (6.79, 14.76)	16.59% (16.07, 17.10)	16.53% (16.01, 17.04)
**Total**	0.78% (0.71, 0.86)	1.64% (1.53, 1.75)	2.76% (2.60, 2.93)	4.37% (4.12, 4.62)	5.94% (5.69, 6.19)	8.37% (8.02, 8.73)	14.42% (14.10, 14.74)	5.11% (5.03, 5.19)

Legend: Observed 5-year risk (in %) for CVD and ASCVD

<1	1-2	2-3	3-4	4-5	5-6	6-7	7-8	8-9	9-10	10-11	11-12	12-13	13-14	14-15	15-16	16-17	17-18

Pooled Cohort Equations for ASCVD – – individuals with diabetes

Reclassification table

Original equation	Re-estimated equation: **<1.25%**	**1.25 – 2.49%**	**2.50 – 3.74%**	**3.75 - 4.99%**	**5.00 – 7.49%**	**7.50 - 9.99%**	**≥ 10.00%**	**Total**
**<1.25%**	476 (1.6%)	1,067 (3.6%)	209 (0.7%)	12 (0.0%)	1 (0.0%)	0 (0.0%)	0 (0.0%)	1,765 (6.0%)
**1.25 – 2.49%**	0 (0.0%)	359 (1.2%)	1,144 (3.9%)	657 (2.2%)	251 (0.8%)	13 (0.0%)	1 (0.0%)	2,425 (8.2%)
**2.50 – 3.74%**	0 (0.0%)	13 (0.0%)	249 (0.8%)	745 (2.5%)	1,027 (3.5%)	176 (0.6%)	15 (0.1%)	2,225 (7.5%)
**3.75 - 4.99%**	0 (0.0%)	1 (0.0%)	31 (0.1%)	249 (0.8%)	1,095 (3.7%)	613 (2.1%)	139 (0.5%)	2,128 (7.2%)
**5.00 – 7.49%**	0 (0.0%)	0 (0.0%)	7 (0.0%)	81 (0.3%)	971 (3.3%)	1,502 (5.1%)	1,260 (4.3%)	3,821 (12.9%)
**7.50 - 9.99%**	0 (0.0%)	0 (0.0%)	0 (0.0%)	5 (0.0%)	155 (0.5%)	774 (2.6%)	2,415 (8.2%)	3,349 (11.3%)
**≥ 10.00%**	0 (0.0%)	0 (0.0%)	0 (0.0%)	2 (0.0%)	18 (0.1%)	255 (0.9%)	13,595 (46.0%)	13,870 (46.9%)
**Total**	476 (1.6%)	1,440 (4.9%)	1,640 (5.5%)	1,751 (5.9%)	3,518 (11.9%)	3,333 (11.3%)	17,425 (58.9%)	29,583 (100%)

**Reclassification**	**NO: 56.4%**	**UP: 41.7%**	**DOWN: 1.9%**

5-year Kaplan Meier estimate of general CVD with 95% confidence intervals

Original equation	**<1.25%**	**1.25 – 2.49%**	**2.50 – 3.74%**	**3.75 - 4.99%**	**5.00 – 7.49%**	**7.50 - 9.99%**	**≥ 10.00%**	**Total**
**<1.25%**	0.42% (0.00, 1.00)	2.21% (1.07, 3.34)	6.25% (2.30, 10.04)					2.25% (1.37, 3.12)
**1.25 – 2.49%**		2.98% (0.55, 5.35)	3.80% (2.39, 5.20)	3.96% (2.37, 5.53)	6.82% (3.18, 10.32)			4.06% (3.12, 5.00)
**2.50 – 3.74%**			5.31% (1.39, 9.06)	4.82% (3.03, 6.58)	7.65% (5.70, 9.55)	15.70% (9.36, 21.60)		7.03% (5.76, 8.29)
**3.75 - 4.99%**				8.24% (3.84, 12.44)	6.57% (4.96, 8.16)	8.67% (5.98, 11.28)	6.10% (1.54, 10.45)	7.40% (6.10, 8.67)
**5.00 – 7.49%**					5.86% (4.08, 7.61)	9.8% (8.02, 11.54)	15.40% (12.56, 18.14)	10.62% (9.34, 11.88)
**7.50 - 9.99%**					8.63% (3.74, 13.28)	12.00% (9.25, 14.67)	14.22% (12.54, 15.86)	13.46% (12.07, 14.82)
**≥ 10.00%**						19.04% (13.25, 24.45)	19.43% (18.64, 20.21)	19.40% (18.62, 20.17)
**Total**	0.42% (0.00, 1.00)	2.37% (1.34, 3.39)	4.49% (3.20, 5.77)	4.88% (3.72, 6.02)	6.82% (5.85, 7.78)	11.15% (9.89, 12.40)	18.30% (17.61, 18.98)	13.60% (13.14, 14.07)

Legend: Observed 5-year risk (in %) for CVD and ASCVD

<1	1-2	2-3	3-4	4-5	5-6	6-7	7-8	8-9	9-10	10-11	11-12	12-13	13-14	14-15	15-16	16-17	17-18

Pooled Cohort Equations for ASCVD – individuals without hypertension

Reclassification table

Original equation	Re-estimated equation: **<1.25%**	**1.25 – 2.49%**	**2.50 – 3.74%**	**3.75 - 4.99%**	**5.00 – 7.49%**	**7.50 - 9.99%**	**≥ 10.00%**	**Total**
**<1.25%**	70,981 (27.0%)	53,889 (20.5%)	8,806 (3.4%)	431 (0.2%)	33 (0.0%)	0 (0.0%)	0 (0.0%)	134,140 (51.1%)
**1.25 – 2.49%**	8 (0.0%)	6,080 (2.3%)	23,960 (9.1%)	12,898 (4.9%)	4,064 (1.5%)	157 (0.1%)	14 (0.0%)	47,181 (18.0%)
**2.50 – 3.74%**	0 (0.0%)	69 (0.0%)	1,582 (0.6%)	7,924 (3.0%)	13,267 (5.1%)	2,387 (0.9%)	253 (0.1%)	25,482 (9.7%)
**3.75 - 4.99%**	0 (0.0%)	3 (0.0%)	33 (0.0%)	932 (0.4%)	8,084 (3.1%)	5,527 (2.1%)	1,523 (0.6%)	16,102 (6.1%)
**5.00 – 7.49%**	0 (0.0%)	2 (0.0%)	3 (0.0%)	71 (0.0%)	2,426 (0.9%)	7,662 (2.9%)	8,898 (3.4%)	19,062 (7.3%)
**7.50 - 9.99%**	0 (0.0%)	0 (0.0%)	0 (0.0%)	1 (0.0%)	85 (0.0%)	805 (0.3%)	9,342 (3.6%)	10,233 (3.9%)
**≥ 10.00%**	0 (0.0%)	0 (0.0%)	0 (0.0%)	3 (0.0%)	2 (0.0%)	70 (0.0%)	10,255 (3.9%)	10,330 (3.9%)
**Total**	70,989 (27.0%)	60,043 (22.9%)	34,384 (13.1%)	22,260 (8.5%)	27,961 (10.7%)	16,608 (6.3%)	30,285 (11.5%)	262,530 (100%)

**Reclassification**	**NO: 35.5%**	**UP: 64.4%**	**DOWN: 0.1%**

5-year Kaplan Meier estimate of general CVD with 95% confidence intervals

Original equation	<1.25%	1.25 – 2.49%	2.50 – 3.74%	3.75 - 4.99%	5.00 – 7.49%	7.50 - 9.99%	≥ 10.00%	Total
**<1.25%**	0.73% (0.65, 0.81)	1.43% (1.30, 1.56)	2.95% (2.52, 3.38)	4.77% (2.36, 7.13)				1.19% (1.11, 1.26)
**1.25 – 2.49%**		1.84% (1.42, 2.25)	2.56% (2.31, 2.80)	4.26% (3.83, 4.68)	5.75% (4.91, 6.57)	8.32% (3.39, 12.99)		3.25% (3.05, 3.44)
**2.50 – 3.74%**			3.85% (2.69, 5.00)	3.86% (3.34, 4.38)	5.47% (5.00, 5.93)	9.45% (8.07, 10.80)	14.13% (9.22, 18.77)	5.35% (5.02, 5.69)
**3.75 - 4.99%**				3.79% (2.22, 5.33)	6.17% (5.52, 6.82)	8.32% (7.43, 9.20)	13.93% (11.85, 15.97)	7.53% (7.03, 8.02)
**5.00 – 7.49%**					6.2% (5.08, 7.31)	8.30% (7.56, 9.04)	12.59% (11.75, 13.42)	10.11% (9.59, 10.63)
**7.50 - 9.99%**						10.14% (7.74, 12.49)	14.58% (13.72, 15.43)	14.24% (13.43, 15.04)
**≥ 10.00%**							19.20% (18.30, 20.10)	19.10% (18.20, 19.99)
**Total**	0.73% (0.65, 0.81)	1.47% (1.35, 1.59)	2.72% (2.51, 2.93)	4.11% (3.80, 4.43)	5.82% (5.49, 6.15)	8.55% (8.04, 9.06)	15.52% (15.04, 16.01)	4.32% (4.22, 4.41)

Legend: Observed 5-year risk (in %) for CVD and ASCVD

<1	1-2	2-3	3-4	4-5	5-6	6-7	7-8	8-9	9-10	10-11	11-12	12-13	13-14	14-15	15-16	16-17	17-18

Pooled Cohort Equations for ASCVD – individuals with hypertension

Reclassification table

Original equation	Re-estimated equation: **<1.25%**	**1.25 – 2.49%**	**2.50 – 3.74%**	**3.75 - 4.99%**	**5.00 – 7.49%**	**7.50 - 9.99%**	**≥ 10.00%**	**Total**
**<1.25%**	13,586 (7.4%)	20,978 (11.5%)	2,722 (1.5%)	74 (0.0%)	1 (0.0%)	0 (0.0%)	0 (0.0%)	37,361 (20.5%)
**1.25 – 2.49%**	5 (0.0%)	5,435 (3.0%)	16,947 (9.3%)	7,278 (4.0%)	1,550 (0.8%)	28 (0.0%)	0 (0.0%)	31,243 (17.1%)
**2.50 – 3.74%**	0 (0.0%)	99 (0.1%)	2,676 (1.5%)	8,810 (4.8%)	9,684 (5.3%)	966 (0.5%)	52 (0.0%)	22,287 (12.2%)
**3.75 - 4.99%**	0 (0.0%)	11 (0.0%)	143 (0.1%)	1,822 (1.0%)	10,318 (5.6%)	4,398 (2.4%)	643 (0.4%)	17,335 (9.5%)
**5.00 – 7.49%**	0 (0.0%)	1 (0.0%)	15 (0.0%)	258 (0.1%)	5,982 (3.3%)	11,499 (6.3%)	7,236 (4.0%)	24,991 (13.7%)
**7.50 - 9.99%**	0 (0.0%)	0 (0.0%)	0 (0.0%)	6 (0.0%)	428 (0.2%)	3,322 (1.8%)	13,006 (7.1%)	16,762 (9.2%)
**≥ 10.00%**	0 (0.0%)	0 (0.0%)	0 (0.0%)	3 (0.0%)	24 (0.0%)	522 (0.3%)	32,127 (17.6%)	32,676 (17.9%)
**Total**	13,591 (7.4%)	26,524 (14.5%)	22,503 (12.3%)	18,251 (10.0%)	27,987 (15.3%)	20,735 (11.4%)	53,064 (29.1%)	182,655 (100%)

**Reclassification**	**NO: 35.6%**	**UP: 63.6%**	**DOWN: 0.8%**

5-year Kaplan Meier estimate of general CVD with 95% confidence intervals

Original equation	<1.25%	1.25 – 2.49%	2.50 – 3.74%	3.75 - 4.99%	5.00 – 7.49%	7.50 - 9.99%	≥ 10.00%	Total
**<1.25%**	1.04% (0.83, 1.25)	1.91% (1.68, 2.14)	2.16% (1.47, 2.84)					1.61% (1.45, 1.77)
**1.25 – 2.49%**		2.75% (2.20, 3.29)	2.94% (2.62, 3.27)	4.94% (4.34, 5.54)	5.6% (4.31, 6.87)			3.52% (3.27, 3.77)
**2.50 – 3.74%**			3.69% (2.83, 4.54)	4.57% (4.03, 5.11)	5.54% (4.99, 6.09)	10.47% (8.10, 12.78)		5.19% (4.83, 5.55)
**3.75 - 4.99%**			6.68% (0.51, 12.47)	5.14% (3.84, 6.42)	6.15% (5.57, 6.72)	7.74% (6.80, 8.66)	12.02% (9.20, 14.75)	6.70% (6.24, 7.15)
**5.00 – 7.49%**				3.95% (1.33, 6.50)	7.32% (6.50, 8.14)	8.26% (7.65, 8.86)	10.92% (10.06, 11.77)	8.82% (8.39, 9.24)
**7.50 - 9.99%**					8.05% (5.10, 10.91)	9.90% (8.58, 11.19)	12.95% (12.26, 13.64)	12.25% (11.65, 12.85)
**≥ 10.00%**						16.02% (12.13, 19.75)	16.95% (16.45, 17.43)	16.92% (16.43, 17.40)
**Total**	1.04% (0.83, 1.25)	2.08% (1.87, 2.30)	2.97% (2.69, 3.25)	4.74% (4.36, 5.12)	6.17% (5.82, 6.51)	8.68% (8.22, 9.15)	15.07% (14.71, 15.43)	7.68% (7.53, 7.83)

Legend: Observed 5-year risk (in %) for CVD and ASCVD

<1	1-2	2-3	3-4	4-5	5-6	6-7	7-8	8-9	9-10	10-11	11-12	12-13	13-14	14-15	15-16	16-17	17-18

Supplementary references

1 R Core Team. R: A language and environment for statistical computing. *R Foundation for Statistical Computing* https://www.R-project.org/ (2018).

2 SAS Institute Inc. *SAS/STAT®14.2 User's Guide*. 3878-4043 (SAS Institute Inc., 2016).

3 Anderson, K. M., Wilson, P. W., Odell, P. M. & Kannel, W. B. An updated coronary risk profile. A statement for health professionals. *Circulation* **83**, 356-362 (1991).

4 Anderson, K. M., Odell, P. M., Wilson, P. W. & Kannel, W. B. Cardiovascular disease risk profiles. *Am Heart J* **121**, 293-298 (1991).

5 D'Agostino, R. B., Sr. *et al.* General cardiovascular risk profile for use in primary care: The Framingham Heart Study. *Circulation* **117**, 743-753 (2008).

6 Goff, D. C. *et al.* 2013 ACC/AHA guideline on the assessment of cardiovascular risk: A report of the American College of Cardiology/American Heart Association Task Force on Practice Guidelines. *Journal of the American College of Cardiology* **63**, 2935-2959 (2014).

7 Klimont, J. & Baldaszti, E. Oesterreichische Gesundheitsbefragung 2014 - Hauptergebnisse des Austrian Health Interview Survey (ATHIS) und methodische Dokumentation. *Statistik Austria* https://broschuerenservice.sozialministerium.at/Home/Download?publicationId=542 (2015).

8 Statistik Austria. Sterbetafeln. *Statistik Austria* http://www.statistik.at/web_de/statistiken/menschen_und_gesellschaft/bevoelkerung/sterbetafeln/index.html (2019).

9 Statistik Austria. Bevölkerung nach Alter und Geschlecht. https://www.statistik.at/web_de/statistiken/menschen_und_gesellschaft/bevoelkerung/bevoelkerungsstruktur/bevoelkerung_nach_alter_geschlecht/index.html (2020).
